# Unravelling the Properties of Fluorescent Ammonium Salts to Obtain Thixotropic Hydrogels with Antitumoral Activity

**DOI:** 10.1021/acsomega.5c06795

**Published:** 2025-12-23

**Authors:** Floriana Billeci, Miriam Buttacavoli, Emanuela Peri, Salvatore Marullo, Patrizia Cancemi, Francesca D’Anna

**Affiliations:** † Dipartimento STEBICEF, 18998 Università degli Studi di Palermo, Viale delle Scienze, Ed. 17 “S. Cannizzaro”, 90128 Palermo, Italy; ‡ Dipartimento STEBICEF, 18998 Università degli Studi di Palermo, Viale delle Scienze, Ed. 16, 90128 Palermo, Italy

## Abstract

Novel fluorescent and thixotropic hydrogels, based on naphthalimide salts differing for both the cation and anion structure, were obtained and applied as promising bioimaging and antitumoral agents. First, the photophysical behavior of the salts was analyzed through UV–vis and fluorescence investigation at variable solvent and concentration, together with the determination of the relative fluorescence quantum yield in water. Organic salts were also tested as gelators, and the resulting soft materials, obtained in H_2_O, H_2_O/DMSO mixtures, and glycerol, were characterized by rheological measurements and fluorescence and resonance light scattering analyses. Morphology of gel phases was examined via scanning electron microscopy. To assess their therapeutic potential, the salts were tested for cytotoxicity and selectivity against a panel of cancer and normal cell lines using the MTT assay. Their performance as bioimaging agents was also evaluated. Remarkably, all salts exhibited strong fluorescence, and their cytotoxicity effects were closely linked to their chemical structure. Notably, the replacement of bromide with gluconate as an anion significantly enhanced cellular uptake, cytotoxicity, and selectivity toward cancer cells. Release experiments revealed that the mechanism of action of the hydrogel can be ascribed to the release of gelator into aqueous media, enabling the ammonium salts to exert a cytotoxic effect. Collectively, our findings support a mechanism of action in which gluconate-based salts are internalized more efficiently by cancer cells, thereby triggering oxidative stress, mitochondrial dysfunction, and apoptotic cell death. These results highlight gluconate-based salts as dual-function materials with promising applications in both cancer therapy and bioimaging.

## Graphical abstract

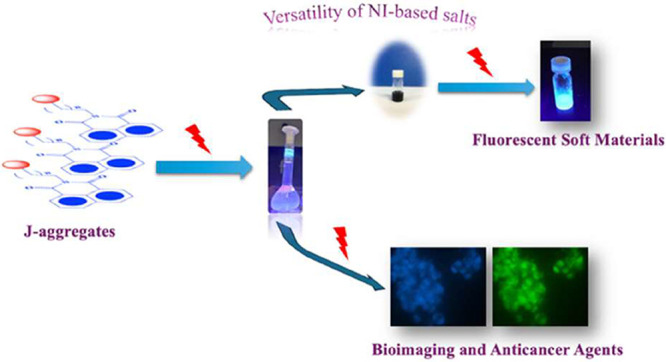

## Introduction

One of the main challenges of modern society is the fight against cancer. According to a 2024 report by the World Health Organization, cancer is one of the top four leading causes of death among people aged 30 years and over worldwide, alongside cardiovascular disease, chronic respiratory disease, and diabetes.^1^ This explains the surge of interest in developing cancer treatments that are both rapid and targeted. In this context, various delivery systems have been explored to efficiently transport anticancer drugs, including polymers,^2,3^ dendrimers,^4,5^ and liposomes.^6,7^ However, these systems have two main limitations: (i) the significant synthetic effort needed to obtain systems with the desired properties and (ii) the low drug loading capacity and the challenging release mechanisms, which often reduce their overall efficiency.

To overcome the above issues, supramolecular hydrogels, formed by low molecular weight gelators (LMWGs), could represent valuable alternatives.^8,9^ Hydrogels are soft materials formed by self-assembly processes, occurring thanks to the establishment of supramolecular interactions among small organic molecules.^8,10–12^ These processes lead to the formation of a 3D network that, by the action of capillary forces, is able to trap the solvent, giving rise to soft materials with intermediate properties between solid and liquid phases. As supramolecular systems, they offer different advantages, among which notable ones are the reversibility of the interactions and their stimuli-responsive nature.^10,13^ This latter property has been boosted during the years of their application in therapeutics and drug delivery systems.^14–16^ Their potential further increases if the LMWG is also the drug molecule, as the supramolecular gel plays the dual role of drug delivery system and therapeutic agent. Furthermore, considering the articulate synthetic pathways frequently featuring pharmaceutically active compounds, a further advantage could be due to the possibility of using drug/gelator systems based on ionic species, such as organic salts. Indeed, notwithstanding that it is frequently said that gelators are serendipitously identified, the use of organic salts and the possibility to easily act on the hydrophobic/hydrophilic balance in the structure, making small changes on the cation or anion structure, represent a useful tool.

This balance is universally recognized as one of the main factors operating in gel phase formation in water^17,18^ and the same factor also affects quantitative structure–activity relationships, consequently determining the potency of a drug molecule.^19,20^

The strategy of using organic salts to obtain hydrogels has been recently used to prepare systems with anticancer activity against melanoma BF16-F10 cells^21^ and metallogels for drug delivery.^22^ In the framework of our interest in studying properties and applications of supramolecular gels,^23^ we have largely used organic salts as LMWGs to gel water,^24^ organic solvents,^25^ and ionic liquids.^26^ In particular, in the case of hydrogels, we have demonstrated that fluorescent imidazolium salts based on 1,8-naphthalimide are able to behave as theranostic agents on cancer cells, keeping their biological activity also in gel phase.^27^ More recently, we have reported data about gel phases formed by folate-based ammonium salts acting as targeted therapeutic agents.^28^

On the grounds of the above considerations, we prepared some 1,8-naphthalimide-based organic salts, featured by the presence of ammonium heads and differing in the length of the alkyl chain or the spacer on the cation, as well as for the nature of the anion (Scheme 1).

**Scheme 1:**
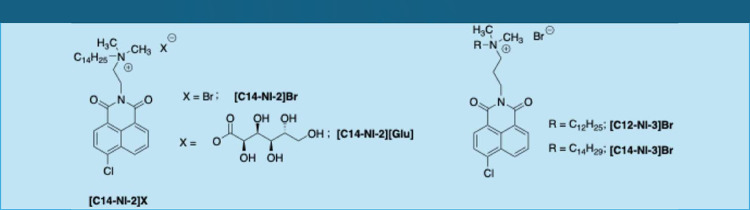
Structures of Used Gelators

Keeping the same efficient fluorophore unit of imidazolium salts but changing the nature of the cationic head could induce significant variations not only in the self-assembly and gelation ability but also in the biological properties of the tested compounds. Indeed, it is well-known that small changes in the structure of biologically active molecules can induce significant variations not only in the potency but also in the activity. This is the reason why, notwithstanding the structural relationship of the salts explored in this paper with the ones investigated in some previous studies, modifications considered in the cationic head, spacer, and alkyl chain length can give useful and valuable insights into their activity. On the other hand, such structural variations can also induce dramatic changes in the aggregation propensity, pathway, and morphologies, as widely reported in the literature for π-conjugated molecules^29,30^ or organic salts.^31^

From now on, the salts used in this work will be indicated as **[Cn-NI-m]­[X]**, where *n* stands for the number of carbon atoms on the alkyl chain and *m* for the ones on the spacer between the aromatic nucleus and the ammonium head. We synthesized dodecyl- and tetradecylammonium derivatives to assess the role played by the alkyl chain length on both the formation of supramolecular gel phases and biological activity. The latter was also evaluated as a function of the length of the spacer joining the naphthalimide nucleus with the ammonium head and going from ethyl to propyl spacers.

As for the anion, we tested the bromide and gluconate derivatives. The latter was chosen for its well-known capability to favor the uptake by cancer cell lines.^32^ One of the main aims of the work is the use of organic salts as bioimaging and antiproliferative agents. Consequently, the absorption and fluorescence behavior of the organic salts was analyzed in water, water/DMSO mixtures at different percentages, TRIS (1×) buffer, and TRIS (1×)/DMSO (90:10; v:v), which proved solvent systems supporting gelation (see later). Furthermore, the emission quantum yield (ϕ), as a function of the organic salts or solvent nature, was determined.

The further step of the work was the investigation of the gelation ability of the salts, tested in biocompatible solvents, like water, aqueous buffers (TRIS 1×), and their mixtures with DMSO and glycerol. Supramolecular gel phases were first characterized by determining the critical gelation concentration (CGC), i.e., the smaller amount of gelator needed to have gel phase formation, and the gel–sol transition temperature (*T*_gel_). Gel phase formation was studied by performing both opacity and resonance light scattering (RLS) investigations to gain insights about gelation time, opacity, and size of the aggregates featuring gel phases. The self-repairing ability of the gel phases was evaluated by performing thixotropy and sonotropy tests, whereas their mechanical properties were assessed through rheology measurements. The emission behavior of gel phases was analyzed, and their emission spectra were compared with the one of the corresponding hot solutions. Finally, supramolecular gel phases were also characterized for their morphology using scanning electron microscopy (SEM) and fluorescence microscopy.

Organic salts, first tested for their cytotoxicity and selectivity activity using MTT assay, toward a panel of cancer and normal cell lines were also evaluated as bioimaging agents.

Interestingly, all of the tested salts showed satisfactory fluorescent properties and were able to induce cytotoxicity, according to their chemical properties. Notably, the replacement of bromide with gluconate as an anion significantly enhanced cellular uptake, cytotoxicity, and selectivity toward cancer cells.

Finally, to obtain insights about the possible mechanism of action, functional assays and Western blotting investigations were performed, using the SK-MEL 28 cell line. Our results suggested a strong induction of reactive oxygen species (ROS) and a pronounced loss of the mitochondrial membrane potential. AO/EB staining revealed a predominantly apoptotic phenotype—membrane blebbing, chromatin condensation, and EB-positive nuclei—although a minority of necrotic cells was also observed. Western blot analyses showed concomitant downregulation of Annexin V and upregulation of caspase-7, especially in [C14–NI-2]­[Glu]-treated cells, indicating the progression to late-stage apoptosis. In summary, gluconate-based salts are internalized more efficiently by cancer cells, where they induce oxidative stress, disrupt mitochondrial function, and activate apoptotic pathways, with necrosis occurring only in a minority of cells. To further investigate the action mechanism of the gels, the possible release of a gelator from the hydrogel into an aqueous buffer was studied and quantified.

Collectively, our results suggest that fluorescent ammonium salts as components of thixotropic hydrogels can provide a highly efficient yet versatile platform for their biomedical applications as bioimaging and antitumoral agents.

## Experimental Section

4-Chloro-1,8-naphthalic anhydride, *N,N*-dimethylethylenediamine, 1-bromododecane, 1-bromotetradecane, *N,N*-dimethyl-1,3-propanediamine, sodium gluconate, Amberlite IRA-400 (Chloride form), and Amberlite IR-120 were obtained from commercial sources and used without further purification. Dichloromethane, toluene, DMSO, diethyl ether, glycerol, and 1× TRIS buffer solution were purchased and used as received. Ultrapure water was used for spectroscopic measurements.

### UV–Vis and Fluorescence Spectroscopy Measurements

Samples for UV–vis and fluorescence spectroscopy were prepared by dilution of stock solutions of salt in the sample solvent. UV–vis spectra were recorded at 25 °C on a Beckmann DU800 spectrophotometer equipped with a Peltier temperature controller, employing quartz cuvettes with a 1 cm optical path.

Samples for fluorescence spectroscopy were degassed prior to measurement. Spectra were recorded with a JASCO spectrofluorometer using quartz cuvettes with a 0.2 cm optical path, and λexc was set at the maximum absorbance wavelength.

### Fluorescence Quantum Yield

Relative fluorescence quantum yields were determined by a reported procedure.^33^ Quantum yields were determined at 25 °C, relative to 9,10-diphenylanthracene in ethanol, employing the standard quantum yield values reported in the literature.^34^

In particular, the relative quantum yield was calculated according to eq 1:

$$\varphi (x)=\left[\left(\frac{A_{s}}{A_{x}}\right)\times \left(\frac{F_{x}}{F_{s}}\right)\times {\left(\frac{n_{x}}{n_{s}}\right)}^{2}\right]\times \varphi_{s}$$ 1

where *A*_s_ and *A*_x_ are the absorbance values of the standard and sample at the excitation wavelength, *F*_s_ and *F*_x_ represent the area of emission peaks corresponding to the standard and the sample, *n*_x_ and *n*_s_ are the refraction indices of the solvents, and ϕ_s_ is the standard emission quantum yield.

### Gelation Tests

The suitable amount of salt was weighed in a screw-capped vial (diameter 1 cm) together with the appropriate solvent (≈250 mg), and the mixture was heated at 80 °C for 1 h, under magnetic stirring, until complete dissolution of the salt. Subsequently, the vial was kept at 4 °C overnight. Gel formation was assessed by the tube inversion test.^35^

### *T*_gel_ Determination

*T*_gel_ was determined by the falling drop method.^36^ A vial containing the preformed gel was placed upside down in a water bath. The bath temperature was raised gradually (1 °C/min) until the gel collapsed, and flow was observed. *T*_gel_ values were reproducible at 1 °C.

### Thixotropy and Sonotropy Tests

The gels were subjected to two different external stimuli. The mechanical stimulus involved stirring the gel phase at 1000 rpm for 5 min using a stir bar (length = 8 mm, height = 3 mm). The sonotropic behavior of the gel phases was tested by irradiating in an ultrasound water bath for 5 min with a power of 200 W and a frequency of 45 kHz. Thereafter, the materials were stored at room temperature overnight. When the samples were stable to the tube inversion test, the gels were defined as thixotropic or sonotropic, respectively.

### RLS Measurements

RLS measurements were carried out with a spectrofluorometer employing a synchronous scanning mode in which the emission and excitation monochromators were preset to identical wavelengths. RLS spectra were recorded from 300 to 700 nm, with both excitation and emission slit widths set at 2.5 nm. Spectra were obtained after 15 accumulations.

### Rheological Measurements

Rheological measurements were carried out on a strain-controlled rheometer equipped with a Peltier temperature controller and a plate–plate tool. Strain and frequency sweep measurements were carried out at 25 °C on three different aliquots of gels within the linear viscoelastic region. In particular, strain sweeps were performed at a frequency of 1 rad/s, while frequency sweeps were performed at a fixed oscillation strain of 0.1%. Thixotropy measurements were carried out by subjecting the sample alternatively to nondestructive strain (γ = 0.1%) for 3 min and to destructive strain (γ = 50%) for 3 min. This sequence was repeated 3 times. Angular frequency was maintained at ω = 1 rad/s, while the temperature was kept at 25 °C.

### SEM Images

SEM images were acquired on the relevant xerogels. Samples for the water-containing gels were obtained by casting the gel into an aluminum stub and removing the solvent under reduced pressure. The sample for the glycerol-based gel was obtained by casting the gel into an aluminum stub and washing it with ethyl acetate to remove the solvent, according to a published procedure.^37^ SEM images were obtained on a PRO X PHENOM electronic scanning microscope, operating at 15 kV, and were acquired at ATeN Center of the University of Palermo—Laboratorio di Preparazione e Analisi di Biomateriali.

### Gelator Release from Gels

400 mg of hydrogel formed by [C14–NI-2]­Br, 4 wt % in H_2_O, was incubated at 37 °C casting 25 mL of PBS buffer. At suitable times, 500 μL of supernatant solution was removed to be spectrophotometrically analyzed by controlling the gelator peak at 343 nm, simultaneously refilled with 500 μL of the same solvent, and prewarmed at 37 °C. In this way, alterations of the final concentration of the gelator in the supernatant solution were minimized. The concentration of the gelator was determined with a calibration curve previously obtained in the PBS buffer.

### Cell Cultures and Treatments

The HCT-116 (CCL-247) (colon cancer), HeLa (CRM-CCL-2) (cervical cancer), MDA-MB-231 (HTB-26) (breast cancer), SK-MEL 28 (HTB-72) (melanoma), and the normal cell lines IMR-90 (CCL-186) (pulmonary fibroblasts) and hTERT RPE-1 (CRL-4000) (epithelial) were purchased from the American Type Culture Collection (ATCC, Manassas, VA, USA) and were maintained in Dulbecco’s modified Eagle medium (DMEM) (Gibco, Paisley, UK), supplemented with 10% heat-inactivated fetal bovine serum, 100 U/mL penicillin, and 100 μg/mL streptomycin, at 37 °C and 5% CO_2_, as already described.^38–41^ Overall, for the experiments, the cell lines were used at the following passage numbers: HCT-116 (passage between 27 and 36), HeLa (passage between 30 and 34), MDA-MB-231 (passage between 35 and 40), SK-MEL 28 (passage between 22 and 31), IMR-90 (passage between 9 and 11), and hTERT RPE-1 (passage between 15 and 17).

### MTT Viability Assay

Cells were plated at 5 × 10^3^ cells/well in 96-well plates and incubated for 24 h before treatment. Stock solutions of H_2_O dissolved salts (5 × 10^–4^ M) were diluted to the desired concentrations (100, 25, 6.25, 1.56, 0.39, 0.097 μM) in the culture medium and added in triplicate to the wells for a further 24 h. After incubation, 20 μL of 5 mg mL^−1^ of thiazolyl blue tetrazolium bromide (Merck, Darmstadt, Germany) in phosphate buffer saline (PBS) was added to each well in the dark and incubated for further 2 h at 37 °C. After removing the medium containing MTT and washing it with PBS three times, 100 μL of DMSO was added to each well to dissolve formazan. The absorbance was recorded at 570 nm using a 96-well plate reader (Spark 20 M Tecan Trading AG, Switzerland). The percentage of cell viability compared to untreated control cells was calculated after subtraction of the blank. IC_50_ values were calculated using GraphPad Prism software by fitting the dose–response curves with a sigmoidal model (log­[inhibitor] vs normalized response, variable slope). In this model, the data are normalized so that the curve runs from 100 to 0%. The IC_50_ is defined as the inhibitor concentration that produces a response halfway between the top and bottom plateaus. The regression equation applied by the software is *Y* = 100/(1 + 10^((Log IC_50_ – *X*) × HillSlope)^), where *Y* represents the normalized response, *X* the logarithm of the inhibitor concentration, IC_50_ the half-maximal inhibitory concentration, and HillSlope describes the steepness of the curve around the IC_50_. Unlike fixed-slope models, the variable slope model allows HillSlope to be fitted from the data, thus providing a more accurate description of the inhibition profile. Each result was the mean value of three different experiments performed in triplicate.

### Morphological Assessment by Phase Contrast Inverted Microscope and Fluorescence Microscopy

Cells were seeded on a coverslip in 12- or 24-well plates at a density of 2.5 × 10^4^ cells/well. After 24 h, cells were treated for 1, 6, or 24 h with appropriate concentrations of selected organic salts. Morphology was observed under a phase contrast inverted microscope or immediately observed under a fluorescent microscope (Carl Zeiss, Oberkochen, Germany) at 400 or 630× magnification. In detail, after 24 h of treatments with the IC_50_ concentration of gluconate-based salts, for AO/EB staining, the cells were washed twice with PBS and stained for a few min with 200 μL of the Acridine Orange (100 μg/mL), ethidium bromide (100 μg/mL) mixture (1:1, v/v). For 2′,7′-dichlorodihydrofluorescein diacetate (DCFH-DA) and JC-1 staining, the cells were incubated for 30 min at 37 °C with 20 μM DCFH-DA (Merk Life Science S.r.l., Merck KGaA, Germany) or 5 μg/mL JC-1 (Enzo Life Science, Farmingdale, USA). Subsequently, the coverslips were washed twice with PBS and immediately observed under the fluorescent microscopy (Carl Zeiss, Oberkochen, Germany) at 630× magnification.

### Western Blotting

SK-MEL 28 cells were seeded in dish plates, grown until 70% confluence, and then treated for 24 h with IC_50_ of selected organic salts. After washing with PBS, cells were carefully scraped and incubated on ice for 30 min in RIPA buffer. The total cellular lysate was centrifuged at 12,000 rpm for 20 min to clear cell debris, and protein concentration was determined by Bradford assay, as already reported.^42–44^ Protein samples (20 μg/lane) were subjected to SDS polyacrylamide gel electrophoresis and then transferred to a nitrocellulose membrane (HyBond ECL, Amersham) and stained with Ponceau S. Membranes were probed using a mouse monoclonal antibody for Actin-β (Santa Cruz, CA, USA, 1:3000) and ANX- 5 (Santa Cruz, CA, USA, 1:500), a rabbit polyclonal for Cleaved CASP-7 (Cell Signaling, 1:500). Following incubation with the appropriate peroxidase-linked antibody, the reaction was revealed by the ECL detection system, using the ChemiDoc MP System (Biorad, Milano, Italy). The correct protein loading was ascertained by immunoblotting for Actin-β. Band quantification was performed using ImageJ software.

## Results and Discussion

### Synthesis of Gelators

Organic salts were synthesized using a previously reported procedure.^27,45^ In the first step, we prepared the neutral precursor **[NI-n]** through the reaction between 4-chloro-1,8-naphthalic anhydride and *N,N*-dimethyl-1,3-propanediamine or *N,N*-dimethylethylenediamine (Scheme 2).

**Scheme 2:**
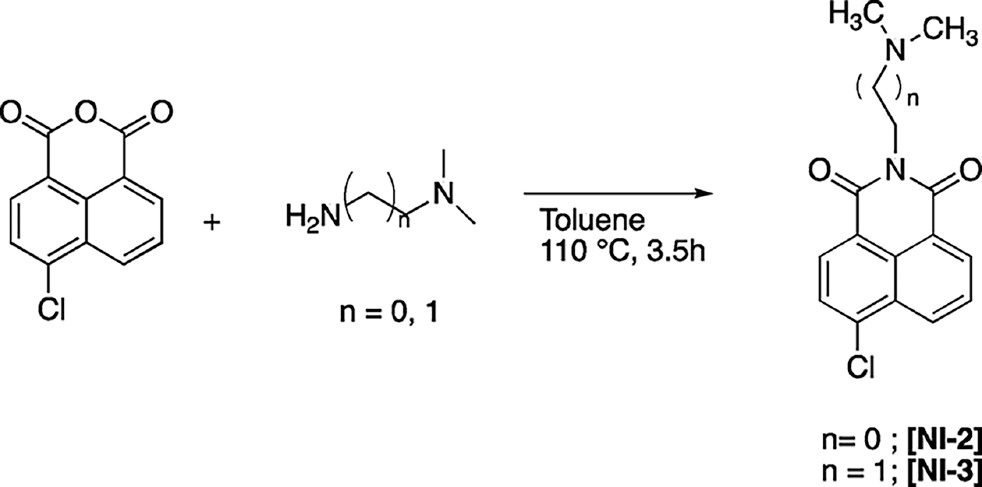
Schematic Representation of the Synthesis of **[NI-n]** Precursors

These precursors were subsequently alkylated with suitable alkyl bromide (Scheme 3).

**Scheme 3:**
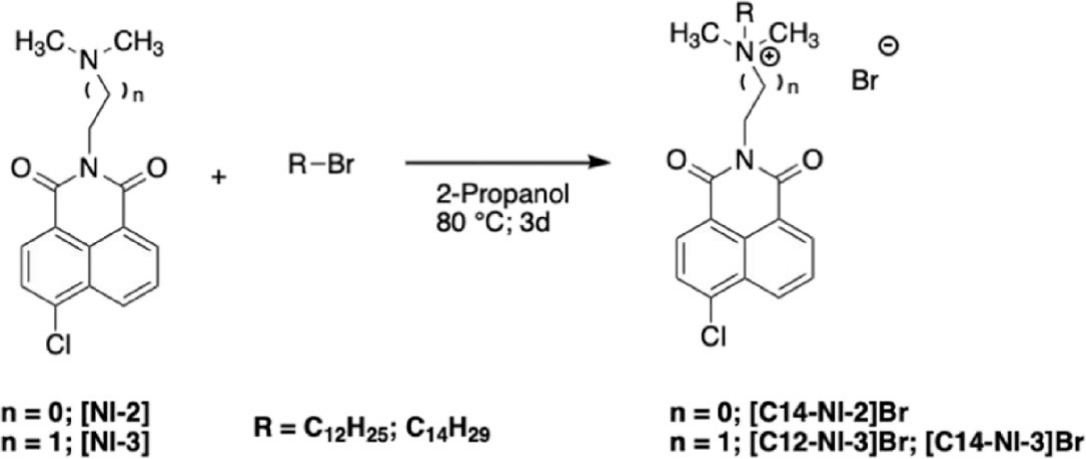
Schematic Representation of the Synthesis of Ammonium Salts

In the case of **[C14–NI-2]­[Glu]**, the anion exchange was performed using a basic ion-exchange resin such as Amberlite IRA-400, according to a previous reported procedure.^46^

### Photophysical Properties of the Organic Salts in Solution

Absorption and emission spectra of all organic salts that proved to be able to form gel phases (see later) were recorded in water, in buffer solution, and in their binary mixture with DMSO to have insights about photophysical properties, considering their possible application as bioimaging agents. In Figure 1, UV–vis spectra corresponding to **[C14–NI-2]­Br** in H_2_O and H_2_O/DMSO binary mixtures are reported. Spectra relevant to all of the other organic salt solutions are reported in Figure S1, whereas λ_max_ values for both absorption and emission spectra are reported in Table S1.

**Figure 1:**
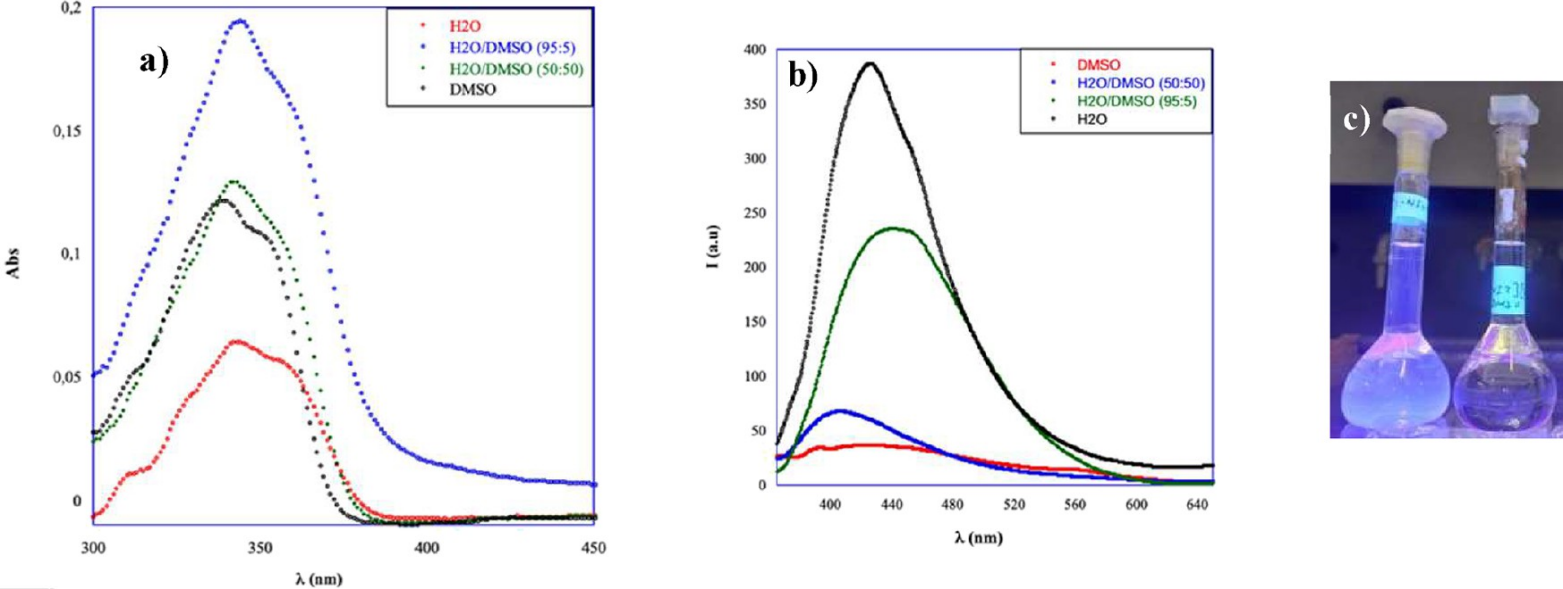
(a) UV–vis spectra of **[C14–NI-2]­Br** (0.0001 M) as a function of solvent nature; (b) emission spectra of **[C14–NI-2]­Br** (0.00001 M) as a function of solvent nature; and (c) picture of irradiated solution in H_2_O (left) and DMSO (right).

Analysis of UV–vis spectra highlights that the absorption of organic salts is affected by the solvent nature. Absorbance values significantly changed on going from DMSO to water solution, but the most significant change was detected in the position of the main absorption band. This exhibited a bathochromic shift, moving from 338 nm in DMSO to 344 nm in a water solution. Changing the nature of the anion and considering **[C14–NI-2]­[Glu]**, we observed the same trend. However, in this case, the observed bathochromic shift proved to be smaller, as the position of the main absorption band moved from 339 nm in DMSO solution to 343 nm in water. On the other hand, the anion being the same (bromide), the spacer lengthening going from **[C14–NI-2]­Br** to **[C14–NI-3]­Br** also induced the same effect with a variation in λ_max_ equal to 6 nm (λ_max_ = 343 and 337 nm in water and DMSO, respectively).

Trends observed with the increase in solvent polarity, going from DMSO to H_2_O, agree with previous report in the literature^47^ and could be indicative of the formation of *J* aggregates.

Analysis of emission spectra, as a function of solvent nature, clearly evidences the positive role exerted by water on the fluorescence emission of both **[C14–NI-2]­Br** and **[C12–NI-3]­Br** (Figure S1 and Table S1). Indeed, in the above cases, emission intensity significantly increased going from DMSO to H_2_O/DMSO binary mixtures to H_2_O. This trend, together with the bathochromic shift observed in the main absorption band on increasing solvent polarity, clearly accounts for the formation of *J* aggregates and for the occurrence of aggregation-induced emission processes. The same trend was also observed comparing the emission intensity of **[C14–NI-2]­Br** in TRIS and TRIS/DMSO (90:10), as the presence of a small percentage of organic solvent halved the emission intensity.

On the grounds of the information gained by recording emission spectra of the organic salts and considering the high emission detected in water solution, we determined the relative emission quantum yield, using 9,10-phenantroline as a standard, in ethanol solution. Data collected are reported in Table 1. Superimposed UV–vis and emission spectra of organic salts and standard are reported in Figure S2, while the ϕ values are reported in Table S2.

**Table 1:** CGC and *T*_gel_ Values as a Function of the Gelator and Solvent Nature

	[C14–NI-2]Br		[C14–NI-2]Glu		[C12–NI-3]Br		[C14–NI-3]Br	
solvent	CGC (%; w/w)	*T*_gel_ (°C)^a^	CGC (%; w/w)	*T*_gel_ (°C)^a^	CGC (%; w/w)	*T*_gel_ (°C)^a^	CGC (%; w/w)	*T*_gel_ (°C)^a^
H_2_O	1.5	53	7.0	36	2.0	42		
H_2_O/DMSO (95:5; v:v)	3.5	47						
H_2_O/DMSO (50:50; v:v)	2.0	65						
TRIS (1×)	3.0	47						
TRIS/DMSO (90:10; v/v)	2.5	48						
Gly	1.0	70			2.0	66	3	42

a *T*_gel_ was reproducible within ±1 °C.

Analysis of the ϕ values shows that it was not affected by the length of the spacer, as accounted for by the comparison between **[C14–NI-2]­Br** and **[C14–NI-3]­Br**. On the other hand, it was significantly affected by the nature of the anion, as it decreased on going from **[C14–NI-2]­Br** to **[C14–NI-2]­[Glu]**. This indicates that the presence of the gluconate anion, featured by a high hydrogen bond donor ability, significantly decreases the emission ability of the salt. Probably, this is a consequence of the more extended solvation shell of the gluconate with respect to the bromide anion, which also induces a significant increase in the polarity of the environment. This hypothesis is also supported by the trend observed with an increase in the alkyl chain length on the cation. Indeed, with the spacer being the same, ϕ values increased going from **[C12–NI-3]­Br** to **[C14–NI-3]­Br**, according to the increase in the hydrophobicity of the substrate. This effect has been previously observed in literature studying aggregation processes in dyes featured by the presence of bulky counterions.^48^

### Gelation Tests

The gelation ability of organic salts was tested in biocompatible solvents, like H_2_O, TRIS (1×) buffer, and its mixture with DMSO at 10% (v/v) and glycerol. When we obtained gel phases, these were white or light-yellow opaque gels (Figure S2).

Gel phases obtained were stable for at least three months at room temperature. Gelation tests were aimed at the determination of the CGC, i.e., the lower amount of gelator needed to have gel phase formation. Furthermore, we also determined the gel–sol transition temperature (*T*_gel_), using the falling drop method.^36^ CGC and *T*_gel_ values, as a function of gelator and solvent nature, are reported in Table 1. Results of gelation tests are reported in Tables S3 and S4 of the SI.

Analysis of data reported in the table shows that CGCs range from 1 up to 7% (w/w). Among tested gelators, **[C14–NI-2]­Br** proved to be the best one. Indeed, it formed gels in most of the solvents used. In general, for the above gelator, CGC increased upon going from water or buffer solutions to corresponding mixtures with DMSO. The lowest CGC value was detected in glycerol. Comparison with data previously reported by us about the gelling ability of corresponding imidazolium salts, **[C14-NIim]­Br**,^27^ evidence a comparable gelling ability of the ammonium salt in water, but a lower tendency to give rise to soft materials formation in H_2_O/DMSO and Tris/DMSO mixtures. Indeed, in water CGCs proved to be comparable (1.5 and 2.0 wt % for **[C14–NI-2]­Br** and **[C14NIim]­Br**) whereas in H_2_O/DMSO and Tris/DMSO significant different ranges were detected (2.5–3.5 wt % for **[C14–NI-2]­Br** and 0.7–1.5 wt % for **[C14NIim]­Br**).

As for gel phases formed in glycerol, CGC increases on going from **[C12–NI-3]­Br** to **[C14–NI-3]­Br**, according to the increase in the alkyl chain length. On the other hand, CGC also increases with the increase in the length of the spacer, going from **[C14–NI-2]­Br** to **[C14–NI-3]­Br**. The above trend, dependent on the alkyl spacer length, was also detected in water solution, though to a minor extent.

Analysis of *T*_gel_, measured at the CGC, shows that melting temperature of the gels ranges from 36 °C, for **[C14–NI-2]­[Glu]** in H_2_O, up to 70 °C for [**C14–NI-2]­Br** in glycerol. However, because of the different CGC values, further analysis cannot be performed. We attempted to determine *T*_gel_ at the common concentration of 4% (w/w), but in these conditions, it was too high to be measured using the falling drop method. The above concentration value was used to perform characterization of the gel phases.

### Rheology Investigation

With the results of gelation tests at hand, we performed rheology investigation to demonstrate the true gel nature of our samples and to have insights about their mechanical behavior. We were able to perform the above analysis with the only exception of **[C14–NI-2]­[Glu]/H**_**2**_ **O**, that proved too feeble and did not resist to the oscillatory action of the rheometer.

We performed frequency and strain sweep measurements. In both cases, we detected typical gel behavior. Indeed, in the frequency sweep investigation, strain being constant, *G*′, the storage modulus representing the solid-like behavior, was always higher than *G*″, the loss modulus indicative of the liquid behavior. Furthermore, in the frequency range investigated, the moduli mentioned above were fairly independent from the frequency. In the case of strain sweep measurements, frequency being constant, at low strain values, we detected the linear viscoelastic region (LVR), in which *G*′ > *G*″, until a strain value corresponding to the inversion of the moduli (γ) and representing the strain needed to induce the gel network breakdown was reached. In Figure 2a,b, plots corresponding to frequency and strain sweep investigations for **[C14–NI-2]­Br/H**_**2**_ **O** are reported. Plots corresponding to all of the other gel phases are reported in Figure S4.

**Figure 2:**
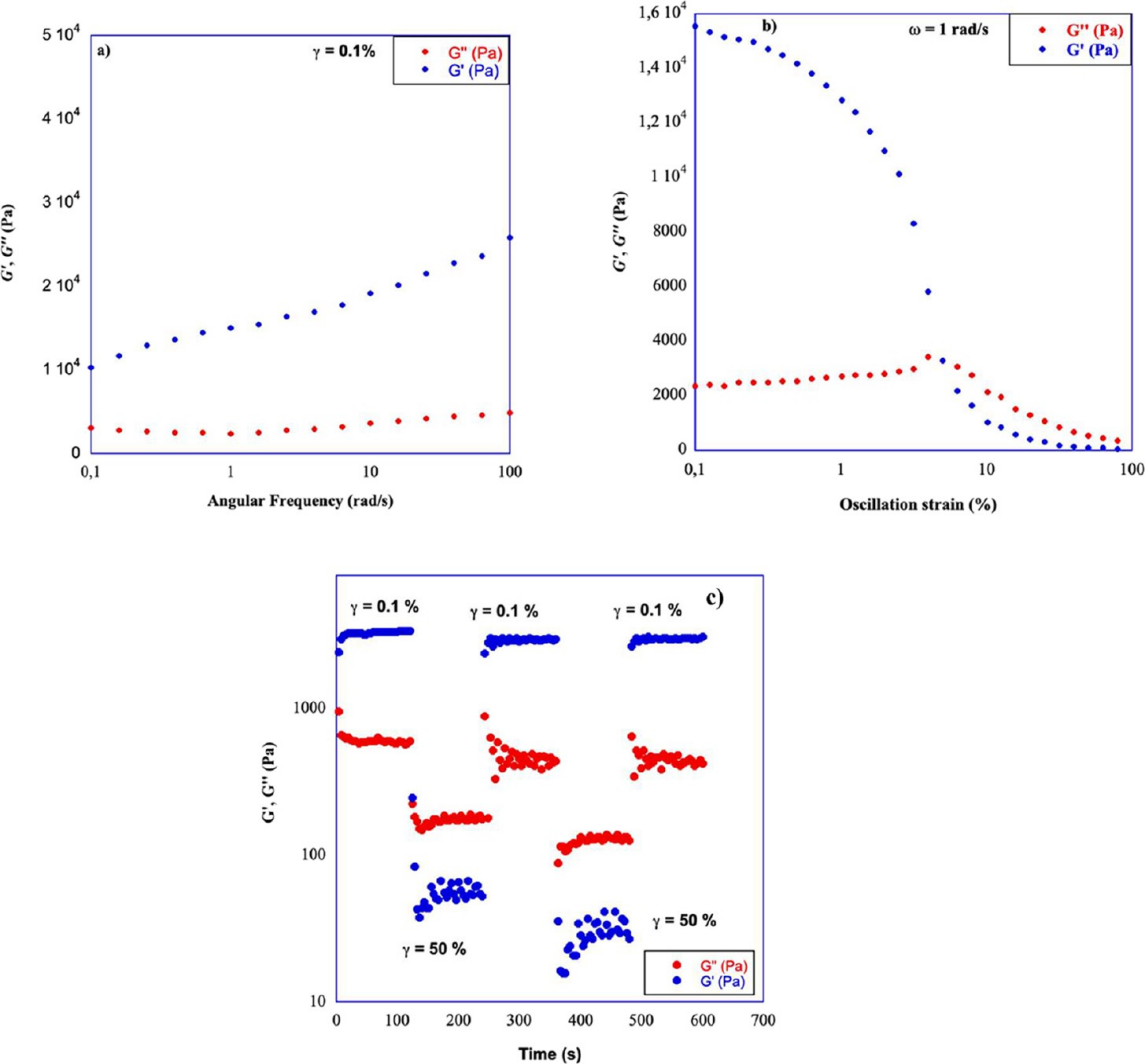
Plot of (a) frequency sweep and (b) strain sweep corresponding to **[C14–NI-2]­Br/H2O** at 4% (w/w); (c) *G*′ and *G*″ at 25 °C as a function of time and application of low (*G*′ > *G*″ regimes) and destructive stain (*G*″ > *G*′ regimes) to **[C14–NI-2]­Br/H**_**2**_ **O/DMSO (95:5)** at 4% wt.

Furthermore, in Table 2, rheological parameters as a function of the gel nature are reported. In the above table, besides *G’* and *G*″ values, also γ (%) and tan δ values are displayed.

**Table 2:** *G*′ and *G*″ at γ = 0.1% and ω = 1 rad s^–1^, tan δ = *G*″/*G*′, and Values of γ at *G*″ = *G*′ for Different Gels (4 wt.%)^a^

gel	*G′* (Pa)	*G″* (Pa)	tan δ	γ (%)
[C14–NI-2]Br/H_2_O	15520 ± 50	2430 ± 80	0.156 ± 0.006	5.2
[C14–NI-2]Br/H_2_O/DMSO (95:5)	2680 ± 90	400 ± 16	0.15 ± 0.01	14 ± 1
[C14–NI-2]Br/H_2_O/DMSO (50:50)	(3.5 ± 0.1) × 10^5^	(5.9 ± 0.1) × 10^4^	0.15 ± 0.01	1.9 ± 0.2
[C14–NI-2]Br/TRIS	3610 ± 15	485 ± 8	0.13 ± 0.01	18 ± 3
[C14–NI-2]Br/TRIS/DMSO (90:10)	5300 ± 200	910 ± 40	0.170 ± 0.003	12 ± 2
[C14–NI-2]Br/Gly	90000 ± 6000	12500 ± 400	0.14 ± 0.013	2.24 ± 0.08
[C14–NI-3]Br/Gly	8900 ± 900	2300 ± 200	0.26 ± 0.01	12 ± 1
[C12–NI-3]Br/Gly	14000 ± 2000	2500 ± 600	0.18 ± 0.01	2.8 ± 0.3
[C12–NI-3]Br/H_2_O	8600 ± 120	5500 ± 300	0.67 ± 0.02	1.0 ± 0.3

a Error limits are based on an average of three different measurements with different aliquots.

As stated above, γ represents the crossover point in the strain sweep plot. On the other hand, tan δ (=*G″*/ *G′*) represents the strength of colloidal forces featuring gel phase. In general, the lower this value, the stronger the colloidal forces. In the present case, tan δ ranges from 0.13 for **[C14–NI-2]­Br/TRIS** up to 0.67 for **[C12–NI-3]/H**_**2**_ **O**, allowing to indicate the last gel as the feeblest one. This latter gel also had the lowest γ value. In all cases, detection of tan δ values significantly lower than 1 indicates that our gel phases were formed thanks to the occurrence of strong supramolecular interactions.

To analyze mechanical features of the gels, a systematic analysis of *G′* values can be performed. *G′* is a measure of the stiffness of the gel, i.e., a measure of the gel’s resistance to deformation. Analysis of collected data shows that *G’* is significantly affected by both gelator and solvent nature. More in detail, *G′* ranged from 2680 Pa for **[C14–NI-2]­Br/H**_**2**_ **O/DMSO** (95:5) up to 350 kPa for **[C14–NI-2]­Br/H**_**2**_ **O/DMSO** (50:50). Then, the presence of DMSO induced a significant increase in the gel stiffness, as accounted for also by the comparison with the value measured for **[C14–NI-2]­Br/H**_**2**_ **O**. On the gelator being the same as **[C12–NI-2]­Br**, the increase in solvent viscosity, going from **[C12–NI-2]­Br/H**_**2**_ **O** to **[C12–NI-2]­Br/Gly**, also induced a significant increase in the gel resistance to deformation.

Negative effects were detected on increasing the ionic strength of the solvent medium and the alkyl spacer length of the gelator. Indeed, in the first case, *G*′ values significantly decreased going from **[C14–NI-2]­Br/H**_**2**_ **O** to **[C14–NI-2]­Br/TRIS**. Similarly, the gel stiffness proved to be an order of magnitude lower, going from **[C14–NI-2]­Br/Gly** to **[C14–NI-3]­Br/Gly**. Probably, in the last case, the increase in conformational flexibility hampers the suitable gelator organization to maximize supramolecular interactions.

### Self-Repairing Ability

Gel phases were investigated for their ability to self-repair after the action of external stimuli, like mechanical stirring or ultrasounds irradiation. The above tests allow us to evaluate the thixotropic or sonotropic behavior of these soft materials. Thixotropy is a very important property of supramolecular gels, that frequently allows their use in drug delivery systems,^49,50^ lubricants^51^ or propellant systems.^52^ We have already observed this kind of property studying features of gel phases formed in water,^53^ ionic liquids^26^ or deep eutectic solvents.^54^ Results collected are reported in Table S5.

Analysis of the results shows that, with the only exception of **[C14–NI-2]­Br/H**_**2**_ **O**, all gels formed by the gelator described above proved to be thixotropic. In the case of the above-mentioned hydrogel, mechanical stirring was not able to induce the breakdown of the gel network. Independently from the gelator nature, soft materials formed in glycerol were not affected by the mechanical stirring. Among the other gel phases, **[C12–NI-3]­Br/H**_**2**_ **O** and **[C14–NI-2]­[Glu]/H**_**2**_ **O** were not able to restore the gelatinous network after mechanical stirring.

On the grounds of the above results, the ability of gel phases to self-repair after the action of a mechanical stimulus was further verified by performing rheological investigation. Gel phases that positively responded to the mechanical stirring were alternatively subjected to nondestructive strain (γ = 0.1%) for 3 min and to destructive strain (γ = 50%) for 3 min at 25 °C.

In Figure 2c, typical thixotropic behavior detected for **[C14–NI-2]­Br/H**_**2**_ **O/DMSO** (95:5) is reported, whereas plots corresponding to all of the other gel phases are reported in Figure S6. For a better evaluation, in Table 3, the percentage of storage modulus recovery as a function of gel nature are reported.

**Table 3:** Storage Modulus Recovery for Different Hydrogels at 4% wt^a^

gel	storage modulus recovery (%)
**[C14–NI-2]Br/H**_**2**_ **O/DMSO** (95:5)	90 (I), 90 (II)
**[C14–NI-2]Br/H**_**2**_ **O/DMSO** (50:50)	99 (I), 98 (II)
**[C14–NI-2]Br/TRIS**	100 (I), 100 (II)
**[C14–NI-2]Br/TRIS/DMSO** (90:10)	88 (I), 90 (II)
**[C14–NI-2]Br/Gly**	72 (I), 65 (II)

a The number of cycles is indicated in brackets.

Analysis of *G*′ and *G*″ moduli evolution, as a function of the applied strain, evidence that in all cases, gel phases were able to reform over three cycles. On the other hand, analysis of recovery percentage sheds light on the positive role played by the presence of organic cosolvents in the gel. Indeed, gelator being the same, i.e., **[C14–NI-2]­Br**, the gel phase became thixotropic going from H_2_O to H_2_O/DMSO binary mixtures, and in both mixtures, they were able to almost recover the initial value of the modulus. This result well agrees with data obtained from RLS investigation, evidencing a significant increase in the size of the aggregates (see later) due to gradual increase in the amount of DMSO in the solvent system. Also, the increase in the ionic strength, going from water to TRIS buffer, favors the formation of a thixotropic gel and also in this case the soft material was able to gain the initial stiffness over two cycles. However, to addition of DMSO to buffer solution, going from **[C14–NI-2]­Br/TRIS to [C14–NI-2]­Br/TRIS/DMSO** (90:10), slightly decreased the self-repairing ability of the gel, also if after the second cycle *G*′ value was equal to the 90% of the initial one. The modulus recovery significantly decreases in the case of **[C14–NI-2]­Br/Gly,** but still maintaining the thixotropic behavior.

Finally, analysis of the responses to ultrasound irradiation demonstrates a significantly different behavior. Indeed, only in the case of **[C14–NI-2]­Br/H**_**2**_ **O/DMSO** (50:50), soft materials did not reform after irradiation. In most cases, gels proved to be stable, and only **[C12–NI-3]­Br/H**_**2**_ **O** was able to reform, behaving as a sonotropic material.

### Opacity Investigation

Gel phase formation was studied using two different approaches, opacity and RLS investigation, providing insights about different features of the materials. To this aim, we performed an opacity measurement as a function of time. As reported in the literature, opacity accounts for the crystallinity of the gel phase.^35^ In general, the higher the opacity, the higher the crystallinity of the gel phase.

All samples were prepared as hot solutions, and absorbance values were detected as a function of time. In all cases, we observed a gradual increase in the absorbance until a maximum and constant value was reached. This was considered as a measure of the crystallinity of the gel and the corresponding time was taken as the gelation time, i.e., the time needed to have gel phase formation. At the end of the investigation, this was further verified by the tube inversion test.^55^ Opacity plots for all the other gel phases are reported in Figure S6.

Analysis of opacity values at the equilibrium shows that, with the only exception of **[C14–NI-3]­Br/H**_**2**_ **O**, all gel phases exhibited comparable opacities and, values measured (∼2.0) are indicative of high crystallinity (Table S6). Different considerations can be made as far as gelation time is concerned. Indeed, the above parameter ranges from 28 to 30 s, in the case of **[C14–NI-2]­Br/H**_**2**_ **O** and **[C14–NI-2]­[Glu]/H**_**2**_ **O**, up to 420 s in the case of **[C12–NI-3]­Br/H**_**2**_ **O** (Table S4). However, gel phase formation occurred fast, independently from the alkyl chain or spacer length, the anion nature, or solvent used. A deeper analysis of the above values testifies that, gelator being the same, i.e., **[C14–NI-2]­Br**, gelation time gradually increases going from H_2_O to H_2_O/DMSO (95:5; v/v) or H_2_O/DMSO (50:50; v/v) or going from TRIS buffer to its binary mixture with DMSO. On the other hand, in the case of **[C12–NI-2]­Br**, gel phase formation also slowed down going from H_2_O to Gly.

As far as the alkyl chain length is concerned, in water solution, going from **[C14–NI-3]­Br** to **[C12–NI-3]­Br**, analysis of the results evidenced the significant role played by hydrophobic interactions, as the higher the hydrophobicity, the faster the gelation process. This trend is also confirmed by the comparison between data collected for **[C12–NI-3]­Br/Gly** and data collected for **[C14–NI-3]­Br/Gly**. Probably, in this case, the increase in van der Waals interactions accounts for the observed trend.

Finally, comparison between gelation times corresponding to **[C14–NI-2]­Br/H**_**2**_ **O** and **[C14–NI-2]­[Glu]/H**_**2**_ **O** indicates that changing the anion nature and, in particular, increasing the hydrogen bond donor ability of the anion did not affect the gelation time, probably evidencing that, in water solution, hydrophobic interactions play a major role with respect to the hydrogen bond in determining the outcome of the gelation time.

### RLS Investigation

Gel phase formation was also investigated by performing RLS measurements. RLS is a technique able to give insights into the presence of aggregates in solution formed by chromophoric units. It allows having information about the size of the aggregates, as the intensity of the scattered light is related to their square volume.^56^ It has been used to investigate systems with different features, like porphyrins,^57^ ionic liquids,^58,59^ deep eutectic solvents^60^ and also supramolecular gels.^61^ In the present case, the investigation was performed as a function of time, to also have information about the mechanism of the gelation process.

Plots of I_RLS_ as a function of time for all of the other gel phases are reported in Figure S7.

Analysis of the obtained plots shows that, with the only exception of [**C14–NI-3]­Br/H**_**2**_ **O/DMSO** (95:5), **[C14–NI-2]­Br/Gly** and **[C12–NI-3]­Br/Gly**, in all the other cases I_RLS_ increased as a function of the time until a constant values, representative of the gel phase formation, was reached. In the above-mentioned cases, a different kinetic trace was obtained, as before gel formation a maximum intensity value was reached that subsequently decreases to a constant value. We have previously observed a similar behavior studying the gelling ability of some diimidazolium salts in alcohol solution^53,62^ and, according to previous reports in literature,^53^ it was ascribed to the first formation of larger three-dimensional aggregates that subsequently contract proceeding through fiber stacking into bundles.^35^ In all cases, at the end of the process, gel phase formation was assessed by the inversion test.,^35^ I_RLS_ values measured after gel phase formation are reported in Table S6.

In general, intensity values ranged from 40 au up to 760 au The above parameter significantly increased going from H_2_O to H_2_O/DMSO mixtures, as accounted for by data collected for **[C14–NI-2]­Br** in H_2_O and H_2_O/DMSO (50:50), probably indicating that the presence of an organic solvent favors the formation of a more extended network. In support of the above hypothesis, the same parameter also increased upon going from **[C14–NI-3]­Br/H**_**2**_ **O** to **[C14–NI-3]­Br/Gly**.

A relevant effect was also detected, changing the nature of the anion. Indeed, more extended aggregates were formed by **[C14–NI-2]­[Glu]/H_2_O** than by **[C14–NI-2]­Br/H_2_O**. Probably, the anion able to form a hydrogen bond network also favored the formation of larger aggregates. Finally, the last factor to be considered is that concerning the lengthening of the alkyl chain or alkyl spacer. In both cases, comparing data collected for **[C12–NI-3]­Br/Gly** and **[C14–NI-3]­Br/Gly** and the ones collected for **[C14–NI-2]­Br/Gly** and **[C14–NI-3]­Br/Gly**, a gradual decrease in I_RLS_ values was detected, that could be ascribed to the increase in the conformational flexibility that could hamper the organization in larger systems.

### Emission Behavior of the Gels

On the grounds of the results collected about emission behavior of the salts in solution, and with the aim to verify if emission could be kept also in the gel phase or if it could change because of the aggregation process, we recorded emission spectra of gel phases and the one of the corresponding hot solutions. In Figure 3a, spectra corresponding to **[C14–NI-2]­Br/H**_**2**_ **O** are reported, whereas the ones relevant to all the other gel phases are displayed in Figure S8. In Table S7, emission intensity values at the wavelength of the main band, together with Δλ values detected as a result of the gelation process, are reported.

**Figure 3:**
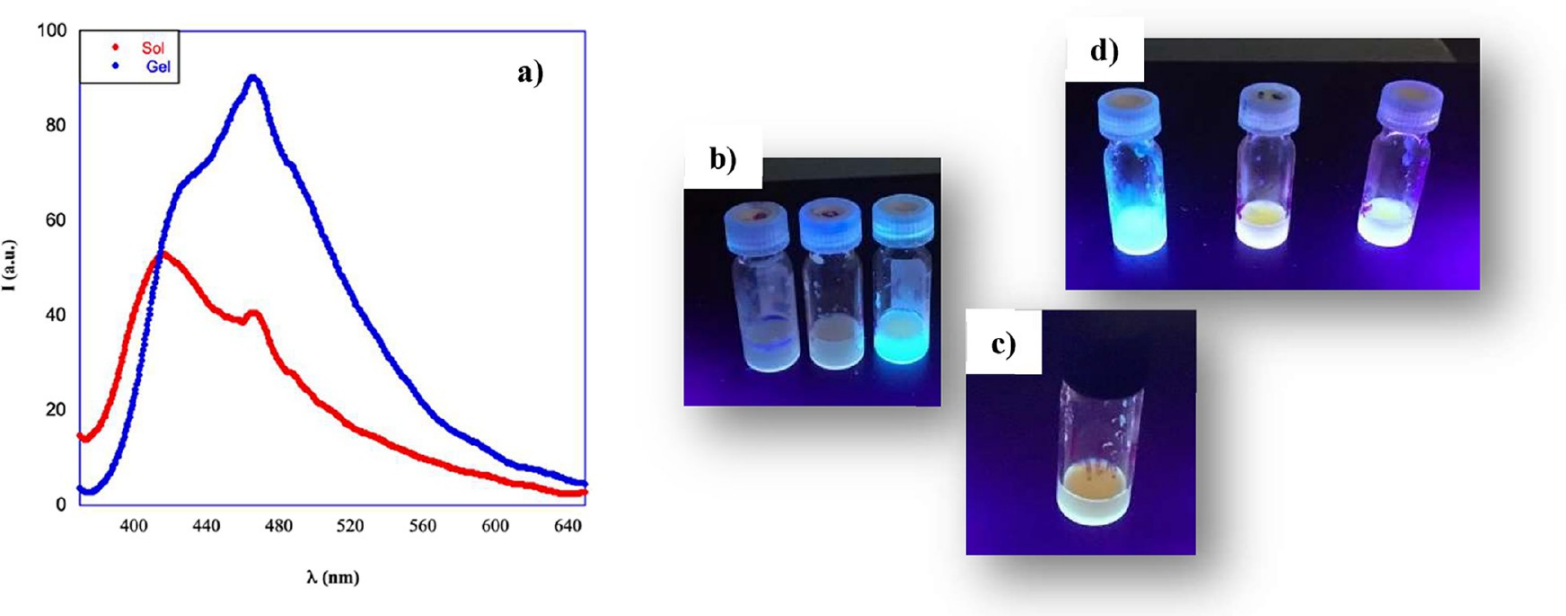
(a) Emission spectra of **[C14–NI-2]­Br/H**_**2**_ **O** at 4 wt % hot solution and gel phase; (b) **[C14–NI-2]­Br** gel at 4 wt % after irradiation at 365 nm in H_2_O (left), H_2_O/DMSO (95:5) (central), H_2_O/DMSO (50:50) (right); (c) **[C14–NI-2]­[Glu]/H2O** gel at 4 wt % after irradiation at 365 nm; (d) **[C14–NI-2]­Br/Gly** (left), **[C14–NI-3]­Br/Gly** (central), **[C12–NI-3]­Br/Gly** (right) gel at 4 wt % after irradiation at 365 nm.

With the only exception of gels formed in glycerol, in all of the other cases, going from the hot solution to the corresponding gel phase, we detected both a significant increase in the emission intensity, together with a change in the position of the band. This result was a clear indication that the gelation process occurred through an aggregation induced emission process that gives rise to the formation of highly emitting aggregates.

Emission intensity changed from ∼10 au for **[C14–NI-3]­Br/Gly** up to 290 a.u. **[C14–NI-2]­Br/Gly**, highlighting a certain role played by the spacer length in determining the gelator organization in the three-dimensional network. Interestingly, the above structural features also affected the size of the aggregates, with the gel phase formed by **[C14–NI-2]­Br** featured by the presence of more extended aggregates (see above), which could be responsible for the higher fluorescence emission.

Emission ability was also influenced by the anion nature and the solvent composition. In the first case, emission intensity significantly decreased going from **[C14–NI-2]­Br/H**_**2**_ **O** to **[C14–NI-2]­[Glu]/H**_**2**_ **O**. On the other hand, as for solvent composition, data collected for **[C14–NI-2]­Br** in H_2_O and H_2_O/DMSO binary mixtures clearly indicate a positive effect due to the presence of the cosolvent, as the emission intensity increased with the amount of DMSO in the gelation solvent. Once again, this result can be ascribed to the increase in the size of the aggregates featuring gel phases, and this effect was also visible because of the gel irradiation (Figure 3b), which accounts for a gradual increase of blue emission with the increase in the DMSO amount.

As previously stated, the gelation process also induced a shift in the main emission band. In particular, the most significant changes were detected for gels formed by H_2_O/DMSO binary mixtures, with a relevant bathochromic shift that parallels the increase in DMSO amount. On the other hand, gelation in glycerol solution induced a significant hypsochromic shift that increased with the increase in the gelator alkyl chain length.

Interestingly, the solvent being the same, the hypsochromic shift was also affected by the anion nature, as accounted for by data collected for **[C14–NI-2]­Br/H**_**2**_ **O** and **[C14–NI-2]­[Glu]/H**_**2**_ **O**. Also in this case, the visual observation after irradiation perfectly supports the above trend (Figure 3c). Indeed, **[C14–NI-2]­Br/H**_**2**_ **O** gave a feeble emission, whereas **[C14–NI-2]­[Glu]/H**_**2**_ **O** seems to give a white emission. The same peculiar behavior was also observed for **[C14–NI-3]­Br/Gly** and **[C12–NI-3]­Br/Gly**.

### Morphology of Gel Phases

Results collected by the analysis of the emission behavior of the gels prompted us to perform a detection of the gel morphology using fluorescence microscopy, employing an excitation range of 300 ms. Images obtained as a function of the different nature of the hydrogels, are reported in Figure 4. In this case, gels formed in water and glycerol were considered because of the different shift observed as a consequence of the gelation process and also of the visual emission behavior recorded after gel phase irradiation (intense blue light for hydrogels and white emission for organogels, see Figure 3b–d).

**Figure 4:**
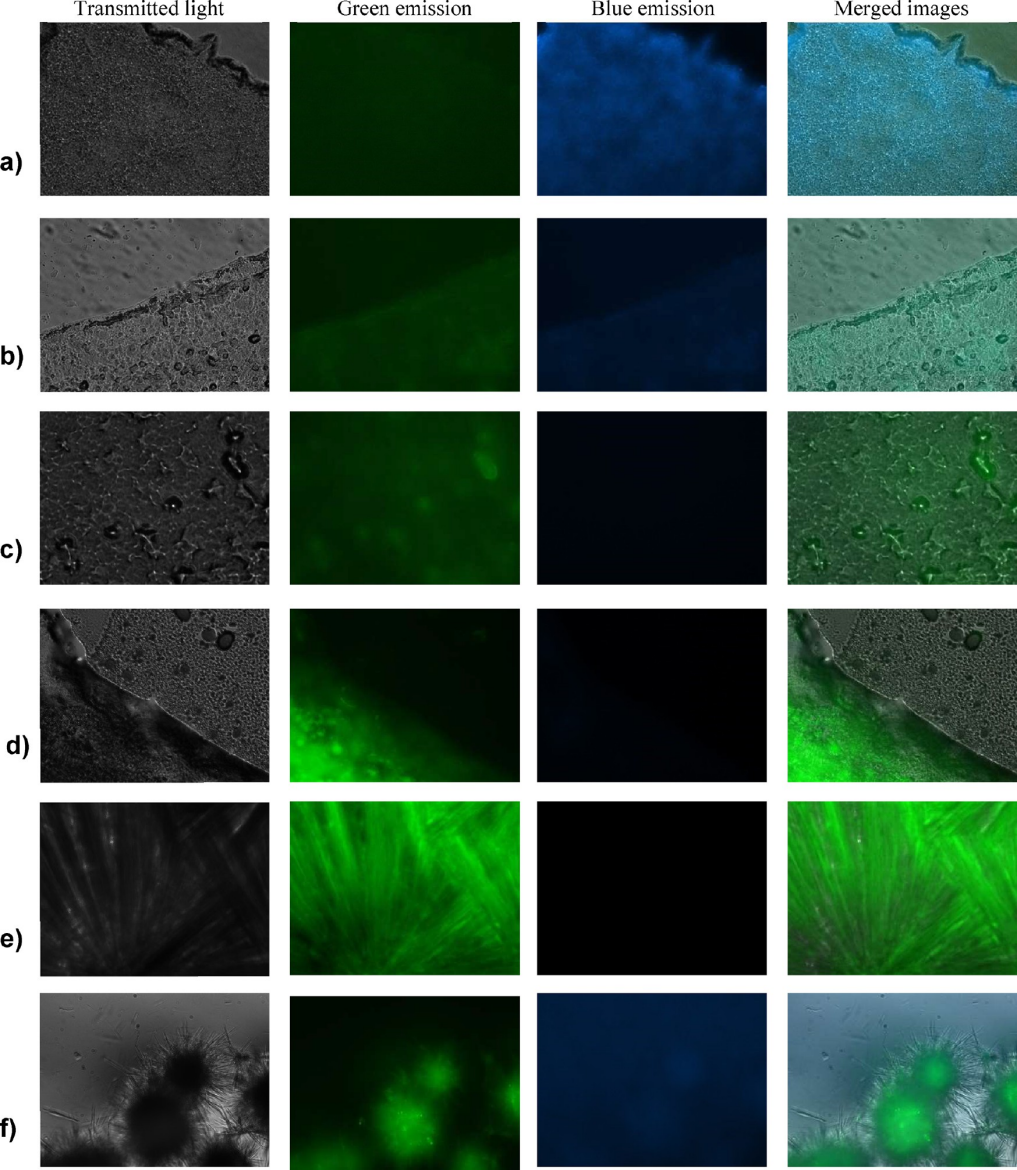
Gel morphology detected at the fluorescence microscopy (excitation range 300 ms): (a) **[C14–NI-2]­Br/Gly**; (b) **[C14–NI-2]­Br/H2O**; (c) **[C14–NI-2]­[Glu]/H**_**2**_ **O**; (d) **[C12–NI-3]­Br/Gly**; (e) **[C12–NI-3]­Br/H2O**; (f) **[C14–NI-3]­Br/Gly**. Images were taken at 200× magnification on a microscope equipped for epifluorescence (Carl Zeiss, Oberkochen, Germany). All samples were prepared at 4 wt %.

Analysis of fluorescence microscopy images supports the hypothesis about the role played by the nature of the gelation solvent in determining both the morphology and emission range. Gels formed by **[C14–NI-2]­Br** in H_2_O and glycerol exhibited a highly compact texture (Figure 4a,b). However, highly intense blue fluorescence emission was detected in the case of **[C14–NI-2]­Br/Gly**, which decreased in the corresponding hydrogel that was instead able to give green fluorescence emission.

The change in the nature of the anion, in the case of **[C14–NI-2]­[Glu]/H**_**2**_ **O**, did not affect the nature of the morphology, but in this case, the hydrogel was able to give only a light green emission (Figure 4c). This result perfectly recalls the trend observed comparing emission spectra of the hot solution and corresponding gel phase (see above). Indeed, in the above-mentioned cases, the gelation process was featured by a significant hypsochromic shift of the main emission band.

The elongation of the alkyl spacer, going from **[C14–NI-2]­Br/Gly** to **[C14–NI-3]­Br/Gly** induced changes both in morphology and fluorescence emission (cf. Figure 4a,f). Indeed, in the last case, the presence of spherulitic aggregates was easily evidenced, and the occurrence of a highly intense green light emission predominated the fluorescence pattern. Once again, the above changes perfectly fit the trend observed by analyzing emission spectra of soft materials, as the hypsochromic shift induced by gelation was larger in the case of **[C14–NI-3]­Br/Gly** than in the case of **[C14–NI-2]­Br/Gly** (Δλ = −6.5 and 16.0 nm, respectively). Interestingly, changes in morphology corroborate the decrease in the size of the aggregates observed, performing an RLS investigation (Figure 5c). Indeed, this accounts for the occurrence of larger aggregates in the soft materials exhibiting a compact texture (**[C14–NI-2]­Br/Gly**) than those in the one showing spherulitic domains (**[C14–NI-3]­Br/Gly**).

**Figure 5:**
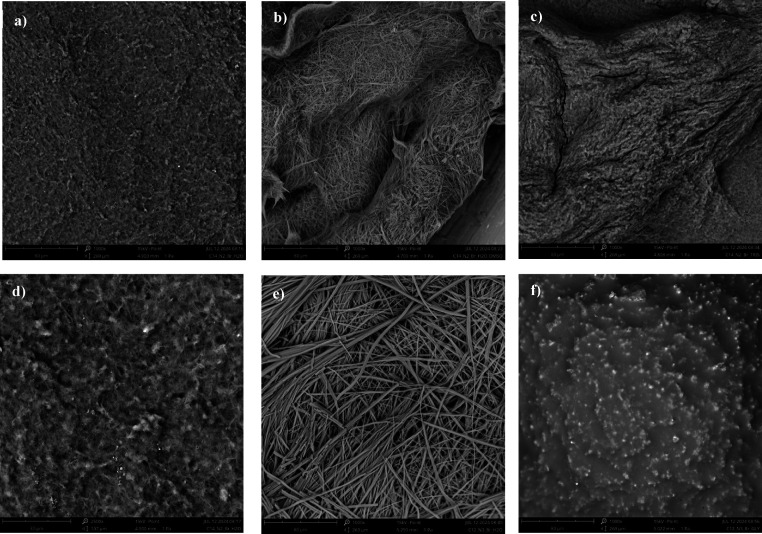
SEM micrographs of hydrogels at 4 wt % (1000×): (a) **[C14–NI-2]­Br/H**_**2**_ **O**; (b) **[C14–NI-2]­Br/H**_**2**_ **O/DMSO (50:50)**; (c) **[C14–NI-2]­Br/TRIS**; (d) [**C14–NI-2]­[Glu]/H**_**2**_ **O**; (e) **[C12–NI-3]­Br/H**_**2**_ **O**; (f) **[C12–NI-3]­Br/Gly**.

Finally, shortening of the alkyl chain, going from **[C14–NI-3]­Br/Gly** to **[C12–NI-3]­Br/Gly** allowed detecting a morphological transition from a spherulitic motif to a compact structure emitting green light, perfectly recalling trend observed as a consequence of the shortening of the alkyl spacer (Figure 4d,f). Interestingly, once again, analysis of I_RLS_ values accounts for the presence of more extended aggregates in the case of **[C12–NI-3]­Br/Gly.** The same emission range was kept in water solution, but with the clear presence of spherulitic aggregates (cfr Figure 4d,e).

To gain more insight into the structural motifs featuring gel phases, some selected samples were also analyzed as xerogels, performing SEM investigation. We know that drying could affect the 3D network of the gels. However, as in all cases, it was performed by slow evaporation at room temperature, and we are confident that only minor perturbations occurred, and different samples can be confidently compared. In this case, we analyzed the effect of solvent nature, considering gels in H_2_O, H_2_O/DMSO binary mixtures and glycerol, together with changes in structural features like the nature of the anion, the alkyl chain and spacer length. SEM micrographs are reported in Figure 5.

Analysis of collected micrographs evidence the significant effect deriving from the solvent nature. Indeed, independently from the anion nature, gels formed in water exhibited a compact texture, as demonstrated by micrographies obtained for **[C14–NI-2]­Br/H_2_O** and **[C14–NI-2]­[Glu]/H**_**2**_ **O** (Figure 5a,d). However, adding DMSO (Figure 8b) as well as increasing the ionic strength due to the preparation in buffer solution (Figure 5c) significantly changed the morphology, and in both cases, the presence of thin fiber-based structures was evidenced. This structural motif becomes predominant in the case of **[C12–NI-3]­Br/H**_**2**_ **O** (Figure 5e), where fiber entanglements formed by objects of homogeneous size were clearly visualized. Once again, a change in solvent nature significantly affects the morphologic nature, and the transition from **[C12–NI-3]­Br/H**_**2**_ **O** to **[C12–NI-3]­Br/Gly** gave rise to the formation of a compact texture in which small spherical objects were observed (Figure 5e,f). Interestingly, the above trend perfectly claims the one observed by fluorescence microscopy that evidenced the transition from spherulitic aggregates to a compact structure in the same systems (cf. Figure 4e,d).

### Biological Investigation

The cytotoxic effect of used organic salts was investigated after 24 h of treatment toward four cancer cell lines of different histological origin, namely HCT-116 (colon cancer), HeLa (cervical cancer), MDA-MB-231 (breast cancer), and SK-MEL 28 (melanoma). In all cases, the MTT assay was performed, using different concentrations of diluted 5 × 10^–4^ M stock solutions of salts, from 100 to 0.097 μM, and the results expressed as IC_50_ values (Figure 6). Globally, IC_50_ values range from 3.59 for **[C14–NI-2]­[Glu]** in HCT-116 cells to 215 μM for **[C12–NI-3]­Br** in HeLa cells. Based on the different nature of the cancer cells, the HeLa cells showed lower cytotoxicity to treatments, except for **[C14–NI-3]­[Glu]**. Similar IC_50_ values were recorded for other cancer cell lines for each treatment, except for **[C14–NI-2]­[Glu],** whose toxicity was higher in HCT-116 cells, indicating that each cancer cell line is more responsive to a specific treatment.

**Figure 6:**
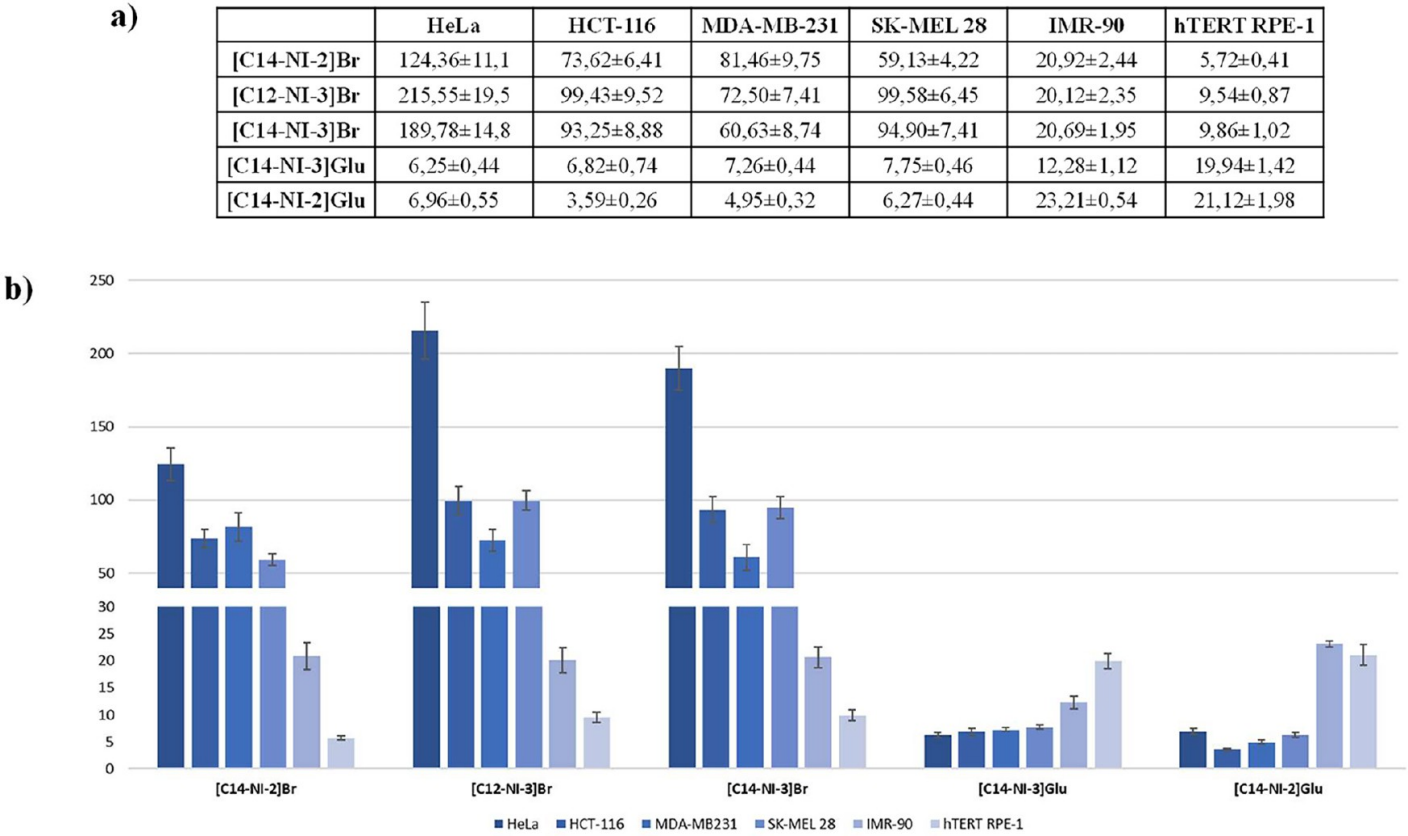
Table (a) and histogram (b) showing the IC_50_ values (concentration that inhibits the 50% of cell proliferation compared to untreated cells) after 24 h of treatment, obtained from the dose–response model, expressed in μM ± SD (standard deviation) of investigated organic salts toward HeLa, HCT-116, MDA-MB-231, and SK-MEL 28 cancer cell lines and IMR-90 and hTERT RPE-1 normal cell lines.

When the different nature of anion is taken into consideration (Figure S9a), it is evident that the gluconate anion exerts a highly toxic effect compared to the bromide one, as account for **[C14–NI-2]­[Glu]** versus **[C14–NI-2]­Br** and **[C14–NI-3]­[Glu**] versus **[C14–NI-3]­Br**, according to the high glucose metabolism in cancer cells, recognized as one of the hallmarks of cancer^63^ It is well-known that accelerated aerobic glycolysis is able to distinguish cancer cells from normal cells and that this distinction has been exploited to detect and image tumors in vivo.^64^

Our results also confirm the role played by the increase in the alkyl chain length on cytotoxic activity (Figure S9b), with a slight increase of cytotoxicity on going from **[C12–NI-3]­Br** to **[C14–NI-3]­Br** salt, according to our previously data, collected by using corresponding imidazolium salts.^27^ However, a different situation can be evidenced by taking into consideration the elongation of the alkyl spacer going from **[C14–NI-2]­Br** to **[C14–NI-3]­Br** or from **[C14–NI-2]­[Glu]** to **[C14–NI-3]­[Glu]** (Figure S9c). Indeed, in these cases, cytotoxicity decreased with the elongation of the alkyl spacer. Probably, according to our previous data, the increase in the spacer length also induces the increase in conformational flexibility, which can make more difficult the interaction with cell membrane, counterbalancing the positive effect due to the increase in hydrophobicity.^65^

Comparison with results previously collected studying cytotoxicity of corresponding imidazolium salts, namely **[C14NIim]­Br** and **[C14NIim]­[Glu]**, toward HeLa and HCT-116 cell lines, evidence a lower cytotoxicity for ammonium salts.^27^ However, differently from the imidazolium salts, which showed comparable IC_50_ values independently from the nature of the cancer cell line, ammonium salts exhibited most significant differences (IC50:4.49 and 3.21 μm for **[C14NIim]­Br** and 124.4 and 73.6 μm for **[C14–NI-2]­Br** toward HeLa and HCT-116, respectively).

Additionally, to assess their selectivity, the salts were tested against two normal human cell lines, namely, IMR-90 (fibroblasts) and hTERT RPE-1 (epithelial cells) (Figure 6). These cell lines were selected because they represent relevant normal counterparts of both epithelial and stromal cell types, thus providing a broader assessment of biocompatibility. Interestingly, bromide-based salts were significantly more toxic in normal cells, with IC_50_ values ranging from 5.72 to 20.92 μM, i.e., lower than those detected in cancer cells, suggesting poor selectivity. Conversely, gluconate-based salts displayed reduced toxicity toward normal cells, with IC_50_ values ranging from 12.28 to 23.21 μM, i.e., higher than those detected in cancer cells. This opposite trend highlights the crucial role of anion in modulating cytotoxicity and supports the hypothesis that gluconate-based salts exert preferential toxicity toward tumor cells, compared to normal cells, likely due to enhanced uptake linked to cancer-specific metabolic pathways, referred as the Warburg effect. Overall, gluconate salts, are the most promising candidates, based on combination of strong anticancer activity and improved biocompatibility with normal human cells.

### Bioimaging

Since the fluorescent salts used in this study could have great potential as both therapeutic and diagnostic agents, fluorescence microscopy was used to investigate their possible application in bioimaging. In all cell lines, blue and green emission was detected only in the cytoplasm and not in the nuclei, after 1 and 6 h of treatment with the IC_50_ concentration (Figure S10). Interestingly, a comparable fluorescence intensity was recorded in both the green and blue channels, according to the fluorescence emission of salts in different solvents. In fact, the intracellular environment is mostly aqueous. Moreover, the obtained results demonstrate a rapid uptake of all organic salts within the cells, but at these concentrations, no significant difference in the emission intensity was observed among different salts.

### Mechanism of Cytoxicity

As stated, the presence of the gluconate anion, unlike the bromide anion, induced a significant increase, at least 10-fold, in the citoxicity and markedly improved the selectivity toward cancer cells compared to normal cells. To gain insights into the possible mechanism underlying this tunability, we first investigated by fluorescence microscopy the uptake of gluconate and bromide salts in SK-MEL 28 cells (Figure 7). After 1 h of treatment at a subtoxic concentration, images acquired under identical excitation time (300 ms) revealed that **[C14–NI-2]­[Glu]** and **[C14–NI-3]­[Glu]**, were taken up more efficiently than the corresponding bromide salts, suggesting that the gluconate anion favors a faster uptake.

**Figure 7:**
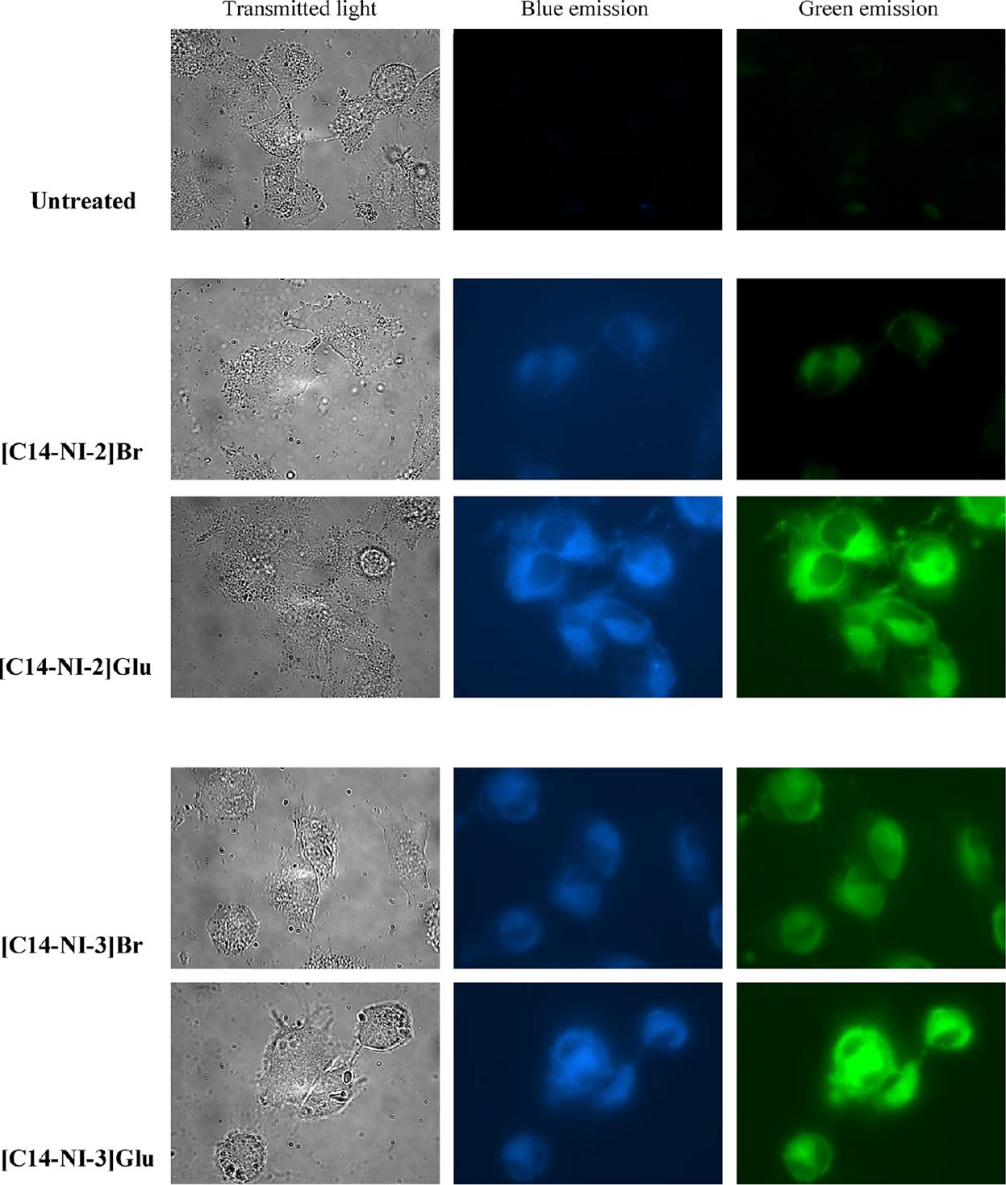
Fluorescence micrographs of SK-MEL28 cells after 1 h of treatment with 5 μM concentration of each salt (excitation range 300 ms). Magnification 630×.

Next, the intracellular localization of **[C14–NI-2]­Glu** and **[C14–NI-3]­Glu** was examined after 24 h of treatment with IC_50_ concentrations. As shown in Figure 8, both salts were localized in the cytoplasm, with bright fluorescence foci, corresponding to organelle localization, such as endocytic/autophagic vacuoles and/or lysosomes, consistent with autophagic/apoptotic cell death. To further investigate the mechanism of cytotoxicity, intracellular ROS production and mitochondrial membrane potential (MMP) were analyzed using DCFH-DA and JC-1 staining, respectively. Since the salts are intrinsically fluorescent, we carefully evaluated the potential interference with assay readouts. Importantly, exposure times used for the dyes (20–50 ms) were considerably shorter than those required to detect salt fluorescence (300 ms), ensuring negligible background interference. Treated cells exhibited a remarkable increase in the level of ROS (Figure 9a), consistent with oxidative stress induction. JC-1 staining revealed a loss of red aggregates together with a more diffuse green fluorescence throughout the cytoplasm compared to untreated cells (Figure 9a), indicative of mitochondrial depolarization. Finally, to discriminate between apoptotic and necrotic cell death, treated cells were subjected to acridine orange/ethidium bromide (AO/EB) staining. As expected (Figure 9a), untreated cells exhibited only green fluorescence due to AO staining of both the cytoplasm and nuclei. In contrast, treated cells displayed a more complex staining pattern, with most cells showing orange fluorescence, membrane blebbing, chromatin condensation, as well as EB-positive nuclei. In some cases, red-stained cells were also detected, suggesting the occurrence of necrotic death.

**Figure 8:**
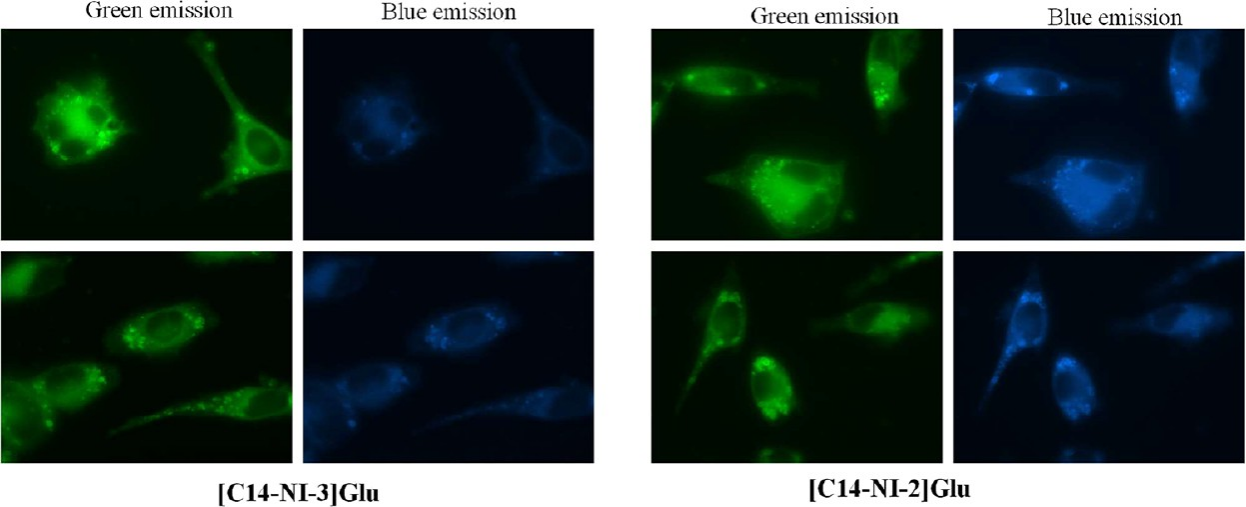
Fluorescence micrographs of SK-MEL28 cells after 24 h of treatment with IC_50_ concentration of **[** **C14–NI-3]­Glu** and **[** **C14–NI-2]­Glu** (excitation range 300 ms). Magnification 630×.

**Figure 9:**
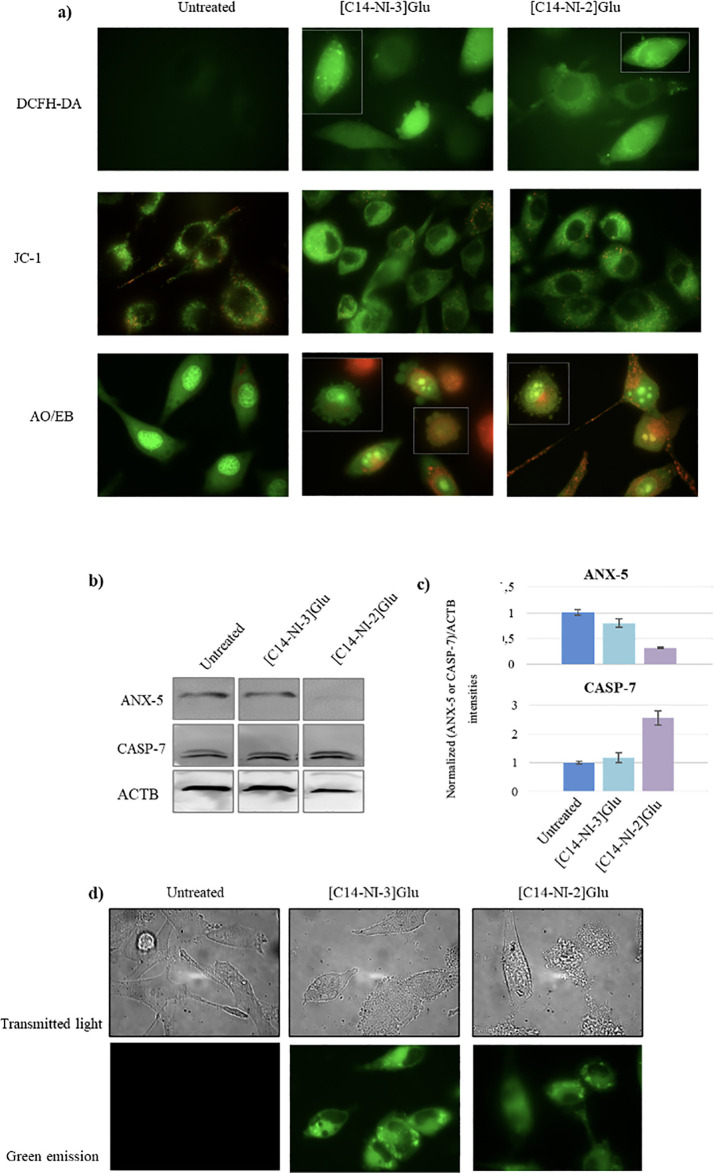
(a) Detection of intracellular ROS production, mitochondrial membrane potential (MMP), and mechanism of cell death, using DCFH-DA, JC-1, and AO/EB staining. For JC-1 and AO/EB staining cells, only the merged images are shown. (b) Western blotting analysis showing the effect of **[** **C14–NI-3]­Glu** and **[** **C14–NI-2]­Glu** treatment (IC_50_ for 24 h) on the expression of ANX-5 and CASP-7 in SK-MEL28 cells. Actin-β was used as a loading control. (c) Histograms showing Western blot quantification, normalized against the Actin-β signal and referred to the untreated control cells. (d) Micrographs of SK-MEL28 cells after 24 h of treatment with IC_50_ concentration of **[** **C14–NI-3]­Glu** and **[C14–NI-2]­Glu** showing morphological alterations in treated cells such as vacuolization and membrane blebbing. Images were also acquired in fluorescence using the excitation range of 300 ms. Magnification 630×.

It is well-known that apoptosis is a fundamental process involved in the maintenance of homeostasis in multicellular organisms. Among the well-documented hallmarks of cells undergoing apoptosis is the redistribution in plasma membrane of phosphatidylserine, a phospholipid almost entirely sequestered in the cytoplasmic leaflet, shedding in the extracellular leaflet during early step of apoptosis.^66^ Annexin V (ANX-5), a protein of yet unknown specific physiologic function, presents a strong affinity for phosphatidylserine and is now widely used for probing cell stimulation or death. Moreover, there is converging evidence for a crucial role of the caspase cascade in the correct execution of a death program. Among the executioner caspases, the subgroup of caspase-3 (CASP-3) and caspase-7 (CASP-7) appears to have a central step.^67^ The above considerations prompted us to assess the expression of markers related to the cell death pathway in **[C14–NI-2]­[Glu]** and **[C14–NI-3]­[Glu]** treated cells. ANX-5 and CASP-7 were probed by Western blot, and their levels were normalized to β-actin (ACTB) level, accordingly (Figure 9b,c). Interestingly, a concomitant downregulation of ANX-5 and upregulation of CASP-7 was detected especially in [**C14–NI-2]­[Glu]** treated cells, suggesting that after 24 h of treatment, cells were in a late apoptosis stage, according to the apparent inhibition of apoptosis detected after ANX-5 protein treatment. Moreover, since the induction of a death program is accompanied by a variety of characteristic changes of the cell morphology, among which shrinkage, plasma membrane blebbing, and nucleus condensation, we also verified morphological changes of treated cells (Figure 9d). Interestingly, membrane blebbing, vacuolization and loss of elongation protrusions were detected in treated cells, consistent with programmed cell death activation. Collectively, these findings support a mechanism of action in which gluconate-based salts enter cancer cells more efficiently, trigger oxidative stress, impair mitochondrial function, and activate apoptosis, with necrosis occurring only in a minority of cells.

Finally, to shed light on the possible action mechanism of the hydrogels, we contacted 400 mg of preformed hydrogel with 25 mL of PBS buffer, to monitor the release of ammonium salt into the buffer solution, by UV–vis spectroscopy, at 37 °C. Initially we set out to conduct this experiment with the [**C14–NI-2]­Glu-**based gel, but this was not possible due to the lack of resistance of the gel to the aqueous phase, which induced its collapse. Consequently, we carried out this release test with the **[C14–NI-2]­Br/H**_**2**_ **O** gel, at 4 wt.%. Although the salts are different since they share similar structural motifs, this experiment can give valuable information for all the gels considered here. The plot of the concentration of salt released into the buffer solution, over time, is reported in Figure S11. Analysis of the plot shows that the hydrogel undergoes significant gelator release after contact with the aqueous buffer. Notably, the concentration released, comprising from (460 ± 20) μM to (1100 ± 50) μM are higher than the IC_50_ value determined for this salt, enabling the cytotoxic effect to be exerted. Hence, the hydrogels act by releasing the ammonium salt, triggering the cytotoxic mechanism above-described. On the grounds of these observation and of literature reports,^27^ we propose that this is the action mechanism for all the hydrogels considered in this work.

## Conclusions

Naphthalimide-based salts, bearing bromide or gluconate anion and differing for the alkyl chain on the charged head or the spacer joining this latter with the aromatic nucleus, were synthesized, and their photophysical behavior was investigated in H_2_O, H_2_O/DMSO mixtures, and glycerol solutions. The salts tested self-assembled, giving rise to the formation of *J*-aggregates, also showing the AIE phenomenon. Fluorescence quantum yields measurements in water, shed light on the impact of hydrophobicity of the salts on their emission behavior, declining in the presence of shorter alkyl chains, and on going from bromide to gluconate anion.

The organic salts formed gel phases in both water and organic solvents, behaving as ambidextrous gelators. The structure of the gelators affected features of soft materials: hydrogels behaved as thixotropic materials and the ability to self-repair was particularly pronounced for those formed in H_2_O/DMSO mixtures and buffer solutions. Interestingly, thixotropic gels exhibited the presence of larger aggregates, which gave rise to the formation of highly emissive materials.

Taking advantage from the significant fluorescence emission and bearing in mind the previously reported antiproliferative activity of imidazolium salts,^68^ the possible application of tested salts as bioimaging and anticancer agents was investigated. All of the synthesized salts showed a significant cytotoxic activity against the tested cancer cell lines, with gluconate derivatives showing the highest activity. The cytotoxicity slight increased from **[C12–NI-3]­Br** to **[C14–NI-3]­Br** salt, confirming our findings about the role played by the increase in the alkyl chain length on cytotoxic activity. In general, ammonium salts proved to be less cytotoxic than the corresponding imidazolium salts, but IC_50_ values exhibited higher differences depending on the nature of the cancer cell line.

New insights were gained regarding the effect of the alkyl spacer length on the biological activity. In fact, salts with shorter alkyl spacer, like **[C14–NI-2]­Br** were more toxic than the longer one **[C14–NI-3]­Br**. However, all of the bromide-based salts lacked selectivity toward cancer cells, showing comparable or higher toxicity in normal cells. In contrast, the replacement of bromide with gluconate enhanced the selectivity for cancer cells. This effect could be due to the more efficient internalization of gluconate-based salts in metabolically active tumor cells, which is consistent with the Warburg effect. Conversely, in normal cells that do not exhibit the Warburg effect, gluconate salts are internalized less efficiently, leading to a lower toxicity. Once inside the cells, they localize predominantly into the cytoplasm, where they induce oxidative stress, mitochondrial depolarization, and the activation of apoptotic pathways, as the dominant mode of cell death, although a small fraction of necrotic cells was observed. Overall, our findings provide mechanistic insights into the tunability of cytotoxicity based on the anion and cation structure and conformation, highlighting gluconate-based salts as promising candidates for the development of new bioimaging and anticancer agents. Further investigation is needed to clarify the pathways specifically involved in cell death induction. Finally, based on release experiments, we propose that the hydrogels act by releasing the ammonium salt in the culture medium enabling then the cytotoxic activity discussed above. Consequently, the ammonium salts presented here show great potential for application both in solution and as active ingredients of semi solid materials.

## Supplementary Material



## Notes

Experimental procedures for the synthesis of gelators, UV–vis and fluorescence spectra for organic salts in in different solvents, UV–vis and fluorescence spectra in H_2_O and in the presence of 9,10-phenantroline in ethanol, plots of strain and frequency sweep measurements on gel phases, thixotropy test performed on hydrogels, plots of opacity and I_RLS_ value as a function of the time, emission spectra for hot solutions and corresponding gel phases, histograms showing the effect of structural changes of investigated organic salts on cytotoxicity toward HeLa, HCT-116, MDA-MB-231 and SK-MEL 28 cancer cell lines, fluorescence micrographs of MDA-MB-231 cells after 1 and 6 h of treatment with the IC_50_ concentration of each salt, plot of gelator release from the gel, ^1^H and ^13^C NMR spectra, table of position of main absorption and emission band for organic salts as a function of solvent nature, table of emission quantum yield for organic salts in water solution, table of gelation tests, results of thixotropy and sonotropy tests performed on gel phases, table for RLS intensity for gel phases, and table of λ_max_ (nm) and emission intensity (I) of hot solutions and corresponding gel phases (PDF)
