# Supplementary material for: Unravelling the Properties of Fluorescent Ammonium Salts to Obtain Thixotropic Hydrogels with Antitumoral Activity
Source: ACS Omega. 2025 Dec 23;11(1):663–80. doi: 10.1021/acsomega.5c06795 (PMC12809299; doi:10.1021/acsomega.5c06795)
Supplement: Supplementary file 1 [file ao5c06795_si_001.pdf]

Supporting Information for

Unravelling the Properties of Fluorescent Ammonium Salts to Obtain Thixotropic Hydrogels with Antitumoral Activity

Floriana Billeci[a,c,e], Miriam Buttacavoli[b,d,e], Emanuela Peri [b], Salvatore Marullo [a], Patrizia Cancemi[a], Francesca D'Anna*[a]*

[a]*Università degli Studi di Palermo, Dipartimento STEBICEF, Viale delle Scienze, Ed. 17 "S. Cannizzaro", 90128 Palermo (Italy).*

[b]*Università degli Studi di Palermo, Dipartimento STEBICEF, Viale delle Scienze, Ed. 16, 90128 Palermo (Italy).*

[c]*Until March 2022.* [d]*Until January 2024* [e]*The authors share the same role*

Experimental Procedures for the synthesis of gelators and characterization	**S3**
**Figure S1.** UV-vis (0.0001 M; left) and emission spectra (0.00001 M; right) of organic salts in different solvents.	**S5**
**Figure S2.** Picture of gel phases formed: **a) [C14-NI-2]Br/H_2O** and **b) [C14-NI-2]Br/Gly**.	**S6**
**Figure S3.** UV-vis (0.0001 M; left) and emission spectra (0.00001 M; right) of organic salts in H_2O and 9,10-phenantroline as standard in Ethanol solution.	**S7**
**Figure S4.** Plot of frequency (left) and strain sweep (right) of different hydrogels at 4%wt.	**S8**
**Figure S5.** Thixotropy test performed on hydrogels at 4% wt.	**S11**
**Figure S6.** Plot of opacity, measured at l = 568 nm, as a function of time for different hydrogels at 45 wt.	**S12**
**Figure S7.** Plot of I_{RLS} as a function of time for different hydrogels at 4% wt.	**S14**
**Figure S8.** Emission spectra of hot solutions and gel phases 4% wt.	**S16**
**Figure S9**. Histograms showing the effect of structural changes of investigated organic salts on cytotoxicity toward Hela, HCT-116, MDA-MB231 and SK-MEL 28 cancer cell lines. IC_{50} values were calculated after 24 h of treatment from the dose-response model and expressed in µM ±SD (standard deviation).	**S18**
**Figure S10.** Fluorescence micrographs of MDA-MB231 cells after 1 h and 6 h of treatment with the IC_{50} concentration of each salt (excitation range 300 ms). Magnification 630×.	**S20**
**Figure S11.** Plot of gelator release as a function of time for the [**C14-NI-2]Br** hydrogel in contact with 25 mL of PBS.	**S20**
**Figure S12.** ^1H and ^{13}C NMR spectra	**S21**
**Table S1.** Position of main absorption and emission band for organic salts as a function of the solvent nature	**S26**
**Table S2.** Emission quantum yield for organic salts in water solution, measured using 9,10-phenantroline in ethanol as standard.	**S26**
**Table S2.** Gelation tests for **[C14-NI-2]Br** and **[C14-NI-3]Br**	**S26**
**Table S3.** Gelation tests for **[C12-NI-3]Br** and **[C14-NI-3][Glu]**	**S26**
**Table S4.** I_{RLS}, opacity values and gelation times for different gel phases at 4 wt %.	**S26**
**Table S5.** Thixotropy and sonotropy tests performed on gel phases at 4% (w/w).	**S27**
**Table S6.** I_{RLS}, opacity values and gelation times for different gel phases at 4 wt %.	**S27**
**Table S7.** λ_{max} (nm) and emission intensity (I) of hot solutions and corresponding gel phases at 4% wt.	**S27**

Synthesis of the gelators

General procedure for the synthesis of the neutral precursors

The neutral precursors were prepared by modification of previously reported procedures (10.1016/j.dyepig.2010.01.005, 10.1021/acsami.0c17149).

In a two-necked round-bottom flask, 1 g (4.39 mmol) of 4-chloro-1,8-naphthalic anhydride was dissolved in toluene at 110 °C for 30 min. To this stirred solution, 1.2 eq. of the suitable diamine was added dropwise. The mixture was kept at 110 °C for 5h, under stirring. Subsequently, the mixture was allowed to cool down at room temperature and the solvent was removed by evaporation at reduced pressure, obtaining a yellow solid. The crude product was purified by flash chromatography on silica (DCM/MeOH, 30/1), obtaining the products as yellow solids.

4-Chloro-N-[3-(dimethylamino)ethyl]-1,8-naphthalic anhydride

Yellow solid. Yield: 88%. ^{1}H NMR (300 MHz, $CDCl_3$) δ (ppm): 8.67 (2H, d, J = 9.0 Hz), 8.60 (1H, d, J = 9.0 Hz), 8.51 (1H, d, J =9.0 Hz), 7.85 (2H, m), 4.34 (2H, t, J = 9.0 Hz), 2.67 (2H, t, J = 9.0 Hz), 2.37 (6H, s). ^{13}C NMR (300 MHz, $CDCl_3$) δ (ppm): 163.8, 164.6, 139.0, 132.1, 131.2, , 130.6, 129.3, 129.1, 127.8, 127.4, 123.1, 121.6, 57.0, 45.8, 38.3.

4-Chloro-N-[3-(dimethylamino)propyl]-1,8-naphthalic anhydride

Yellow solid. Yield: 80%. ^{1}H NMR (300 MHz, $CDCl_3$) δ (ppm): 8.64 (2H, d, J = 6.0 Hz), 8.57 (1H, d, J = 6.0 Hz), 8.48 (1H, d, J = 6.0 Hz), 7.85 (1H, d, J = 9.0 Hz), 7.81 (1H, d, J = 9.0 Hz), 4.23 (2H, t, J = 6.0 Hz), 2.44 (2H, t, J = 6.0 Hz), 2.26 (6H, s), 2.44 (2H, quin, J = 6.0 Hz). ^{13}C NMR (300 MHz, $CDCl_3$) δ (ppm): 163.7, 164.3, 138.9, 133.8, 131.9, 131.0, 130.5, 129.3, 129.0, 127.3, 123.1, 121.6, 57.4, 45.3, 39.1, 26.1.

General Procedure for the Synthesis of Naphthalimide Bromide Salts [C_nNIi_m][Br].

The precursor (1g) was dissolved in 30 mL of acetonitrile, 2 eq. of the suitable alkyl bromide compound (n-dodecyl, or n-tetradecyl) were added to the solution and the resulting mixture was refluxed for 72 h. Subsequently, the reaction mixture was allowed to cool down and the solvent was removed by evaporation. The residue obtained was first washed with portions (4 × 10 mL) of ethyl acetate, and then dissolved in dichlorometane and washed with deionized water in a separating funnel (2 × 20 mL). The combined extracts were dried with Na_2SO_4 and evaporation of the solvent yielded the products as pale-yellow solids.

[C14-NI2][Br]

Pale yellow solid. Yield: 96%. ^{1}H NMR (300 MHz, $CDCl_3$) δ (ppm): 8.65 (2H, t, J = 6.0 Hz), 8.50 (1H, d, J = 6.0 Hz), 7.90 (1H, d, J = 9.0 Hz), 7.84 (1H, d, J = 9.0 Hz), 4.67 (2H, t, J = 6.0 Hz), 3.91 (2H, t, J = 6.0 Hz), 3.76 (2H, m), 3.66 (6H, s), 1.83 (4H, m), 1.26 (20H, m), 0.88 (3H, t, J = 6.0 Hz), ^{13}C NMR (300 MHz, $CDCl_3$)

δ (ppm): 163.6, 163.3, 140.2, 132.8, 131.9, 131.6, 129.3, 129.4, 129.0, 128.2, 127.7, 122.1, 120.5, 64.5, 60.0, 52.0, 31.9, 29.7, 29.6 (2C), 29.4 (2C), 29.3, 29.2, 14.1.

[C14-NI3][Br]

Pale yellow solid. Yield: 90%. ^{1}H NMR (300 MHz, $CDCl_3$) δ (ppm): 8.52 (1H, d, *J* = 6.0 Hz), 8.48 (1H, d, *J* = 6.0 Hz), 8.35 (1H, d, *J* = 9.0 Hz), 7.77 (1H, d, *J* = 6.0 Hz), 7.72 (1H, d, *J* = 9.0 Hz), 4.26 (2H, t, *J* = 6.0 Hz), 3.77 (2H, t, *J* = 6.0 Hz), 3.58 (2H, m), 3.46 (6H, s), 2.27 (3H, m), 1.71 (2H, m), 1.25 (20H, m), 0.87 (3H, t, *J* = 6.0 Hz). ^{13}C NMR (300 MHz, $CDCl_3$) δ (ppm): 163.8, 163.5, 139.6, 132.3, 131.4, 131.0, 129.2, 128.9, 127.9, 127.4, 122.4, 120.9, 64.2, 61.6, 51.5, 45.2, 52.0, 37.5, 32.6, 29.7, 29.6 (2C), 29.5 (2C), 29.3, 29.2, 28.9, 22.7, 21.9, 14.1.

[C12-NI3][Br]

Pale yellow solid. Yield: 95%. ^{1}H NMR (300 MHz, $CDCl_3$) δ (ppm): 8.33 (1H, d, *J* = 6.0 Hz), 8.24 (1H, d, *J* = 6.0 Hz), 8.15 (1H, d, *J* = 9.0 Hz), 7.61 (1H, d, *J* = 6.0 Hz), 7.55 (1H, d, *J* = 9.0 Hz), 4.60 (2H, m), 4.17 (2H, m), 3.82 (2H, m), 3.54 (1H, m), 3.30 (6H, s), 2.16 (1H, m), 1.69 (1H, m); 1.23 (19H, m), 0.89 (3H, t, *J* = 6.0 Hz). ^{13}C NMR (300 MHz, $CDCl_3$) δ (ppm): 163.4, 163.1, 139.1, 132.0, 131.0, 130.5, 128.6, 127.9, 127.3, 122.0, 122.6, 51.8, 45.2, 31.9, 29.7, 29.6, 29.5 (2C), 29.4 (2C), 22.1, 14.1.

For the synthesis of the gluconate-based salt, an anion-exchange procedure was carried out with resin Amberlite IRA-400 (Chloride form); after that, a previous cationic exchange was needed to convert potassium gluconate in gluconic acid using the acid resin, Amberlite IR-120, as previously reported (rif: 10.1016/j.jcis.2019.04.034).

[C14-NI2][Glu]

Orange solid. Yield: 92%. ^{1}H NMR (300 MHz, $CDCl_3$) δ (ppm): 8.33 (1H, d, *J* = 6.0 Hz), 8.38 (1H, d, *J* = 9.0 Hz), 8.32 (1H, d, *J* = 9.0 Hz), 7.69 (2H, m), 4.53 (6H, m), 3.70 (3H, m), 3.40 (6H, s), 1.73 (2H, m), 1.25 (29H, m), 0.86 (3H, m). ^{13}C NMR (300 MHz, $CDCl_3$) δ (ppm): 177.63, 176.24, 163.53, 139.67, 132.43, 131.61, 131.02, 128.83, 127.95, 121.86, 120.31, 64.00, 63.17, 59.05, 52.29, 26.69, 29.54, 29.45, 29.39, 29.35, 29.25, 25.75, 22.69, 14.13.

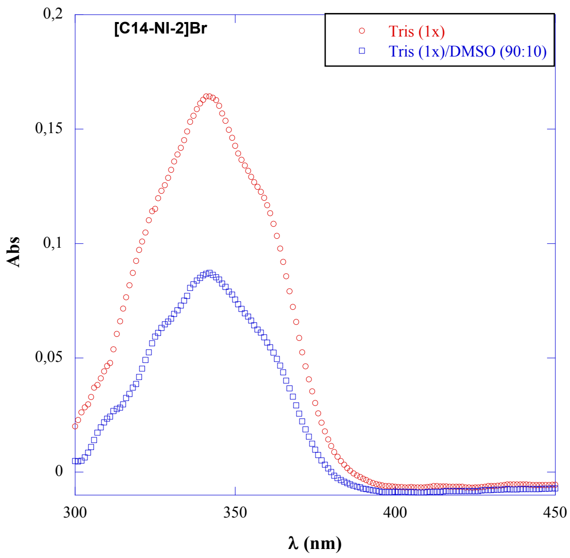
[C14-NI-2]Br
Tris (1x)
Tris (1x)/DMSO (90:10)
Abs
λ (nm)

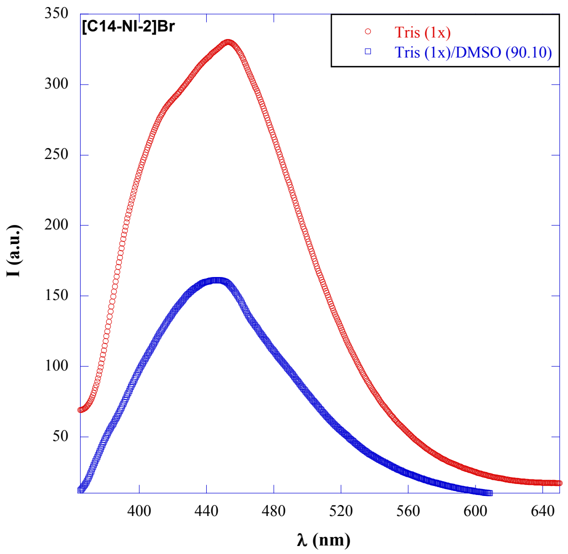
[C14-NI-2]Br
Tris (1x)
Tris (1x)/DMSO (90.10)
I (a.u.)
λ (nm)

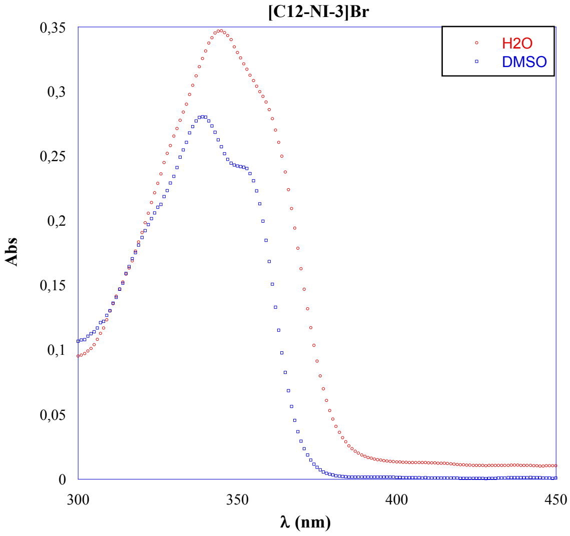
[C12-NI-3]Br
H2O
DMSO
Abs
λ (nm)

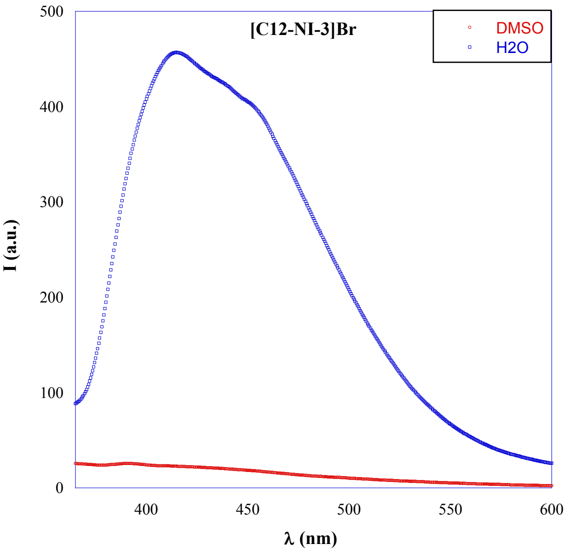
[C12-NI-3]Br
DMSO
H2O
I (a.u.)
λ (nm)

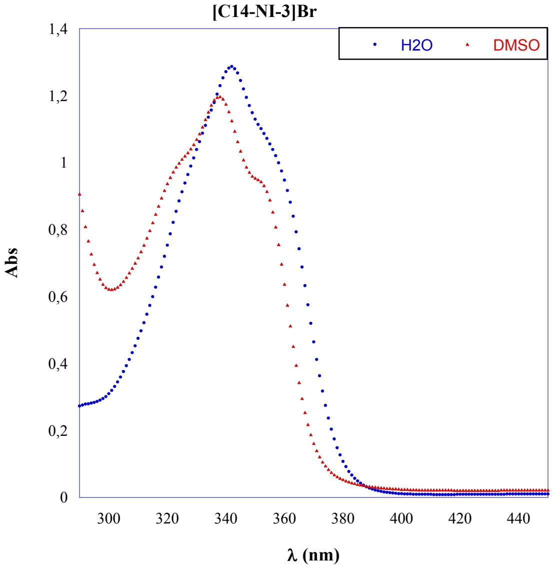
[C14-NI-3]Br
H2O
DMSO
Abs
λ (nm)

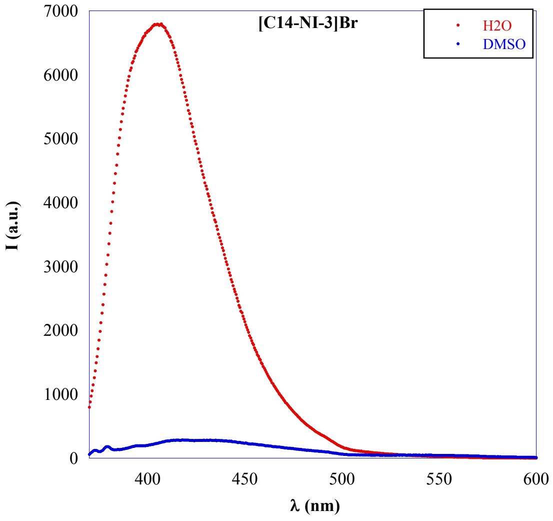
[C14-NI-3]Br
H2O
DMSO
I (a.u.)
λ (nm)

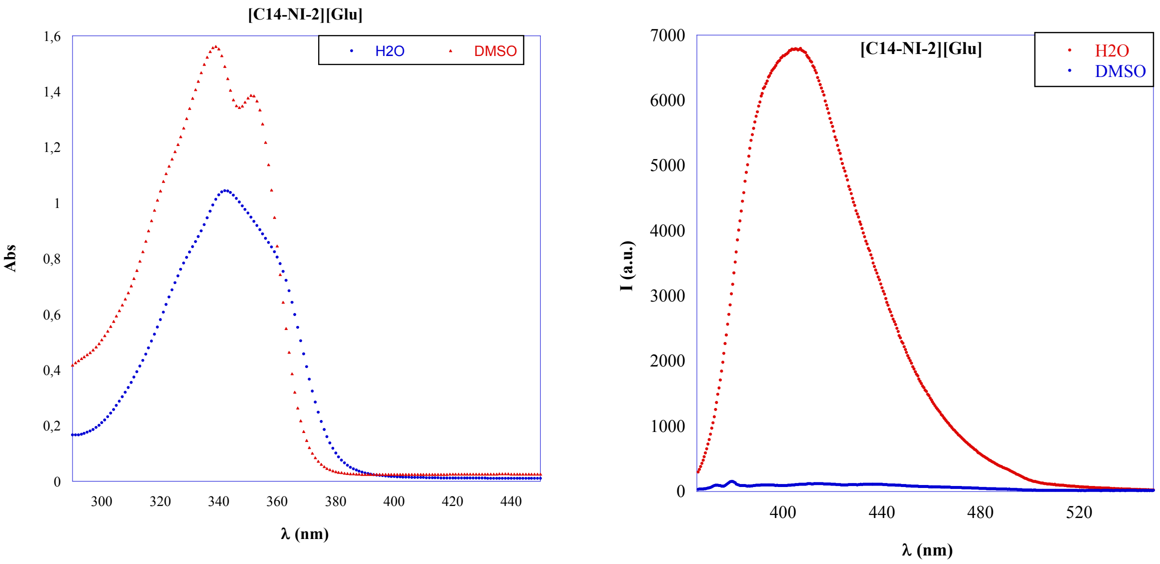

Figure S1. UV-vis (0.0001 M; left) and emission spectra (0.00001 M; right) of organic salts in different solvents.

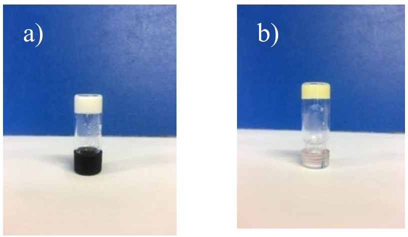

Figure S2. Picture of gel phases formed: **a) [C14-NI-2]Br/H_2O** and **b) [C14-NI-2]Br/Gly**.

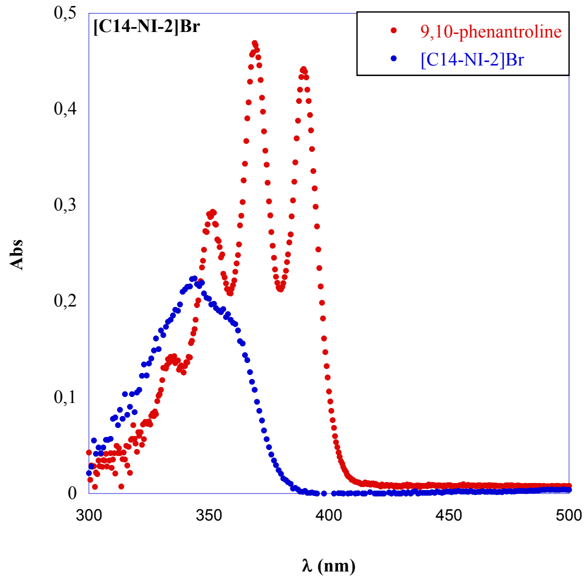
[C14-NI-2]Br
9,10-phenantroline
[C14-NI-2]Br
Abs
λ (nm)

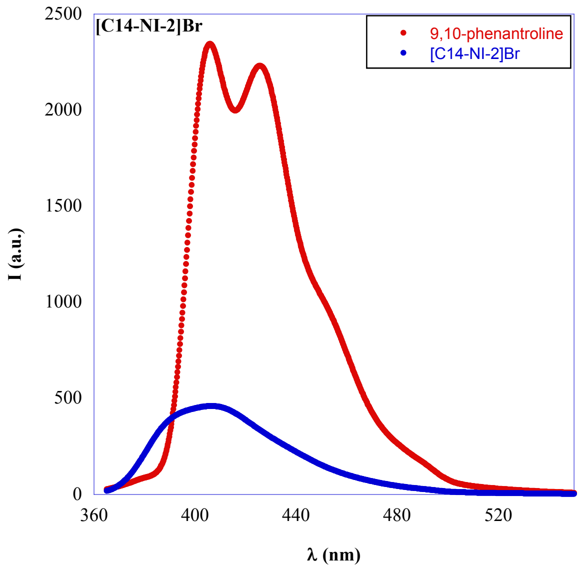
[C14-NI-2]Br
9,10-phenantroline
[C14-NI-2]Br
I (a.u.)
λ (nm)

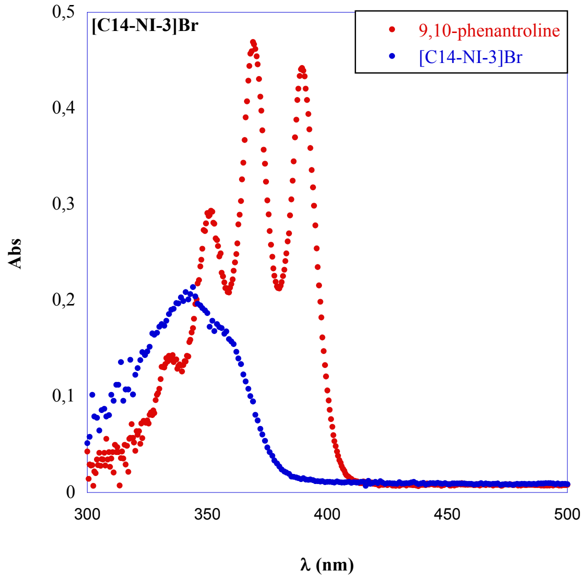
[C14-NI-3]Br
9,10-phenantroline
[C14-NI-3]Br
Abs
λ (nm)

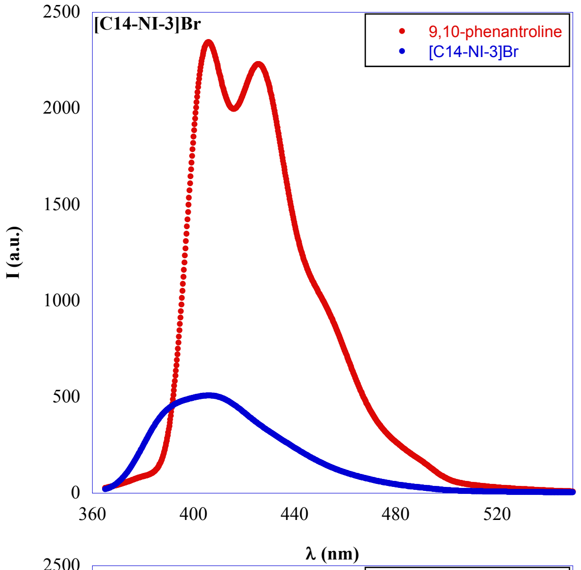
[C14-NI-3]Br
9,10-phenantroline
[C14-NI-3]Br
I (a.u.)
λ (nm)

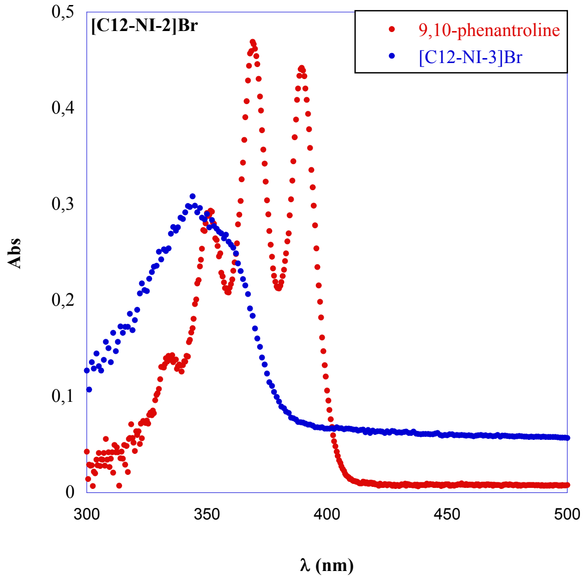
[C12-NI-2]Br
9,10-phenantroline
[C12-NI-3]Br
Abs
λ (nm)

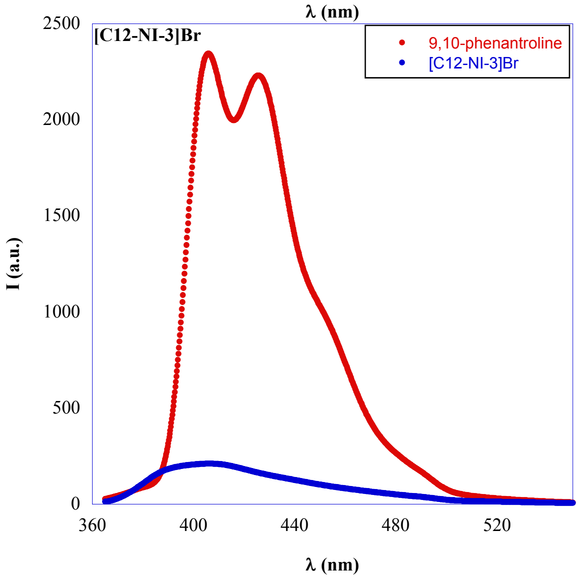
[C12-NI-3]Br
9,10-phenantroline
[C12-NI-3]Br
I (a.u.)
λ (nm)

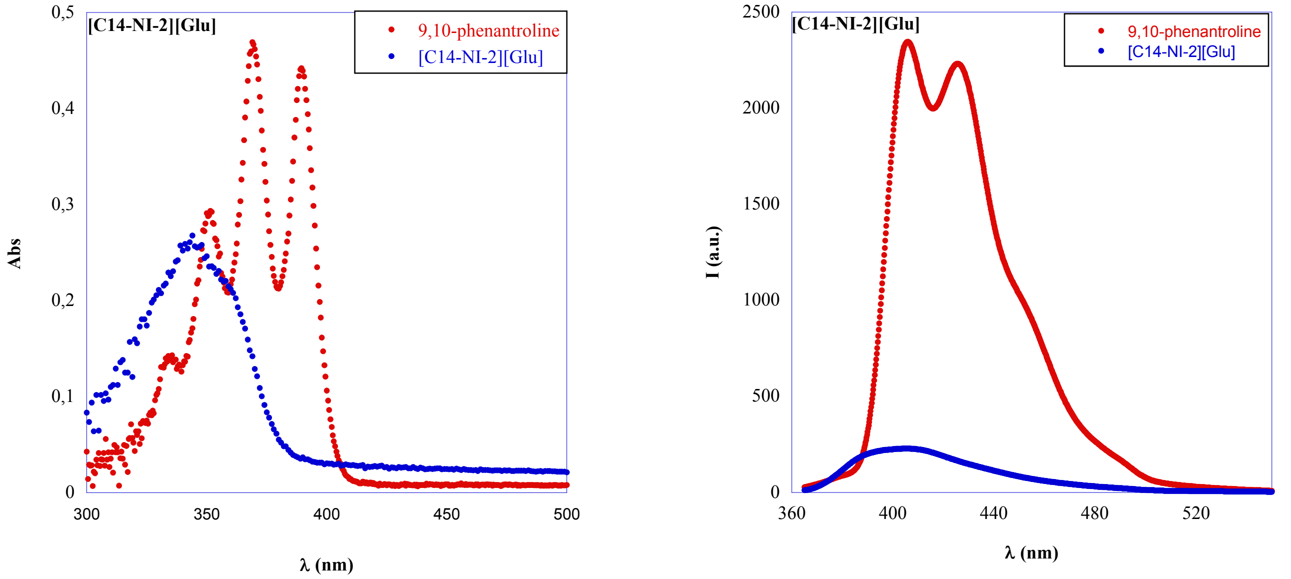

Figure S3. UV-vis (0.0001 M; left) and emission spectra (0.00001 M; right) of organic salts in H_2O and 9,10-phenantroline as standard in ethanol solution.

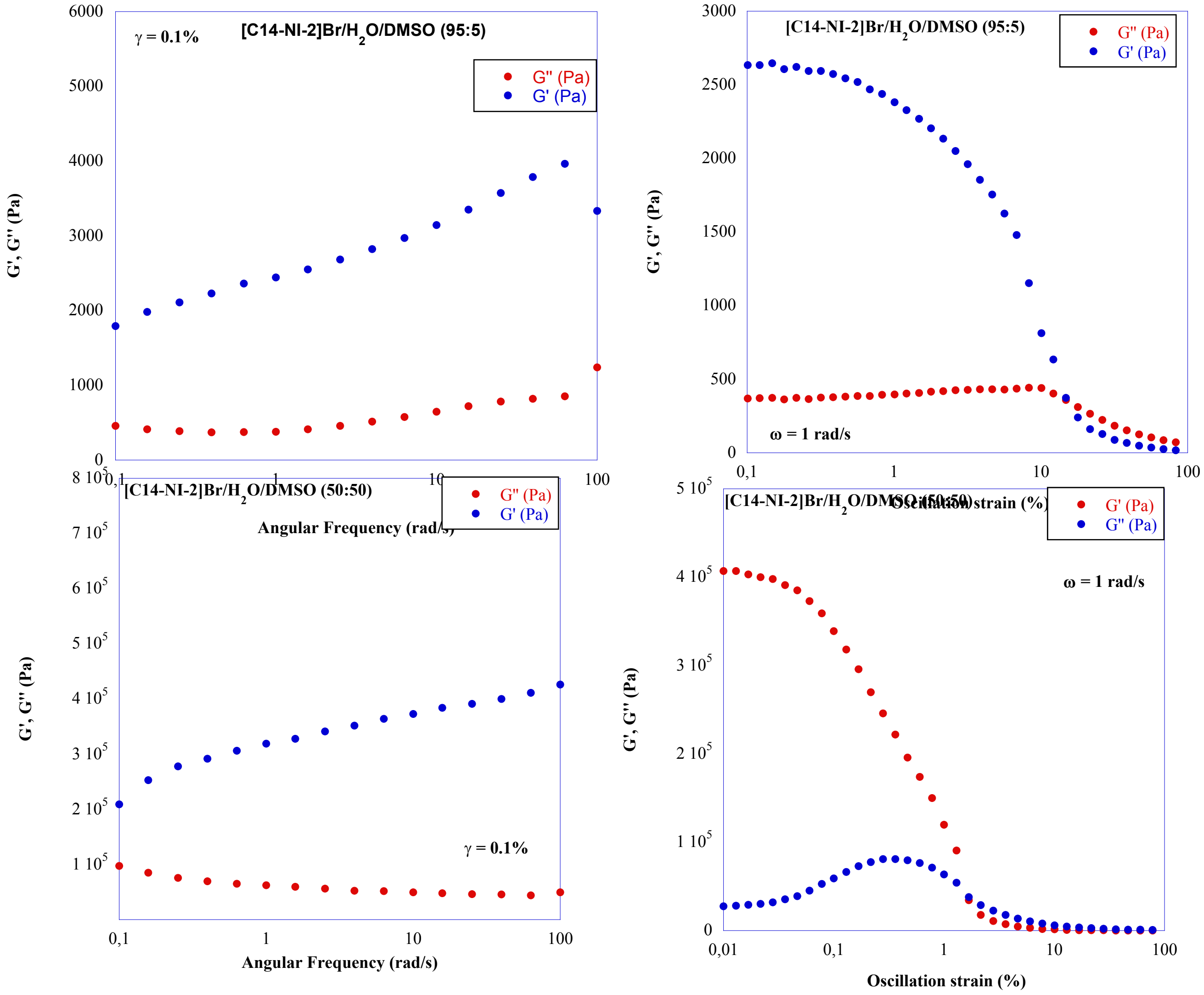

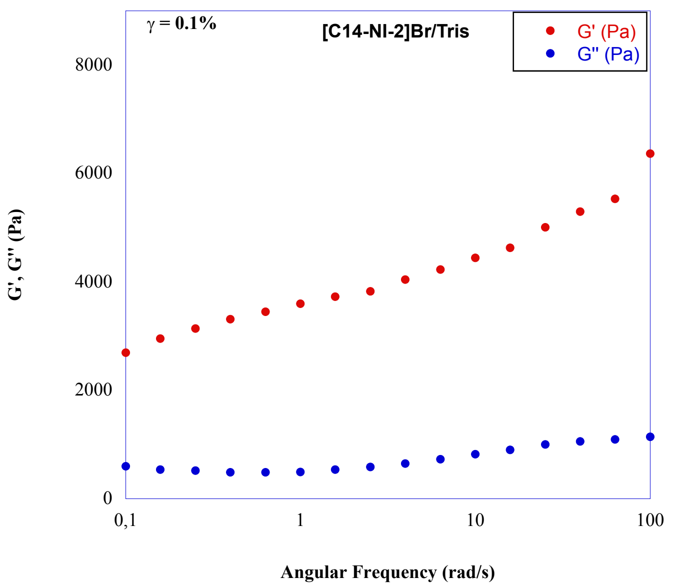
γ = 0.1%
[C14-NI-2]Br/Tris
G' (Pa)
G" (Pa)
G', G" (Pa)
0
2000
4000
6000
8000
0,1
1
10
100
Angular Frequency (rad/s)

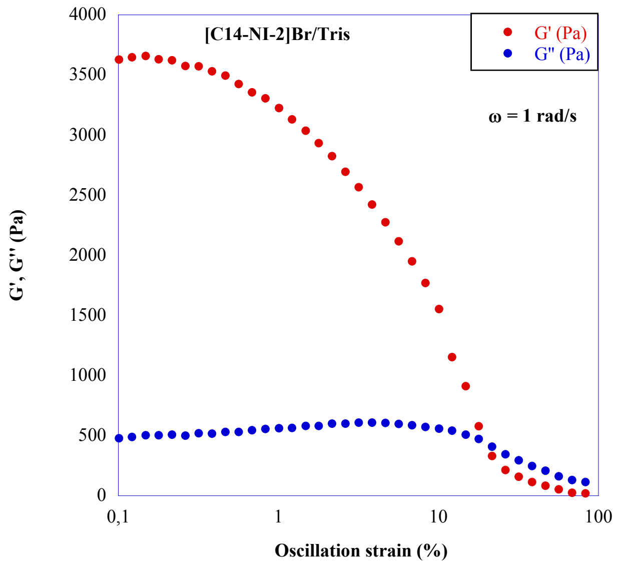
[C14-NI-2]Br/Tris
G' (Pa)
G" (Pa)
ω = 1 rad/s
G', G" (Pa)
0
500
1000
1500
2000
2500
3000
3500
4000
0,1
1
10
100
Oscillation strain (%)

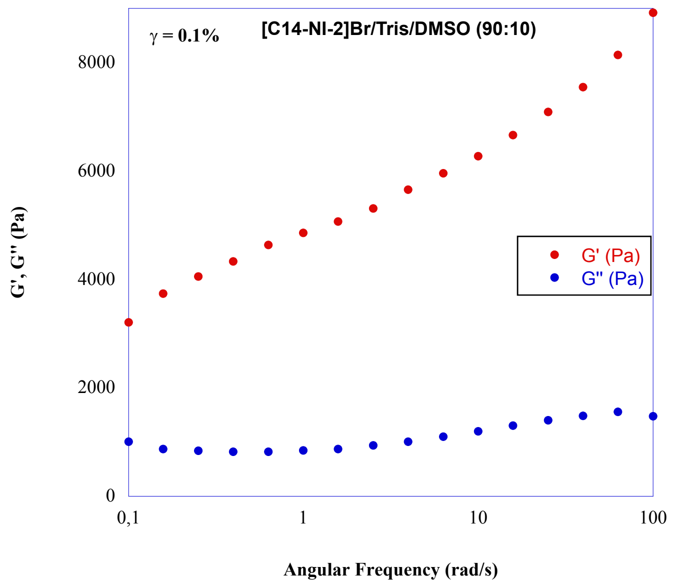
γ = 0.1%
[C14-NI-2]Br/Tris/DMSO (90:10)
G' (Pa)
G" (Pa)
G', G" (Pa)
0
2000
4000
6000
8000
0,1
1
10
100
Angular Frequency (rad/s)

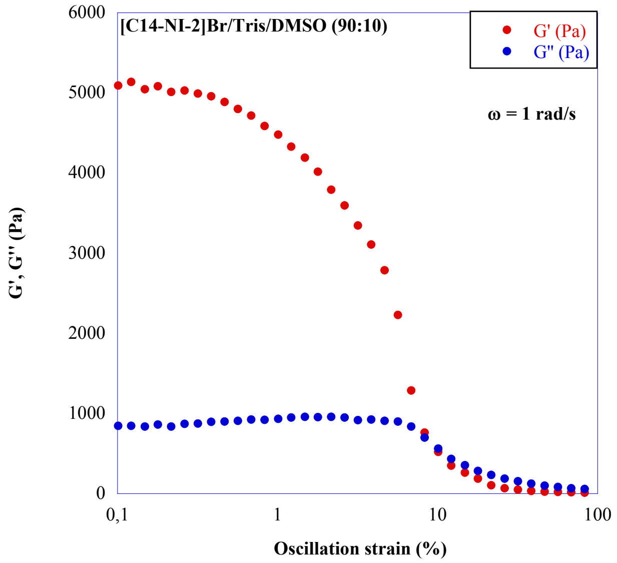
[C14-NI-2]Br/Tris/DMSO (90:10)
G' (Pa)
G" (Pa)
ω = 1 rad/s
G', G" (Pa)
0
1000
2000
3000
4000
5000
6000
0,1
1
10
100
Oscillation strain (%)

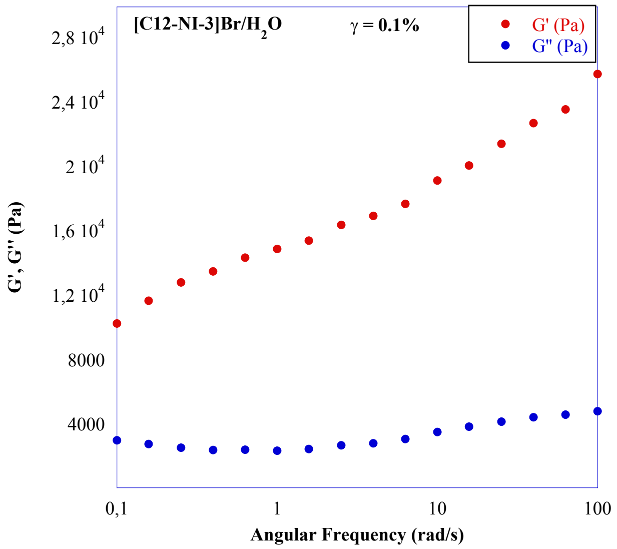
[C12-NI-3]Br/H2O
γ = 0.1%
G' (Pa)
G" (Pa)
G', G" (Pa)
4000
8000
1,2 10^4
1,6 10^4
2 10^4
2,4 10^4
2,8 10^4
0,1
1
10
100
Angular Frequency (rad/s)

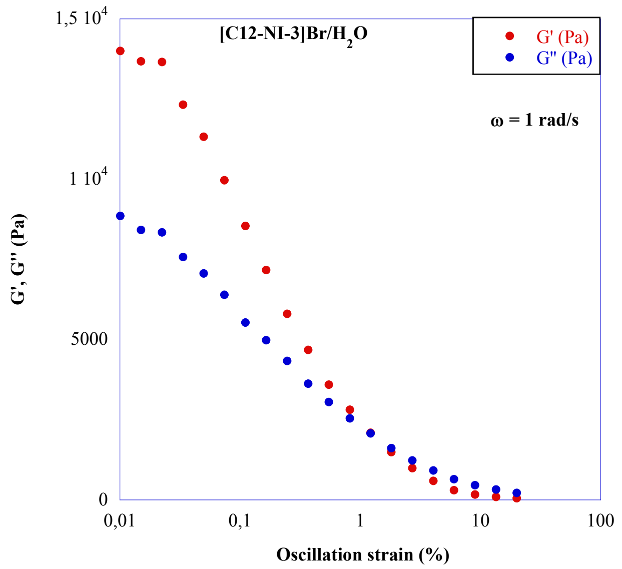
[C12-NI-3]Br/H2O
G' (Pa)
G" (Pa)
ω = 1 rad/s
G', G" (Pa)
0
5000
1 10^4
1,5 10^4
0,01
0,1
1
10
100
Oscillation strain (%)

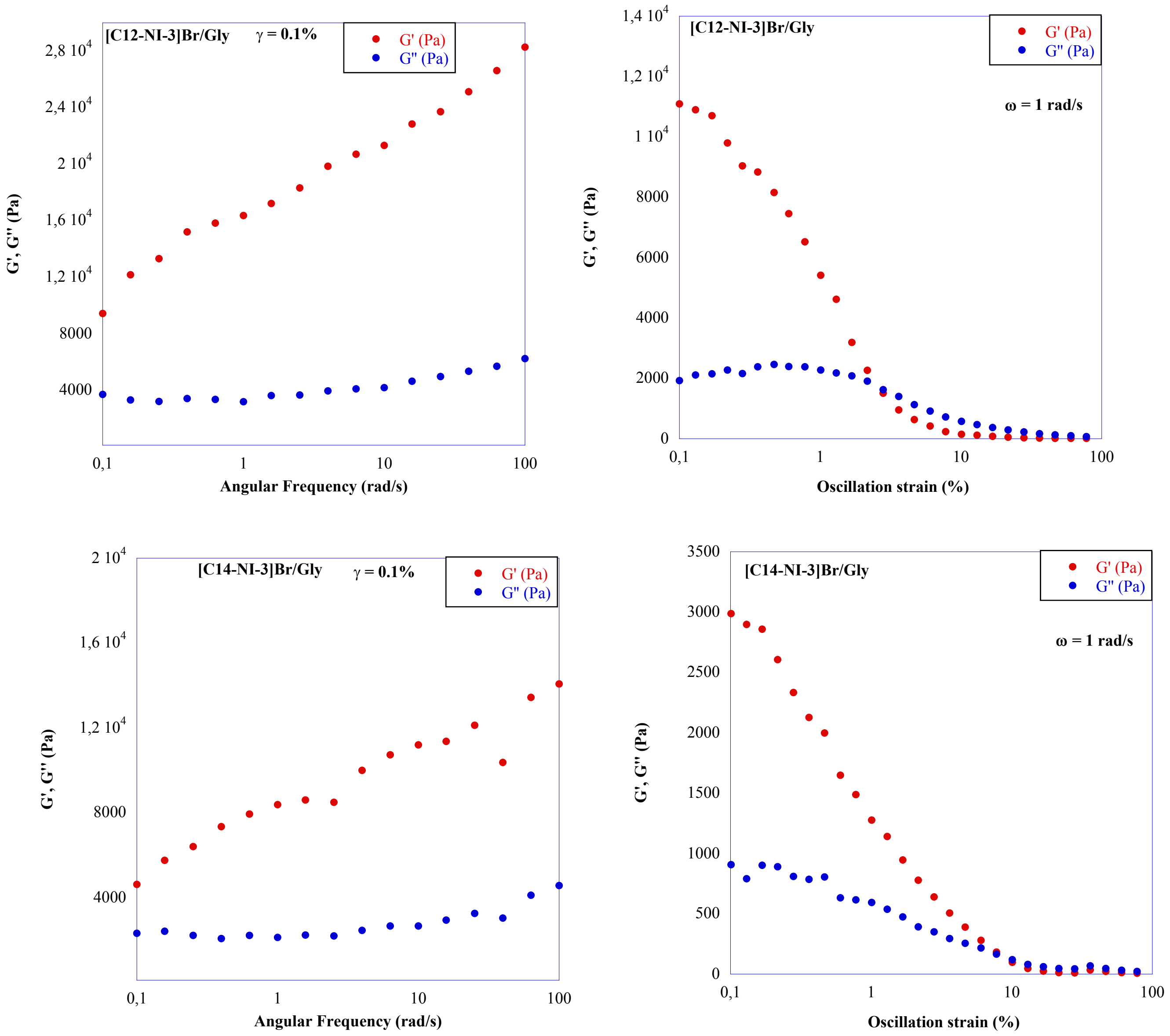

Figure S4. Plot of frequency (left) and strain sweep (right) of different hydrogels at 4%wt.

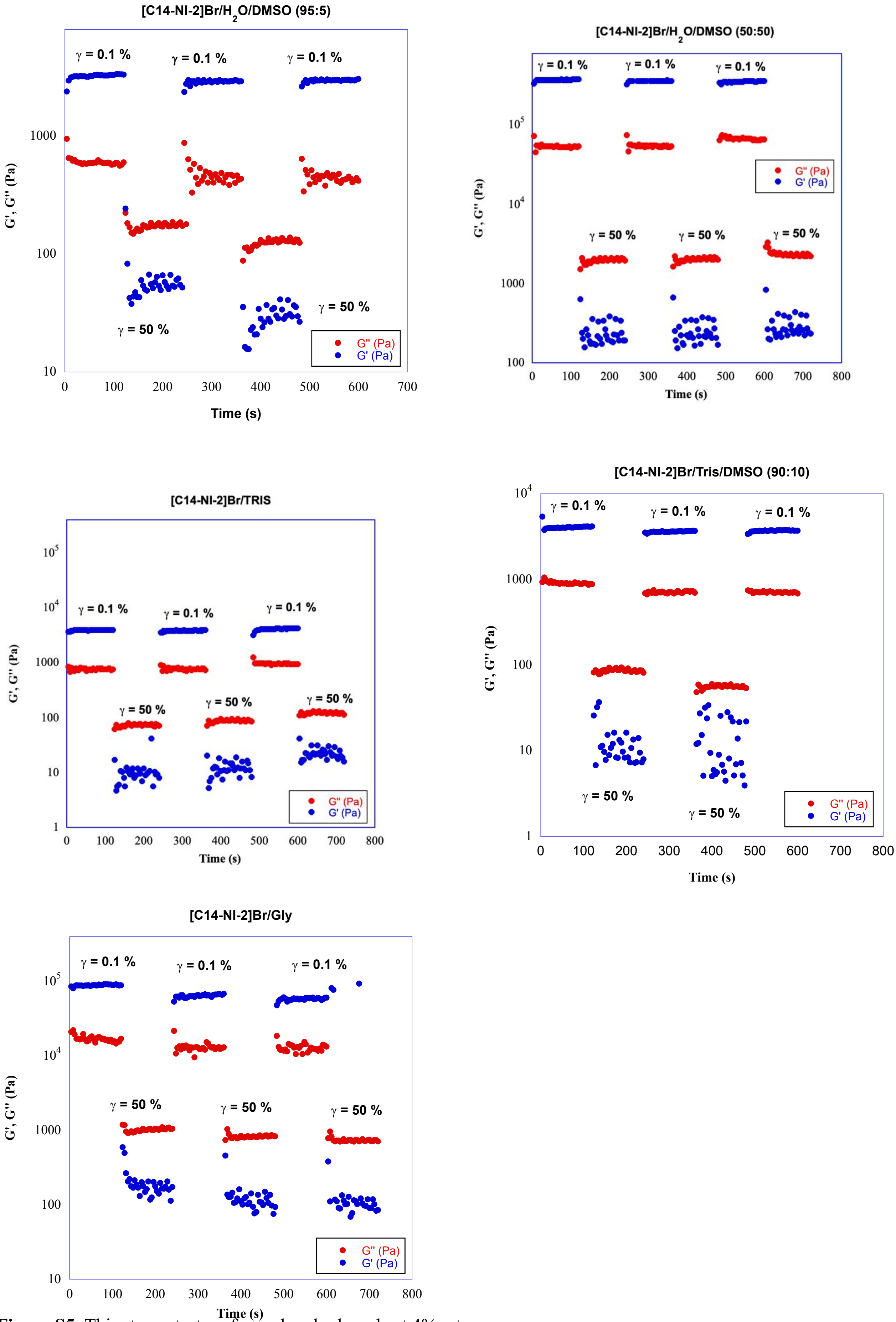

Figure S5. Thixotropy test performed on hydrogels at 4% wt.

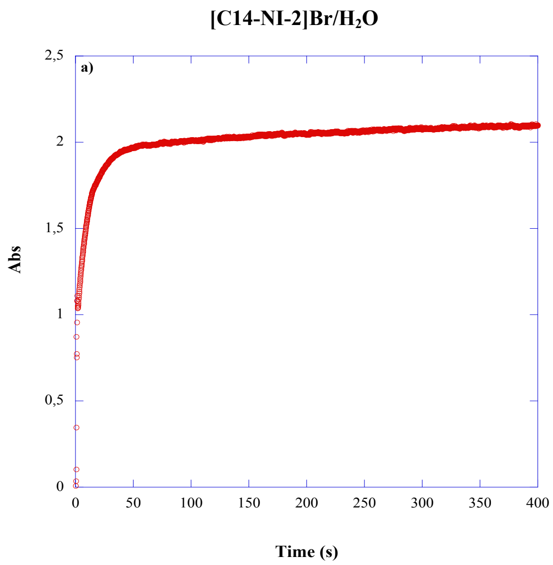
[C14-NI-2]Br/H_2O
a)
Abs
Time (s)
2,5
2
1,5
1
0,5
0
0
50
100
150
200
250
300
350
400

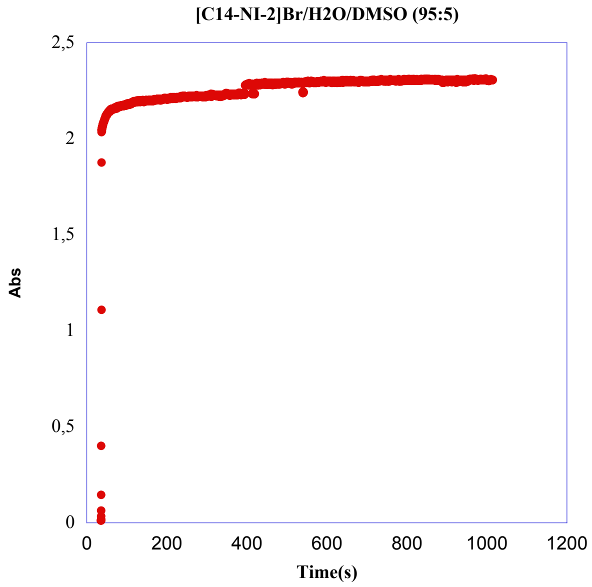
[C14-NI-2]Br/H2O/DMSO (95:5)
Abs
Time(s)
2,5
2
1,5
1
0,5
0
0
200
400
600
800
1000
1200

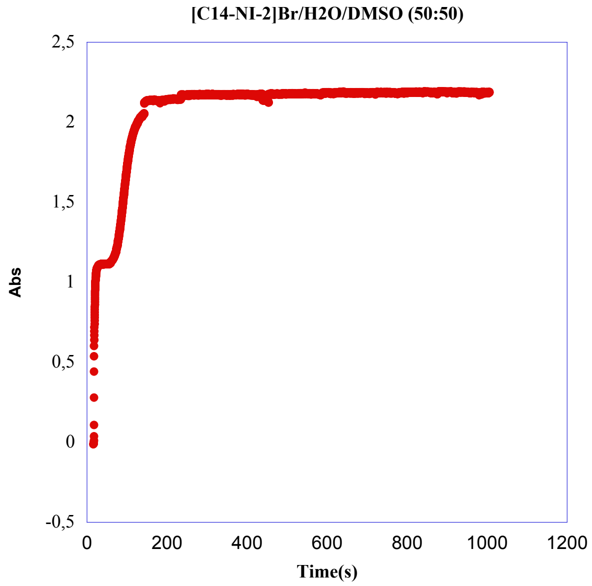
[C14-NI-2]Br/H2O/DMSO (50:50)
Abs
Time(s)
2,5
2
1,5
1
0,5
0
-0,5
0
200
400
600
800
1000
1200

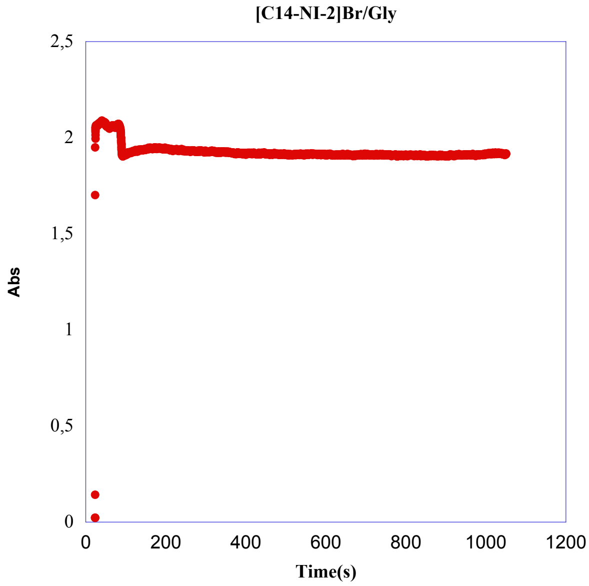
[C14-NI-2]Br/Gly
Abs
Time(s)
2,5
2
1,5
1
0,5
0
0
200
400
600
800
1000
1200

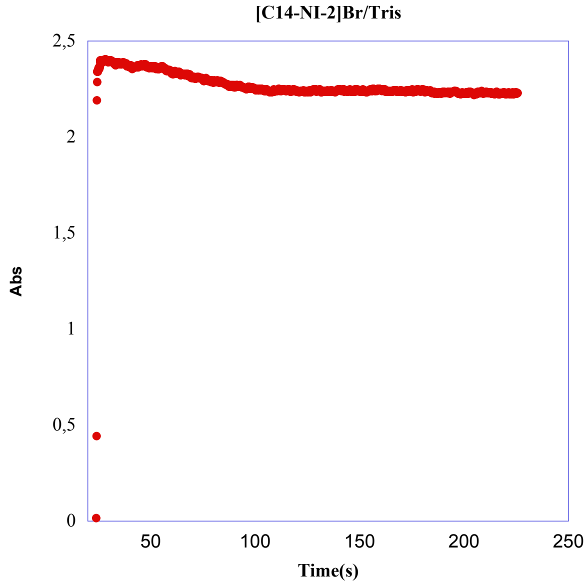
[C14-NI-2]Br/Tris
Abs
Time(s)
2,5
2
1,5
1
0,5
0
50
100
150
200
250

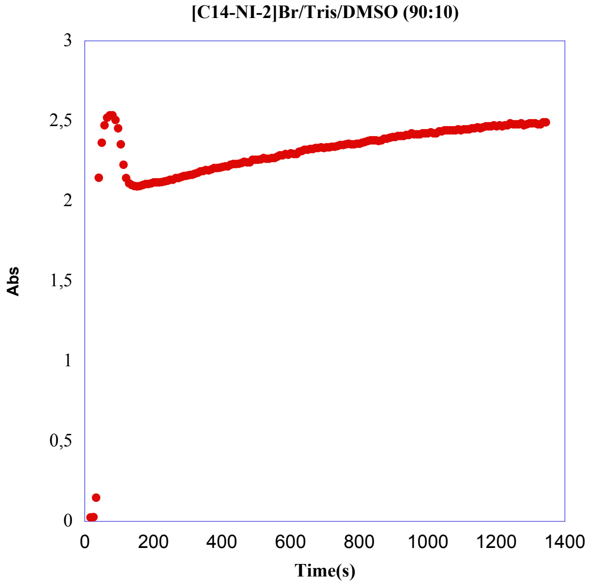
[C14-NI-2]Br/Tris/DMSO (90:10)
Abs
Time(s)
3
2,5
2
1,5
1
0,5
0
0
200
400
600
800
1000
1200
1400

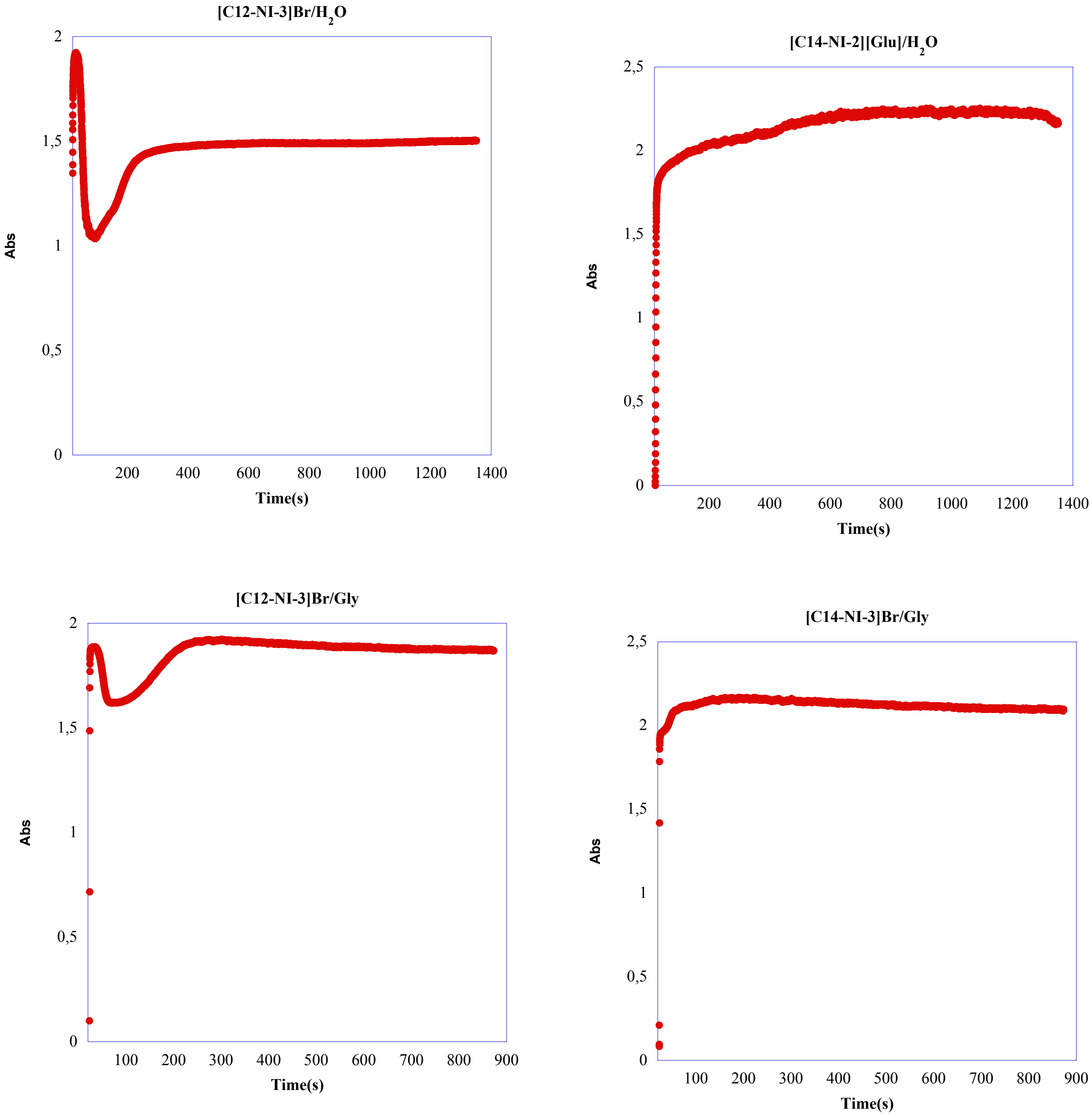

Figure S6. Plot of opacity, measured at λ = 568 nm, as a function of time for different hydrogels at 4% wt.

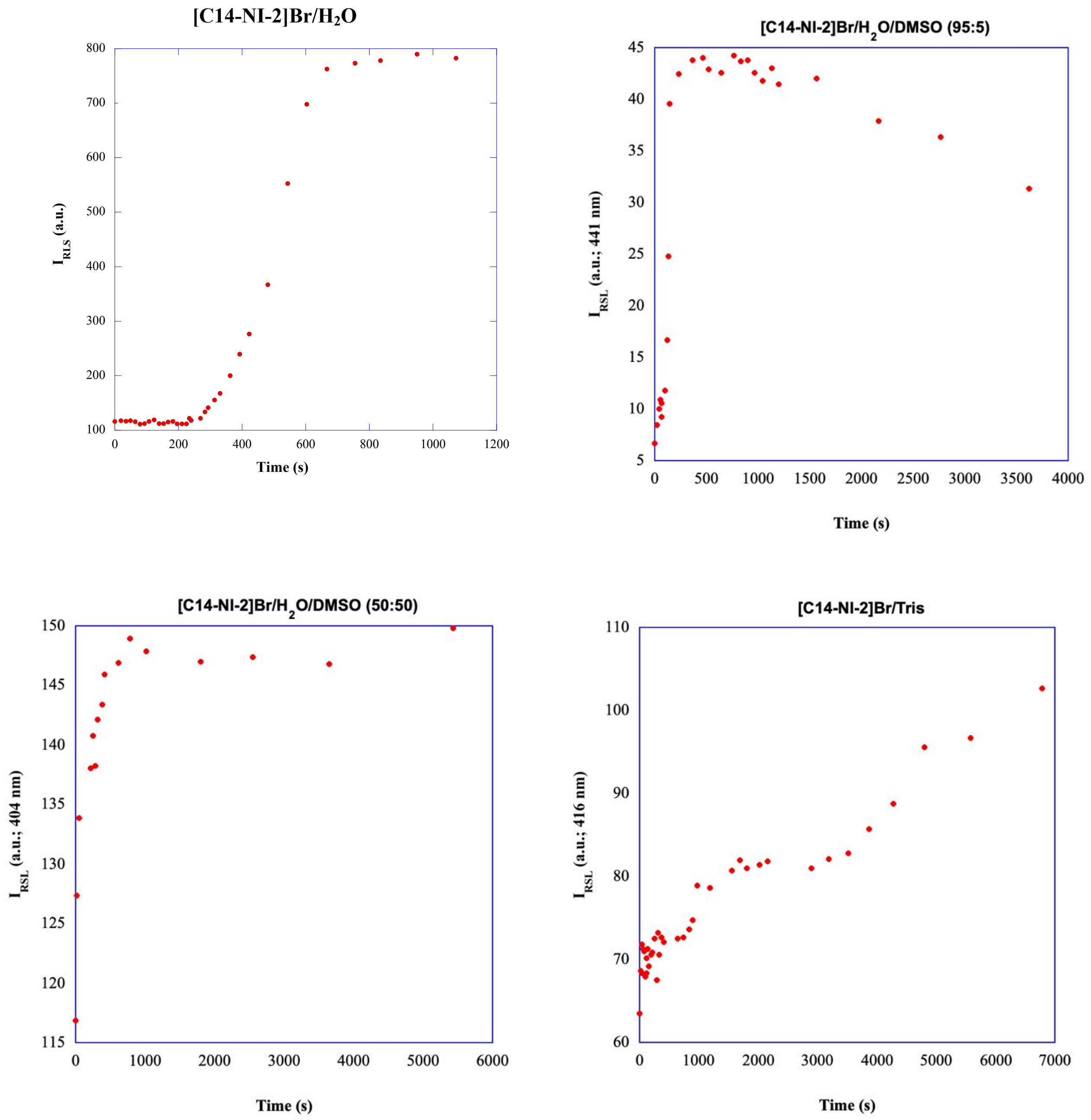
[C14-NI-2]Br/H2O
IRLS (a.u.)
Time (s)
[C14-NI-2]Br/H2O/DMSO (95:5)
IRSL (a.u.; 441 nm)
Time (s)
[C14-NI-2]Br/H2O/DMSO (50:50)
IRSL (a.u.; 404 nm)
Time (s)
[C14-NI-2]Br/Tris
IRSL (a.u.; 416 nm)
Time (s)

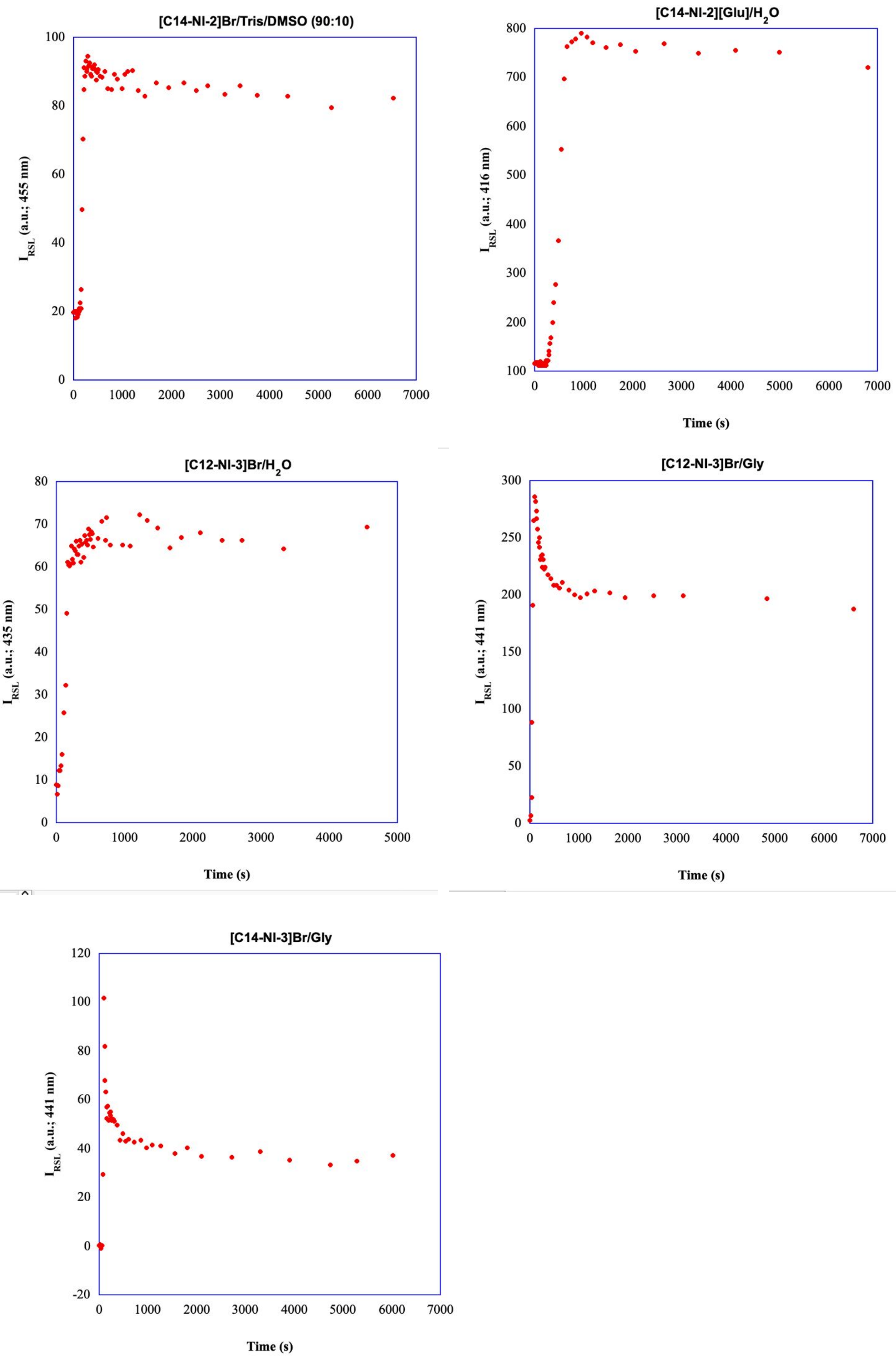

Figure S7. Plot of I_{RLS} as a function of time for different hydrogels at 4% wt.

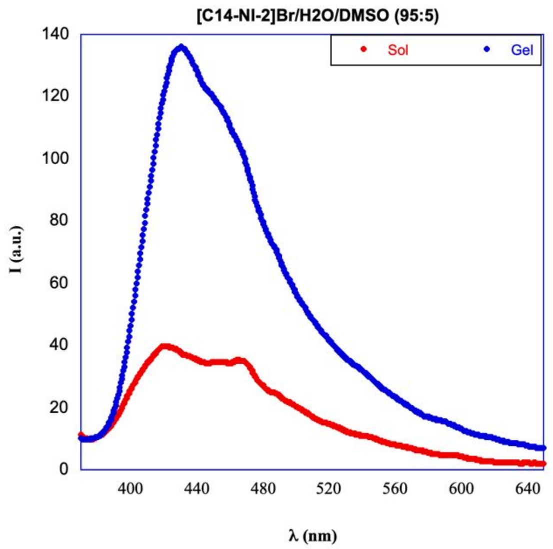
[C14-NI-2]Br/H2O/DMSO (95:5)
Sol
Gel
I (a.u.)
λ (nm)

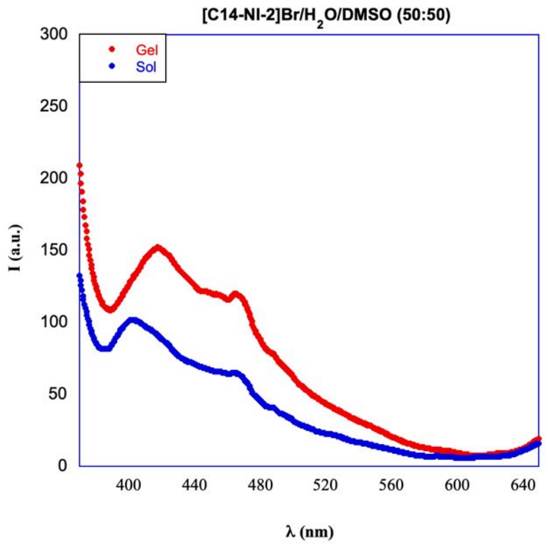
[C14-NI-2]Br/H$_2$O/DMSO (50:50)
Gel
Sol
I (a.u.)
λ (nm)

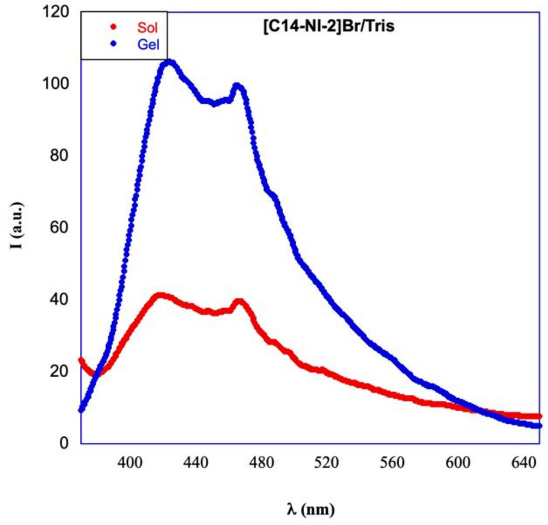
[C14-NI-2]Br/Tris
Sol
Gel
I (a.u.)
λ (nm)

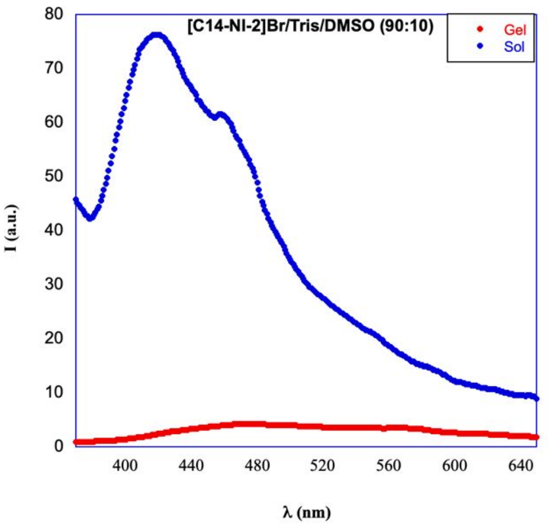
[C14-NI-2]Br/Tris/DMSO (90:10)
Gel
Sol
I (a.u.)
λ (nm)

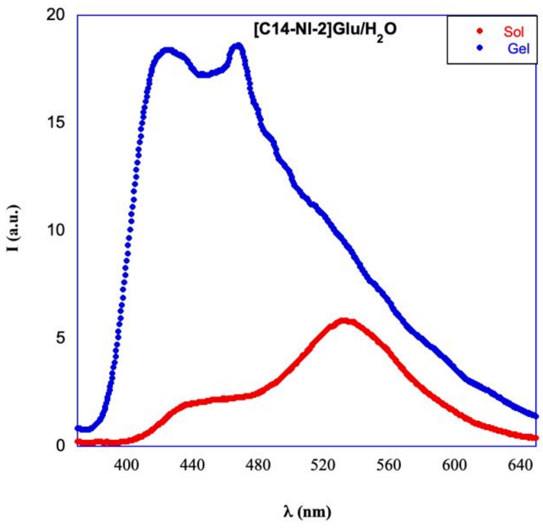
[C14-NI-2]Glu/H$_2$O
Sol
Gel
I (a.u.)
λ (nm)

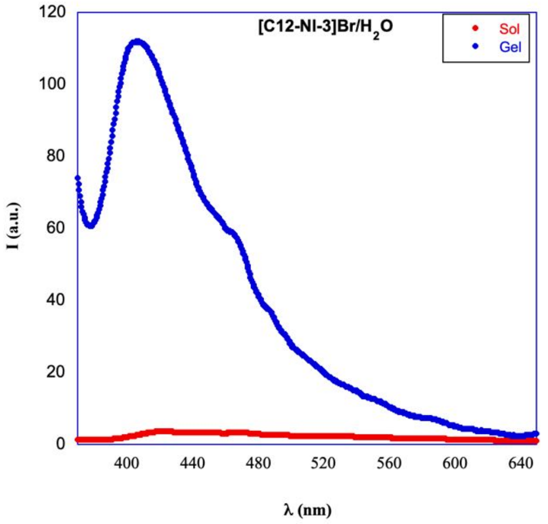
[C12-NI-3]Br/H$_2$O
Sol
Gel
I (a.u.)
λ (nm)

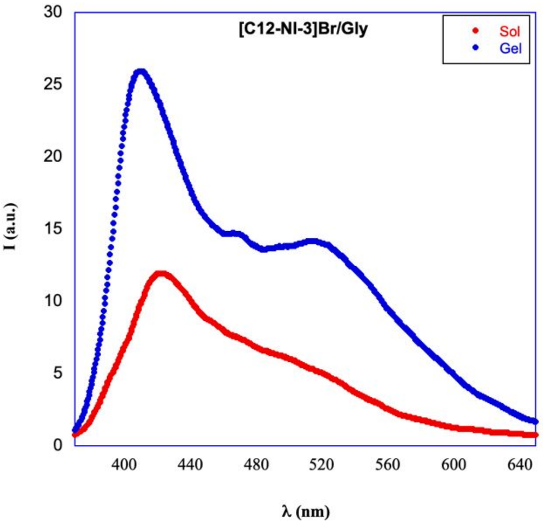

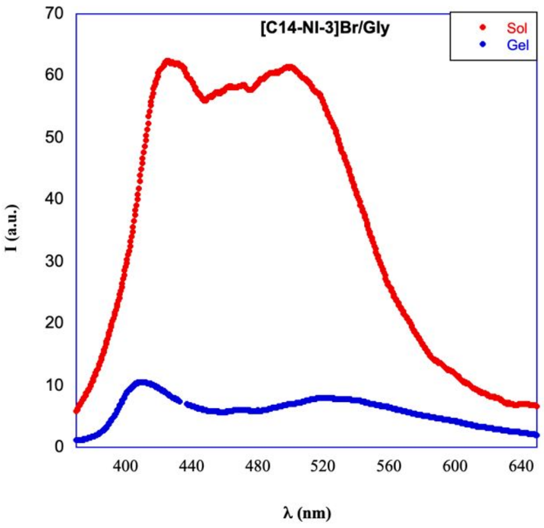

Figure S8. Emission spectra of hot solutions and gel phases 4% wt.

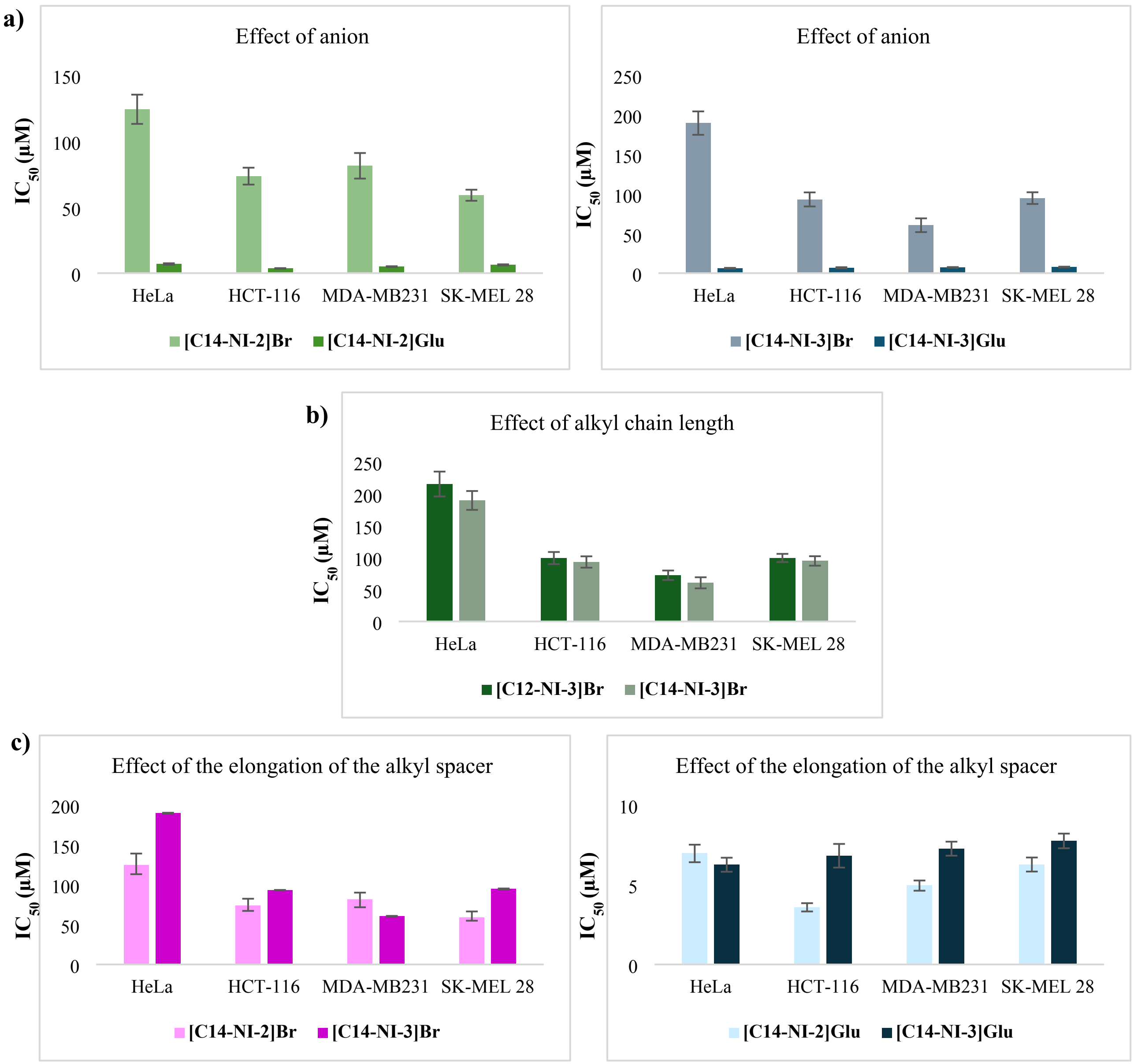

Figure S9. Histograms showing the effect of structural changes of investigated organic salts on cytotoxicity toward Hela, HCT-116, MDA-MB231 and SK-MEL 28 cancer cell lines. IC_{50} values were calculated after 24 h of treatment from the dose-response model and expressed in µM ±SD (standard deviation).

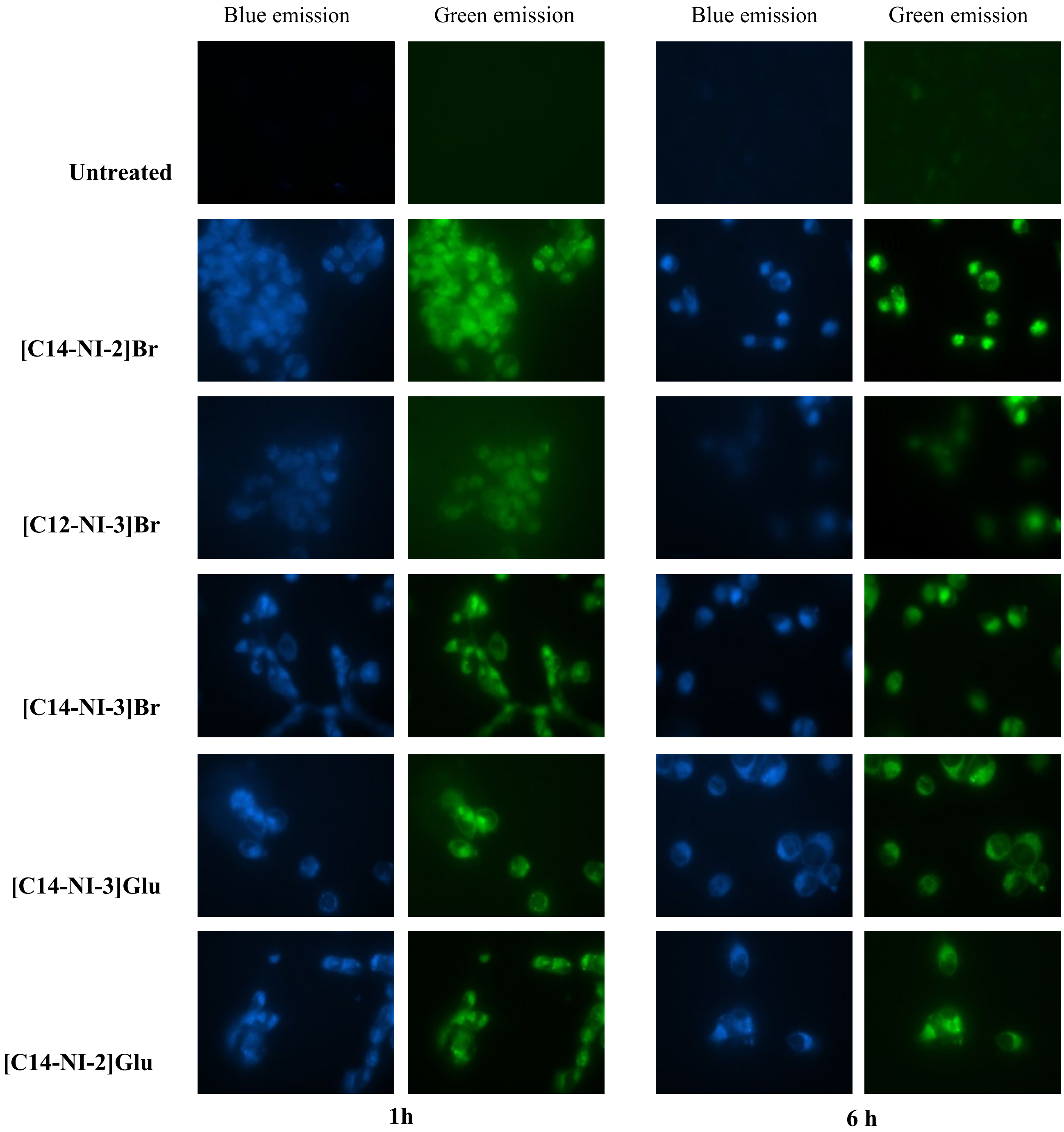

Figure S10. Fluorescence micrographs of MDA-MB231 cells after 1 h and 6 h of treatment with the IC_{50} concentration of each salt (excitation range 300 ms). Magnification 630×.

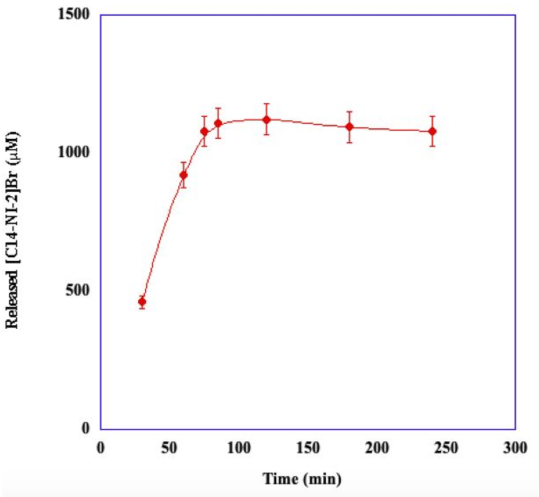

Figure S11. Plot of gelator release as a function of time for the [**C14-NI-2]Br** hydrogel in contact with 25 mL of PBS.

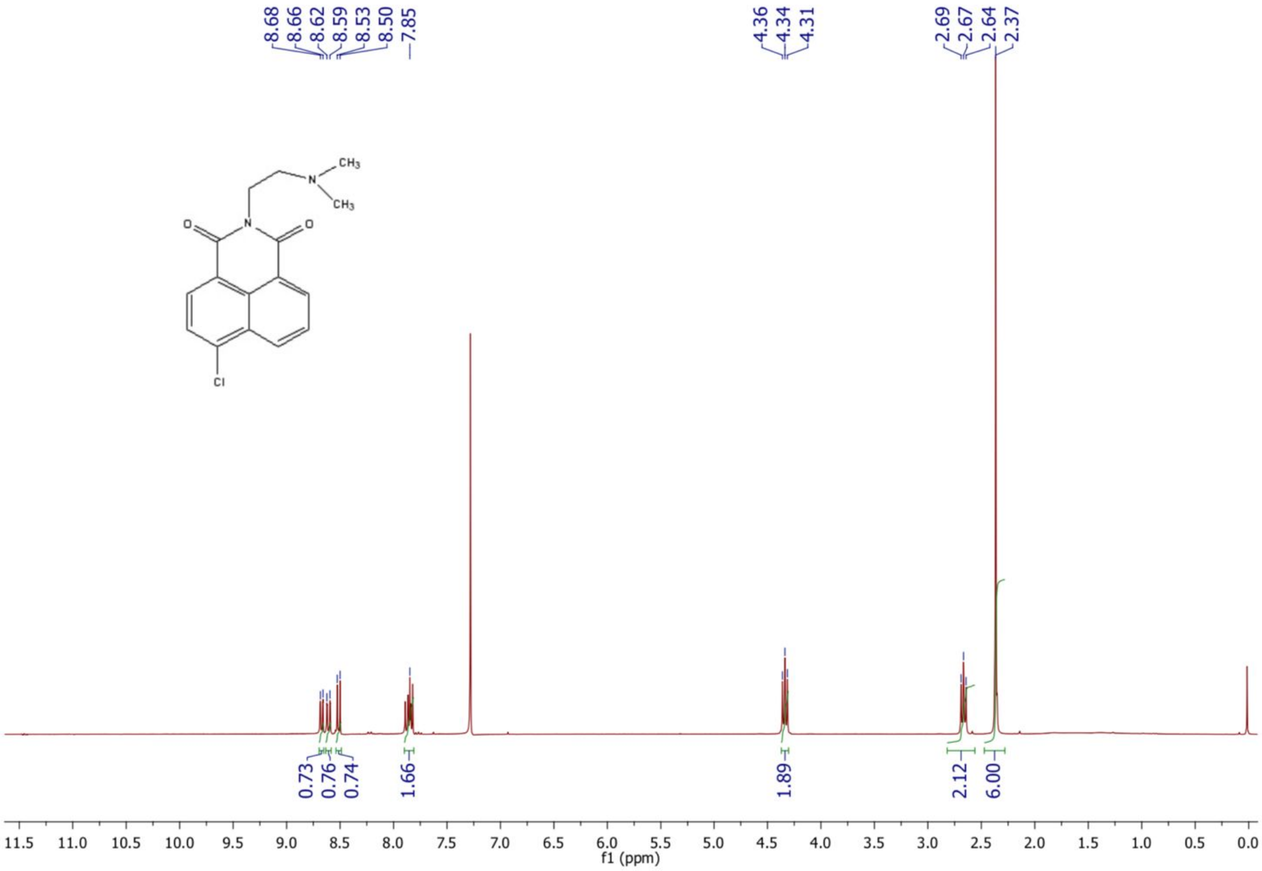

8.68
8.66
8.62
8.59
8.53
8.50
7.85
4.36
4.34
4.31
2.69
2.67
2.64
2.37
CH3
N
CH3
O
N
O
Cl
0.73
0.76
0.74
1.66
1.89
2.12
6.00
11.5 11.0 10.5 10.0 9.5 9.0 8.5 8.0 7.5 7.0 6.5 6.0 5.5 5.0 4.5 4.0 3.5 3.0 2.5 2.0 1.5 1.0 0.5 0.0
f1 (ppm)

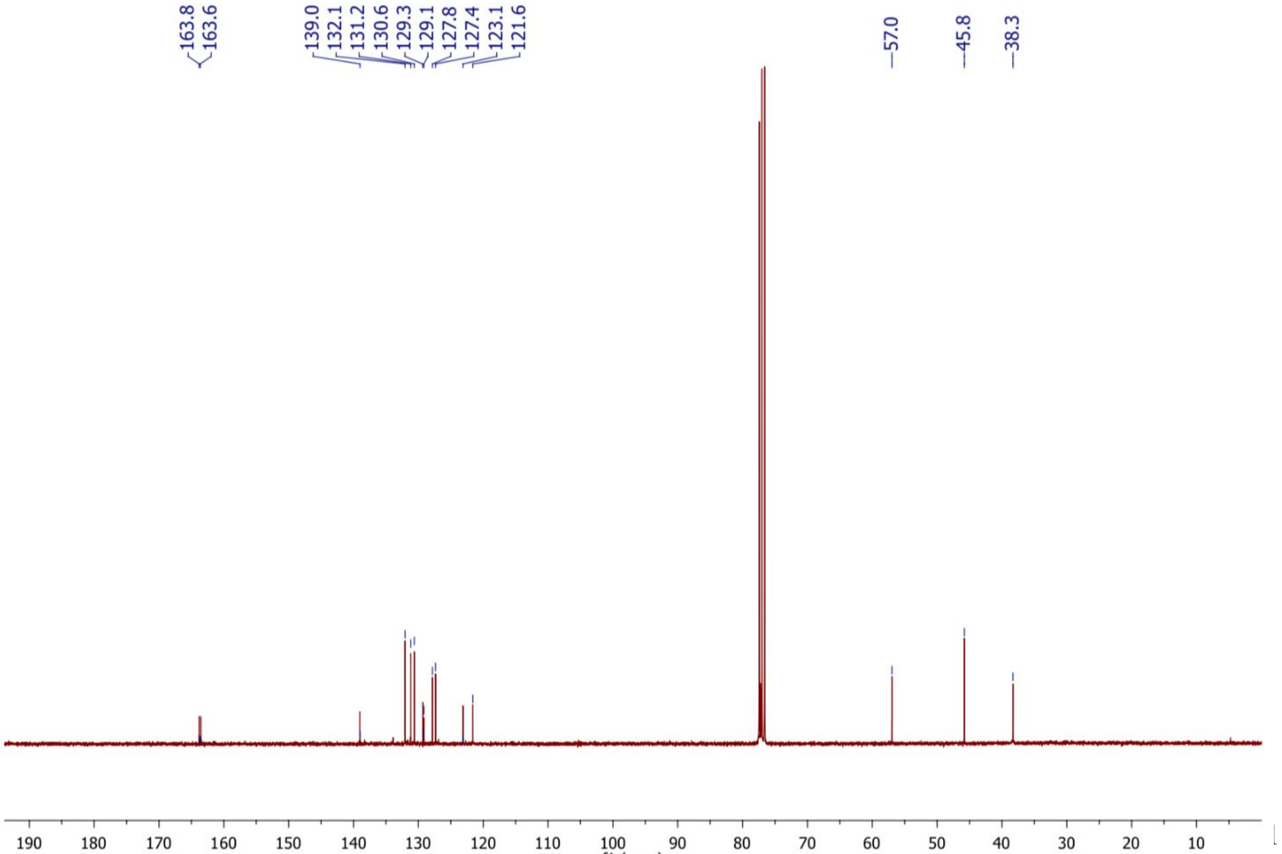

163.8
163.6
139.0
132.1
131.2
130.6
129.3
129.1
127.8
127.4
123.1
121.6
57.0
45.8
38.3
190 180 170 160 150 140 130 120 110 100 90 80 70 60 50 40 30 20 10
f1 (ppm)

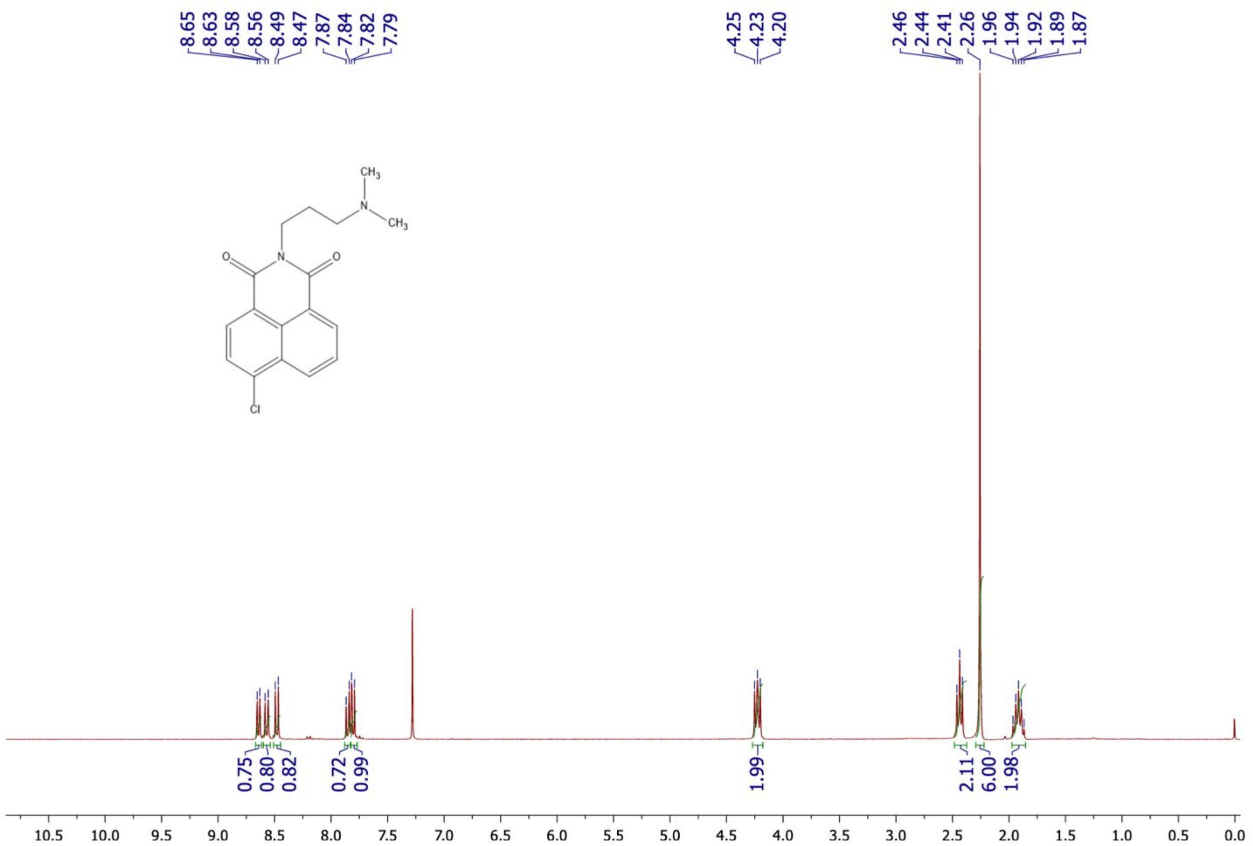
8.65
8.63
8.58
8.56
8.49
8.47
7.87
7.84
7.82
7.79
4.25
4.23
4.20
2.46
2.44
2.41
2.26
1.96
1.94
1.92
1.89
1.87
CH3
N
CH3
O
N
O
Cl
0.75
0.80
0.82
0.72
0.99
1.99
2.11
6.00
1.98
10.5
10.0
9.5
9.0
8.5
8.0
7.5
7.0
6.5
6.0
5.5
5.0
4.5
4.0
3.5
3.0
2.5
2.0
1.5
1.0
0.5
0.0

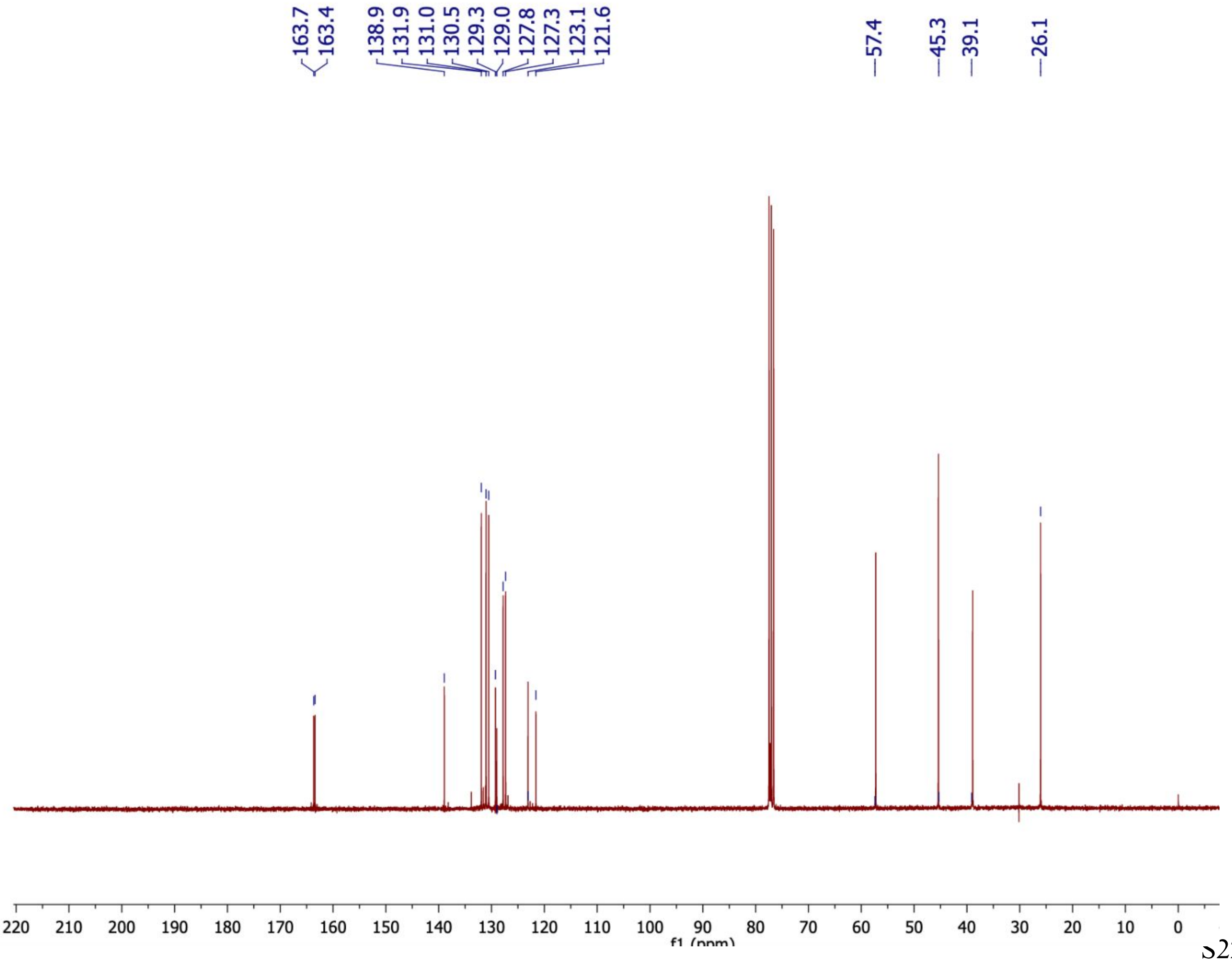
163.7
163.4
138.9
131.9
131.0
130.5
129.3
129.0
127.8
127.3
123.1
121.6
57.4
45.3
39.1
26.1
220
210
200
190
180
170
160
150
140
130
120
110
100
90
80
70
60
50
40
30
20
10
0
f1 (ppm)

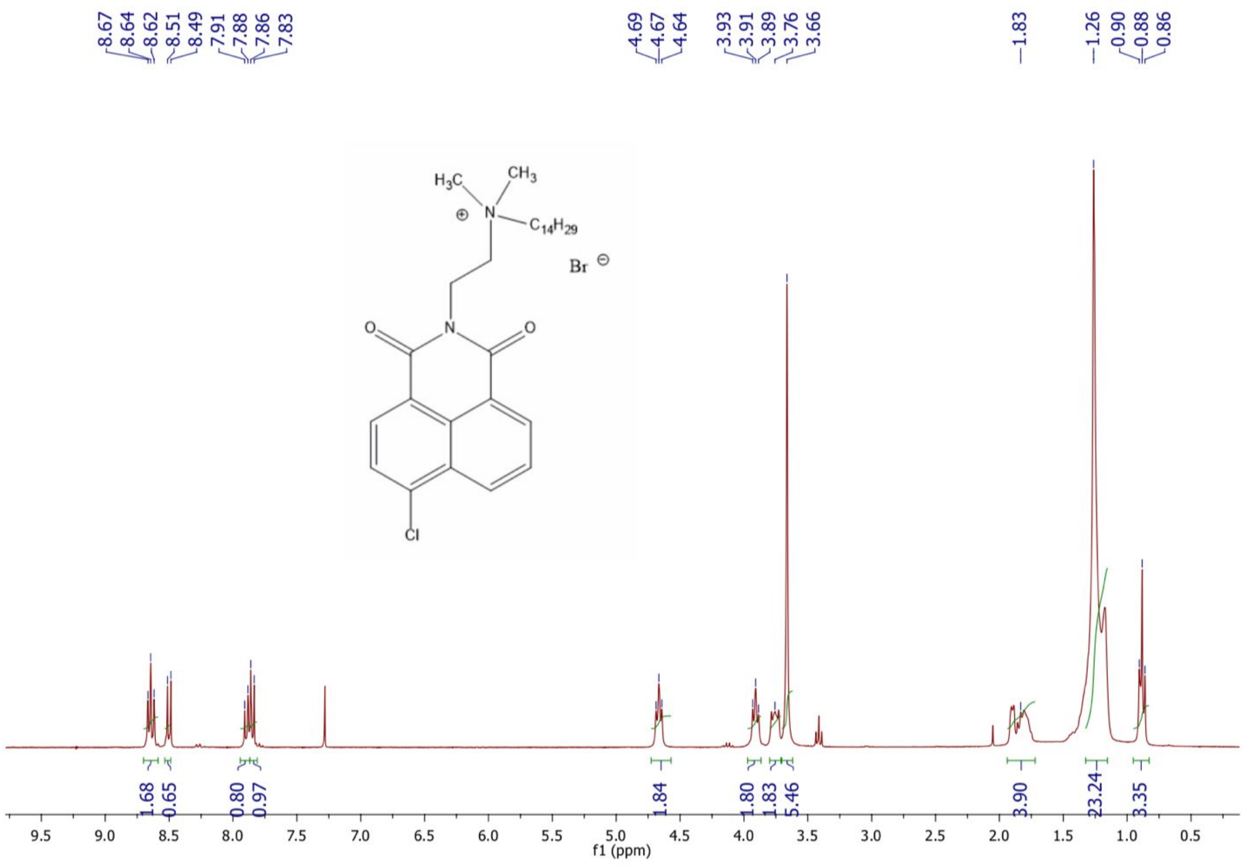
8.67
8.64
8.62
8.51
8.49
7.91
7.88
7.86
7.83
4.69
4.67
4.64
3.93
3.91
3.89
3.76
3.66
1.83
1.26
0.90
0.88
0.86
H3C
CH3
N
C14H29
Br
O
N
O
Cl
1.68
0.65
0.80
0.97
1.84
1.80
1.83
5.46
3.90
23.24
3.35
f1 (ppm)

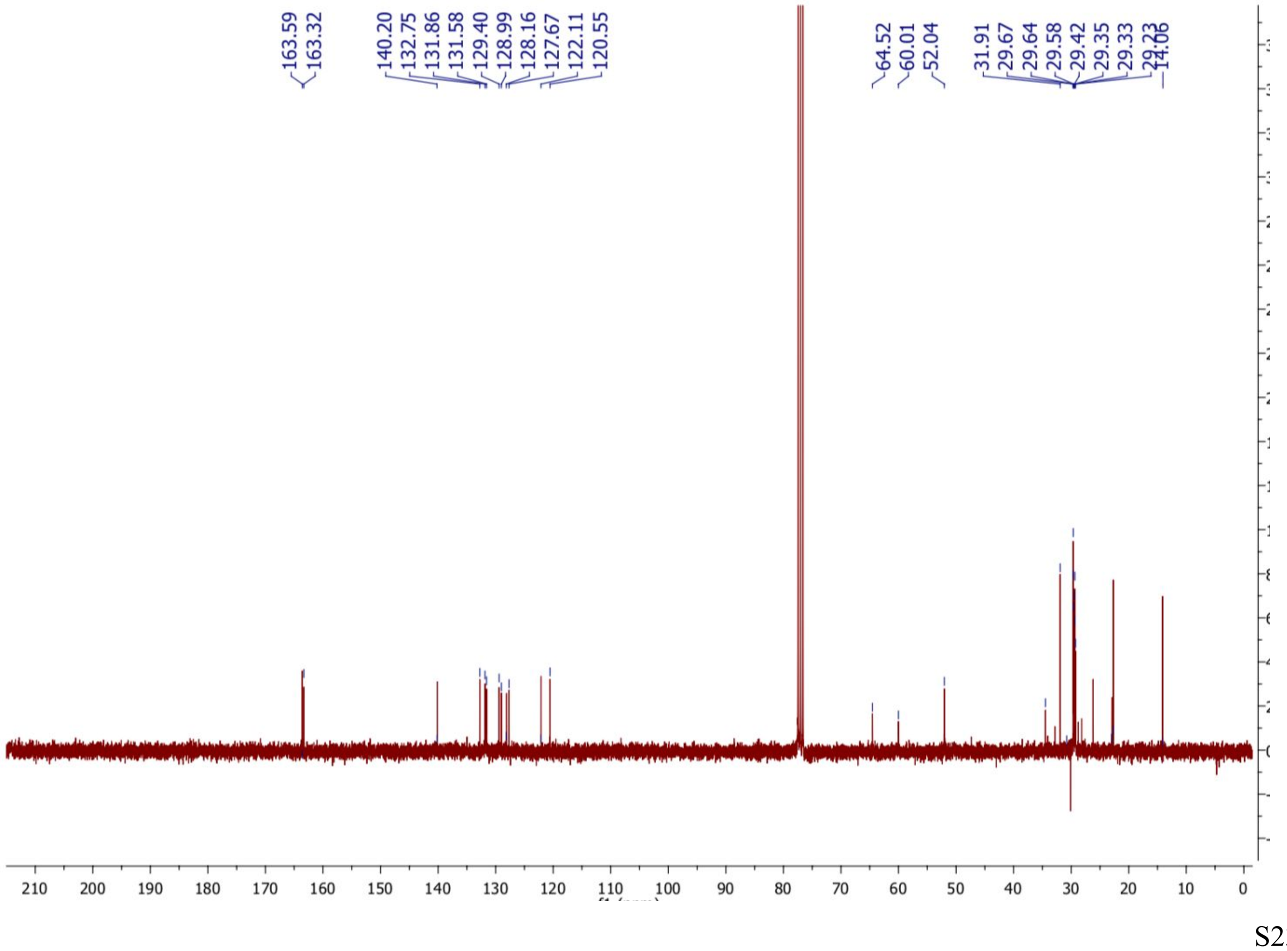
163.59
163.32
140.20
132.75
131.86
131.58
129.40
128.99
128.16
127.67
122.11
120.55
64.52
60.01
52.04
31.91
29.67
29.64
29.58
29.42
29.35
29.33
29.23
14.06

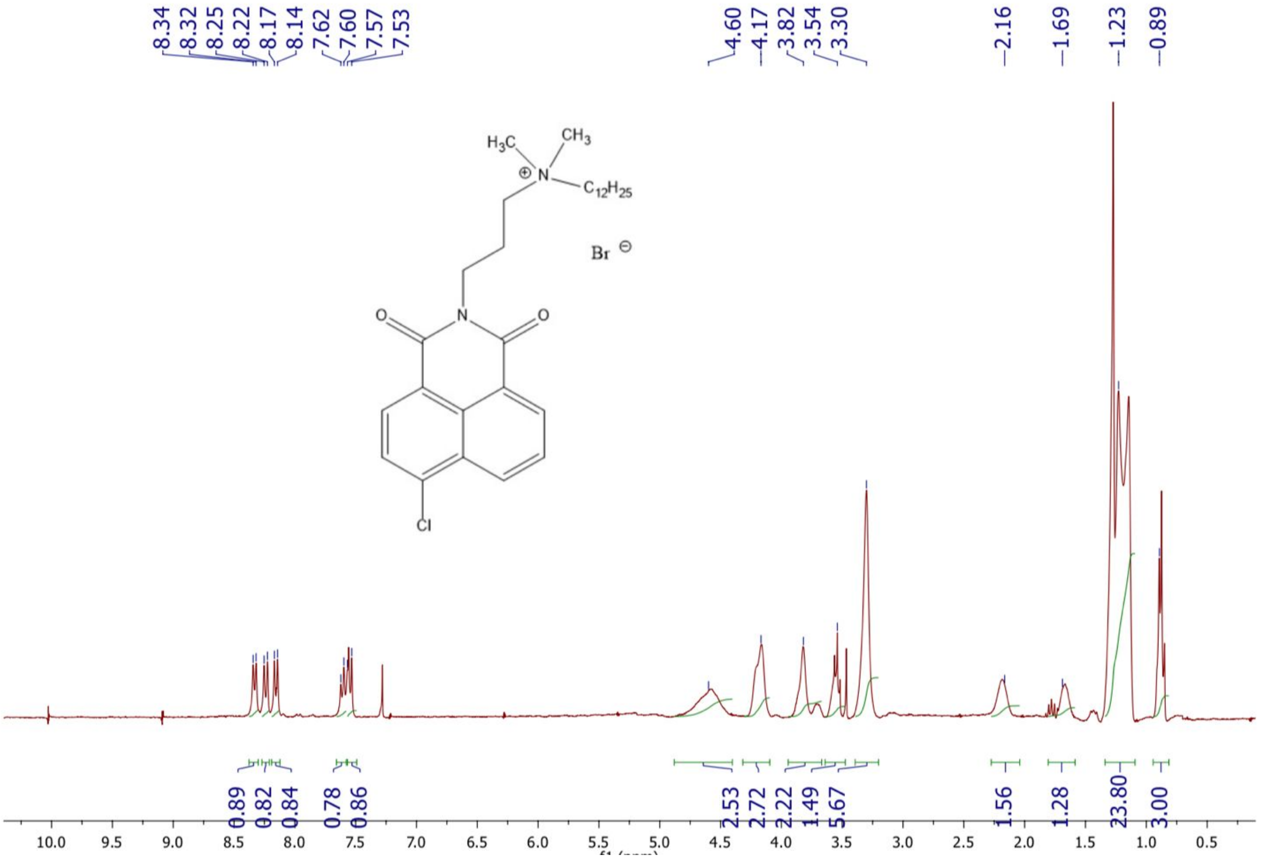
8.34
8.32
8.25
8.22
8.17
8.14
7.62
7.60
7.57
7.53
4.60
4.17
3.82
3.54
3.30
2.16
1.69
1.23
0.89
H3C
CH3
N
C12H25
Br
O
N
O
Cl
0.89
0.82
0.84
0.78
0.86
2.53
2.72
2.22
1.49
5.67
1.56
1.28
23.80
3.00
10.0
9.5
9.0
8.5
8.0
7.5
7.0
6.5
6.0
5.5
5.0
4.5
4.0
3.5
3.0
2.5
2.0
1.5
1.0
0.5

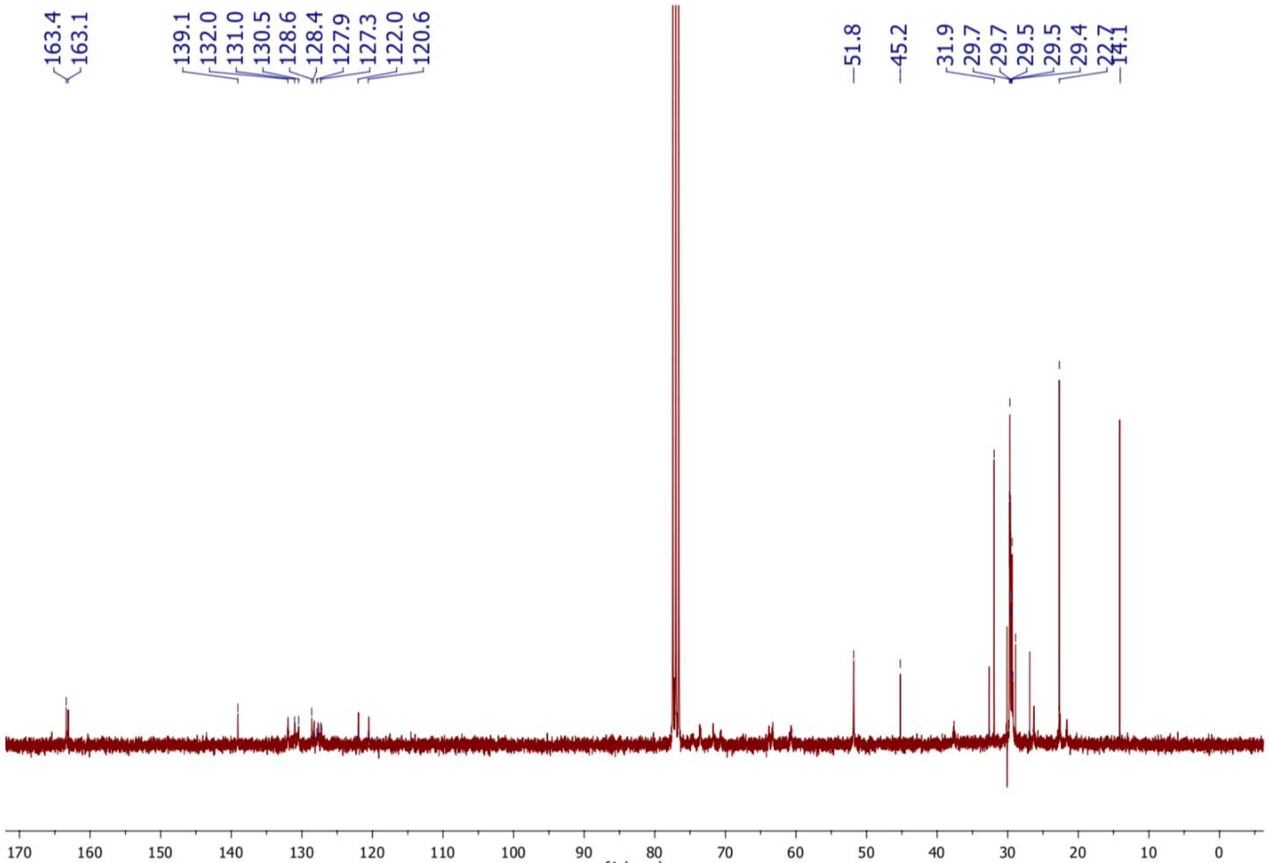
163.4
163.1
139.1
132.0
131.0
130.5
128.6
128.4
127.9
127.3
122.0
120.6
51.8
45.2
31.9
29.7
29.7
29.5
29.5
29.4
22.7
14.1
170
160
150
140
130
120
110
100
90
80
70
60
50
40
30
20
10
0
f1 (ppm)

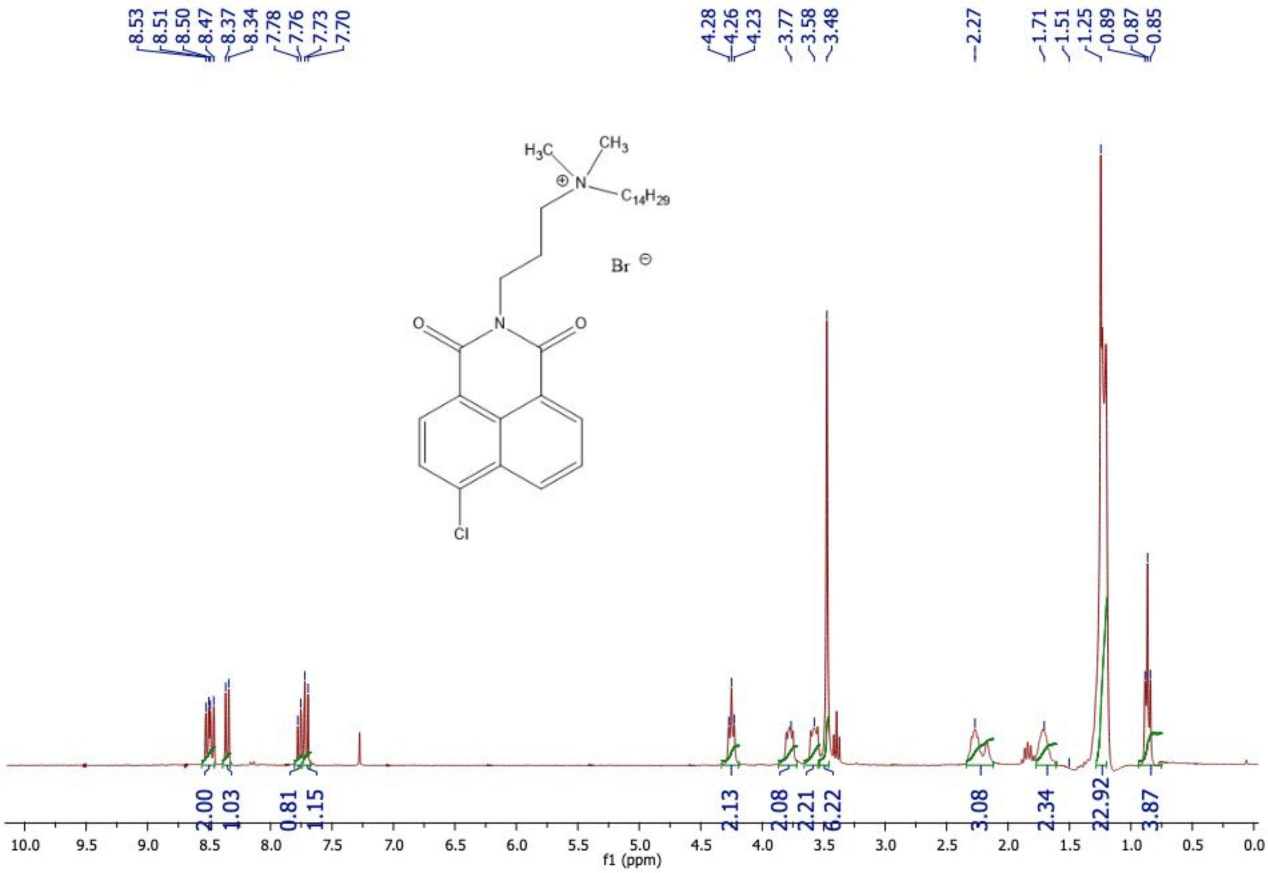
8.53 8.51 8.50 8.47 8.37 8.34 7.78 7.76 7.73 7.70
4.28 4.26 4.23 3.77 3.58 3.48
2.27
1.71 1.51 1.25 0.89 0.87 0.85
H3C CH3 N C14H29
Br
O N O
Cl
2.00 1.03 0.81 1.15 2.13 2.08 2.21 6.22 3.08 2.34 22.92 3.87
10.0 9.5 9.0 8.5 8.0 7.5 7.0 6.5 6.0 5.5 5.0 4.5 4.0 3.5 3.0 2.5 2.0 1.5 1.0 0.5 0.0
f1 (ppm)

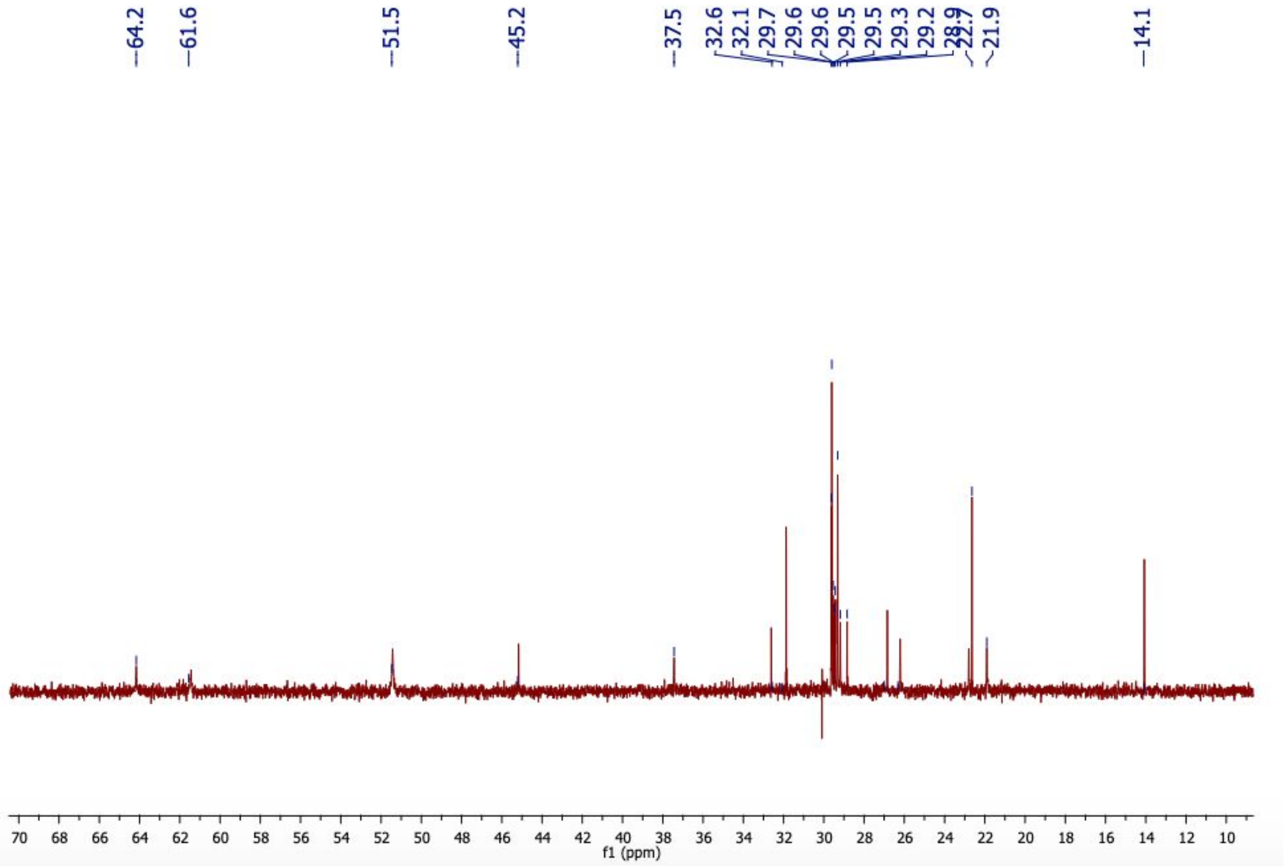
64.2
61.6
51.5
45.2
37.5
32.6 32.1 29.7 29.6 29.6 29.5 29.5 29.3 29.2 28.9 22.7 21.9
14.1
70 68 66 64 62 60 58 56 54 52 50 48 46 44 42 40 38 36 34 32 30 28 26 24 22 20 18 16 14 12 10
f1 (ppm)

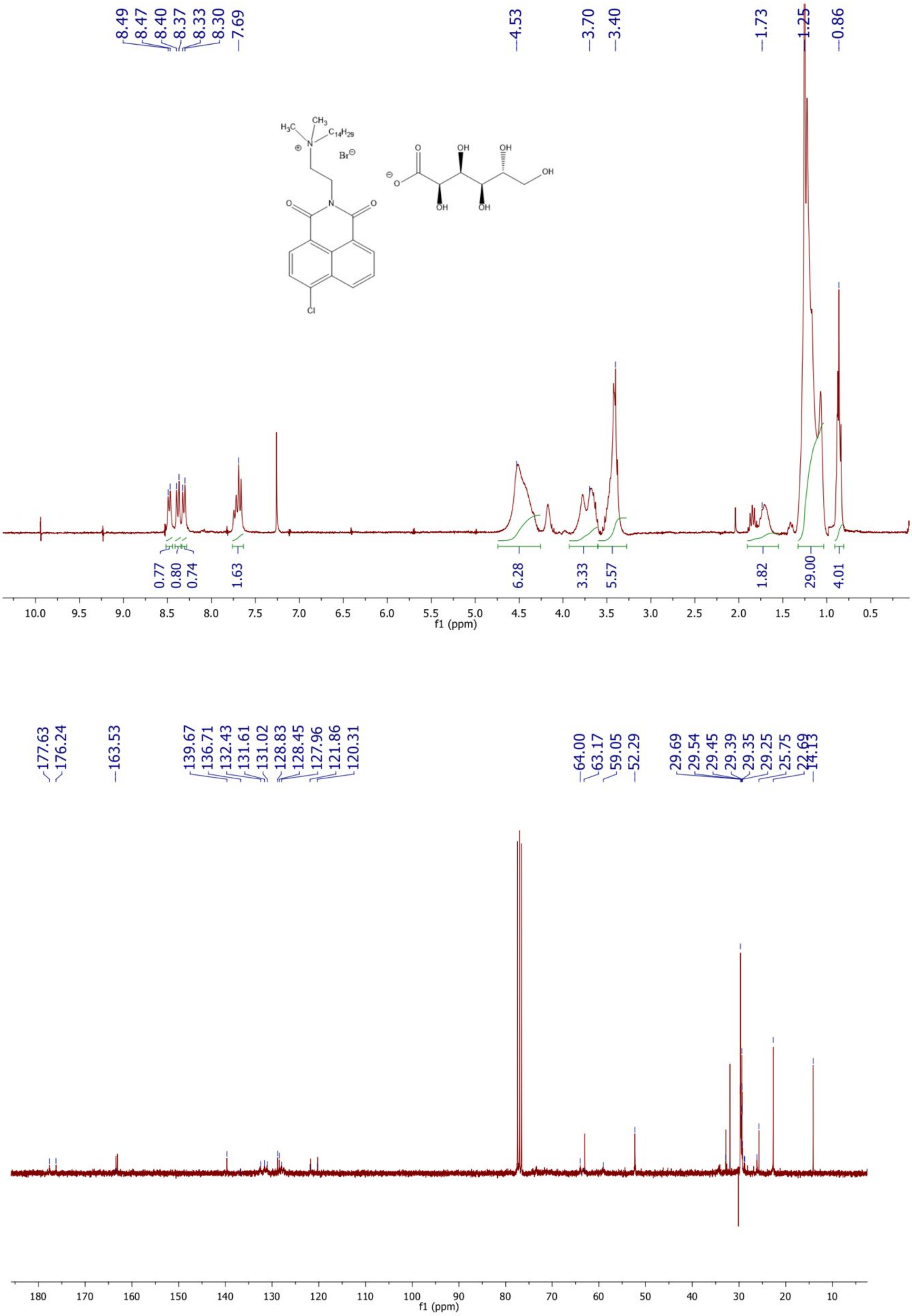

Figure S12. ^{1}H and ^{13}C NMR spectra of precursors and salts.

Table S1. Position of main absorption and emission band for organic salts as a function of the solvent nature

Salt	Solvent	λ_{max} (nm) UV-vis	λ_{max} (nm) Emission	I_{em} (a.u.)
**[C14-NI-2]Br**	H_2O	344	426	387
	H_2O/DMSO (95: 5)	344	441	235
	H_2O/DMSO (50: 50)	342	404	67
	DMSO	338	394	36
	Tris (1x)	342	456	330
	Tris (1x)/DMSO (90:10)	342	445	160
**[C14-NI-2][Glu]**	H_2O	343	405	6790
	DMSO	339		
**[C14-NI-3]Br**	H_2O	343	407	6800
	DMSO	337	418	284
**[C12-NI-3]Br**	H_2O	344	414	6350
	DMSO	339	391	172

Table S2. Emission quantum yield for organic salts in water solution, measured using 9,10-phenantroline in ethanol as standard.

Organic salt	ϕ
**[C14-NI-2]Br**	0.40
**[C14-NI-2][Glu]**	0.16
**[C12-NI-3]Br**	0.15
**[C14-NI-3]Br**	0.45

Table S3. Gelation tests for **[C14-NI-2]Br** and **[C14-NI-3]Br**

S = solution; P = precipitate; PG = gelatinous precipitate; G = gel

	**[C14-NI-2]Br**		**[C14-NI-3]Br**	
Solvent	***Range (w/w; %)***		***Range (w/w; %)***	
H_2O	1-2	G	1-4	PG
H_2O/DMSO (95:5)	1-3.5	G	1-4	P
H_2O/DMSO (90:10)	1-3	PG	1-3.5	P
H_2O/DMSO (50:50)	1-2	G	1-4	PG
Tris (1x)	1-4	G	1-3	P
Tris (1x)/DMSO (90:10)	1-2.5	G		
Gly	1	G	1-3	G

Table S4. Gelation tests for **[C12-NI-3]Br** and **[C14-NI-3][Glu]**

	**[C12-NI-3]Br**		**[C14-NI-3][Glu]**	
Solvent	***Range (w/w; %)***		***Range (w/w; %)***	
H_2O	2	G	4	S
H_2O/DMSO (95:5)	1-2	PG	2-4	S
H_2O/DMSO (90:10)	2	PG	2-4	S
H_2O/DMSO (50:50)	2	P		
Tris (1x)	3	P		
Tris (1x)/DMSO (90:10)	2	P		
Gly	2	G	2-4	PG

S = solution; P = precipitate; PG = gelatinous precipitate; G = gel

Table S5. Thixotropy and sonotropy tests performed on gel phases at 4% (w/w).

Gel	Thixotropy Test	Sonotropy Test
[C14-NI-2]Br/Gly	Yes	Stable
[C14-NI-2]Br/H_2O	Stable	Stable
[C14-NI-2]Br/H_2O/DMSO (95:5; v/v)	Yes	Stable
[C14-NI-2]Br/H_2O/DMSO (50:50; v/v)	Yes	No
[C14-NI-2]Br/TRIS	Yes	Stable
[C14-NI-2]Br/TRIS/DMSO (90:10; v/v)	Yes	Stable
[C14-NI-3]Br/Gly	Stable	Stable
[C12-NI-3]Br/H_2O	No	Yes
[C12-NI-3]Br/Gly	Stable	Stable
[C14-NI-2]Glu/H_2O	No	Stable

Yes: gel phase reforms after the action of the stimulus; No: gel phase does not reform after the action of the stimulus; Stable: gel phase is stable to the action of the stimulus;

Table S6. I_{RLS}, opacity values and gelation times for different gel phases at 4 wt %.

Gelator	Solvent	I_{RLS} (a.u.)	A_g	t_g (s) from UV-vis measurement
[C14-NI-2]Br	H_2O	52	1.98	28
[C14-NI-2]Br	H_2O/DMSO (95:5)	47	2.16	54
[C14-NI-2]Br	H_2O/DMSO (50:50)	143	1.11; 2.13	142
[C14-NI-2]Br	Tris (1x)	88	2.40	30
[C14-NI-2]Br	Tris 1x/DMSO (90:10)	90	2.10	140
[C14-NI-2]Br	Gly	202	2.10	87
[C12-NI-3]Br	H_2O	71	1.84; 1.45	420
[C12-NI-3]Br	Gly	215	1.82; 1.87	230
[C14-NI-3]Br	Gly	42	2.13	115
[C14-NI-2][Glu]	H_2O	760	2.00	30

Table S7. λ_{max} (nm) and emission intensity (I) of hot solutions and corresponding gel phases at 4% wt.

		Hot Solution		Gel phase		
Gelator	Solvent	λ_{max} (nm)	I (a.u.)	λ_{max} (nm)	I (a.u.)	Shift (nm)
[C14-NI-2]Br	H_2O	416.5	53.0	465.5	90	-1.5
		**467.0**	41			
[C14-NI-2]Br	H_2O/DMSO	**422.0**	40	430.5	136	8.0
	(95:5)	465.0	35			
[C14-NI-2]Br	H_2O/DMSO	402.0	102	417.5	152	15.5
	(50:50)	464.0	65	464.5	120	0.5
[C14-NI-2]Br	TRIS	420.0	41	423.5	106	3.5
		466.0	40	465.5	100	0.5
[C14-NI-2]Br	TRIS/DMSO	417.0	76	420.0	4	3.0
	(90:10)					
[C14-NI-2]Br	Gly	427.5	63	421.0	290	-6.5
[C12-NI-3]Br	H_2O	421.5	4	407.0	112	-14.5
[C12-NI-3]Br	Gly	421.5	12	410.0	26	-11.5
[C14-NI-3]Br	Gly	425.5	62	409.5	11	-16.0
		500.0	61	519.5	8	+ 19.5 nm
[C14-NI-2][Glu]	H_2O	533.0	6	469	18	-54
